# Oleanane-Type Triterpene Conjugates with 1*H*-1,2,3-Triazole Possessing of Fungicidal Activity

**DOI:** 10.3390/molecules27154928

**Published:** 2022-08-02

**Authors:** Zili Chen, Yu Jiang, Chen Xu, Xiangyu Sun, Chao Ma, Zihao Xia, Hanqing Zhao

**Affiliations:** 1 Key Laboratory for Northern Urban Agriculture of Ministry of Agriculture and Rural Affairs, Beijing University of Agriculture, Beijing 102206, China; chenzili1995@163.com (Z.C.); jy1013937462@126.com (Y.J.); 13691458370@163.com (C.X.); 2 Torch High Technology Industry Development Center, Ministry of Science &Technology, Beijing 100045, China; sunxiangyu1990@126.com; 3 Beijing Zhong Bao Green Agriculture Technology Group Co., Ltd., Beijing 100193, China; racoon_happy@126.com

**Keywords:** oleanolic acid, triazoles, fungicides, new pesticides

## Abstract

The triazole pesticide is an organic nitrogen-containing heterocyclic compound with a 1,2,3-Triazole ring. In order to develop a potential glucosamine-6-phosphate synthase (GlmS) inhibitor bactericide, 18 triazole-derivative compounds were synthesized efficiently. In addition, these compounds have not been reported in the literature. The structure was confirmed by high-resolution mass spectrometry (HRMS), ${}^{1}$H NMR and ${}^{13}$C NMR. The potential use of the most promising derivatives has been investigated by testing their antifungal activity and enzyme inhibitory activity, revealing inhibitory activities in the low micromolar range. Among them, the antifungal effects of compounds **1e**, **1f**, **1g**, **2e**, **2f**, and **2g** on ***Sclerotinia sclerotiorum*** were particularly significant, all of which were above 83%. These compounds will be further investigated as potential antifungal lead compounds. Their structure–activity relationships are discussed based on the effects of substituted phenyl groups on compounds.

## 1. Introduction

**Figure 1:**
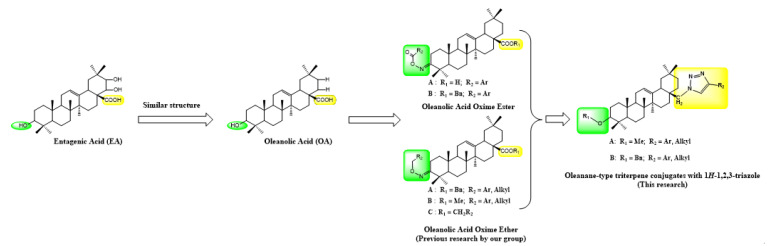
The structural formula of Entagenic Acid, Oleanolic Acid, oleanolic acid oxime ester/oleanolic acid oxime ether and Oleanane-type triterpene conjugates with 1*H*-1,2,3-Triazole.

The development of lead compounds of new pesticides based on natural products plays an important role in the field of pesticide research [1,2]. Many natural compound derivatives have shown strong vitality as pesticides. There are a wide range of natural compounds that are biologically diverse and unique in their role. Moreover, they are easily degradable and compatible with the environment [3]. The newly developed fungicide based on natural products has the advantages of safety, high efficiency, low residue and low resistance to chemicals. It also ensures the high yield of agricultural products such as vegetables in addition to solving the problem of pesticide residues. At present, 1,2,3-Triazole and its derivatives have not been discovered in nature and can only be synthesized by artificial means [4]. The 1,2,3-Triazole structure in which three nitrogen atoms are arranged adjacently to eachother in the five-membered ring was first reported by Michael in 1893 [5]. For a long time, the synthesis and application of triazole rings have received great attention from scientific researchers in various fields at home and abroad. An abundance of research has shown that this class of compounds bears significant anti-inflammatory effects [6]. Further, studies have shown bacteriostatic [7], anti-tumor [8,9], and anti-HIV effects [10,11] in medical applications; and this type of compound is widely used in fungicides [12], herbicides [13], and insecticides [14] in agriculture. In addition, such compounds have shown very important applications in the fields of organic synthesis, medicinal chemistry, organometallic catalysis, and materials science [15,16,17]. Rational design based on specific biological targets is one of the important means of developing new pesticides internationally. According to the literature, entagenic acid (EA, Figure 1) has good inhibitory activity on GlmS [18], which has been verified by molecular docking experiments. The researchers used oleanolic acid which has high structural similarity to entagenic acid as raw material, the oleanolic acid oxime ester and oleanolic acid oxime ether compounds were synthesized and their biological activities were determined [19,20,21,22]. Subsequently, some target compounds exhibited certain inhibitory activity and good antifungal activity against GlmS. This experiment will continue to develop new glucosamine-6-phosphate synthase inhibitors.

**Scheme 1:**
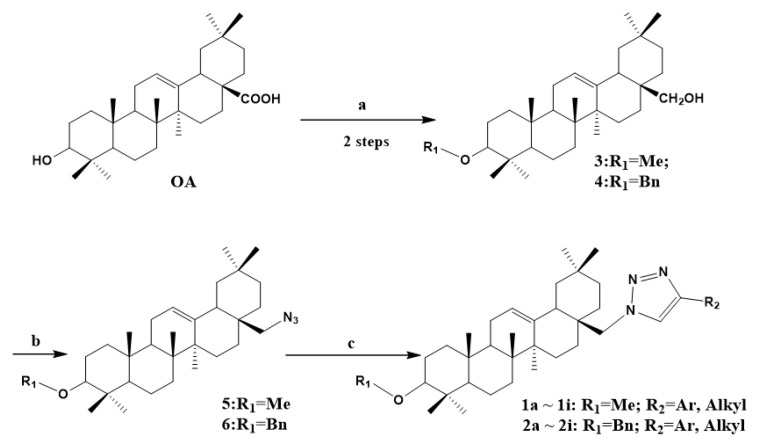
Synthesis of the target compounds **1** and **2**. Reagents and conditions: step a: (1) MeI/ BnBr, NaH, THF, 0 ${}^{\circ }$C to 40 ${}^{\circ }$C, 12 h; (2) LAH, THF, 0 ${}^{\circ }$C to room temperature, 4 h; 75% for **3**, 72% for **4**; step b: Under N${}_{2}$ protection, Tf${}_{2}$O, pyridine, 0 ${}^{\circ }$C to room temperature, 15 min, then NaN${}_{3}$, DMF, 60 ${}^{\circ }$C; 87% to **5**, 89% to **6**; step c: CuSO${}_{4}$·5H${}_{2}$O, sodium ascorbate, then substituted phenylacetylene or alkyl alkyne, CH${}_{3}$OH:H${}_{2}$O = 1:1, room temperature, 1 h, 77–86% for **1a∼1i**, 74–89% for **2a∼2i**.

Based on the results accumulated in the early stages of these experiments, various important biological activities possessed by the triazole group were considered. In this paper, a novel hexosaminidase (GlmS) inhibitor oleanane-type conjugate with 1,2,3-Triazole fragment was designed and synthesized followed by the determination of biological activity of the target compound and inhibition of *Candida albicans* GlcN-6-P synthase. We hope to report the synthesis (Scheme 1) and biological activity in more detail, as well as the structure–activity relationship studies. We report the preliminary results of the study here.

## 2. Results and Discussion

As shown in Scheme 1, we envisioned that the target compounds, namely triazole derivatives **1** and **2** could be synthesized from the intermediates **3**, **4**, **5** and **6**. According to known methods, the first three steps can be prepared using oleanolic acid as a starting material [23,24]. Tf${}_{2}$O was added to compounds **3** and **4** under N${}_{2}$ protection for 15 min; and then reacted with sodium azide to form intermediate compounds **5** and **6**. Then cyclization of **5** and **6** with an alkyne provided the desired triazole derivatives **1** and **2** under the conditions of copper sulfate and sodium ascorbate in high yields, respectively.

All the derivatives were synthesized according to the procedures described in Scheme 1 with good overall yields of 70–89%. The synthesized compounds were characterized by ${}^{1}$H-NMR, ${}^{13}$C-NMR, and HRMS (Supporting information). We used CDCl${}_{3}$ as a solvent to confirm the structure of compounds by NMR. The physical data of the target compounds are given in Table 1.

### 2.1. Antifungal Activity of Series of Compounds ***1*** and ***2*** against Six Fungi

Fungicidal activities of the target compounds **1** and **2** against six fungal species were evaluated as previously reported [25] and compared with the commercial fungicide chlorothalonil. As shown in Table 2, the resulting data revealed that most of the tested compounds displayed a certain degree of fungicidal activity against the six species. The determination results of antifungal activity exhibited that the oleanane-type conjugates with 1,2,3-Triazole fragment showed some antifungal activity against six kinds of pathogenic fungi at a concentration of 50 μg/mL.

In general, the following structure–activity relationships (SAR) are observed in series of compounds **1** and **2**: (1) As a whole, the target compound had a certain inhibitory effect on the tested strains, all compounds inhibited ***Sclerotinia sclerotiorum***, ***Botrytis cinerea Pers*** and ***Rhizoctonia solani Kuhn*** at 50 μg/mL, and the inhibition rate was above 50%. (2) The antifungal effects of compounds **1e**, **1f**, **1g**, **2e**, **2f** and **2g** on ***Sclerotinia sclerotiorum*** were particularly prominent, 85.6%, 83.1%, 87.6%, 86.8%, 87.7%, 89.6%, respectively. (3) According to compound **1e** (R${}_{2}$ = 4-Cl-C${}_{6}$H${}_{4}$-), **1f** (R${}_{2}$ = 4-NO${}_{2}$-C${}_{6}$H${}_{4}$-), **1g** (R${}_{2}$ = 4-F-C${}_{6}$H${}_{4}$-), **2e** (R${}_{2}$ = 4-Cl-C${}_{6}$H${}_{4}$-), **2f** (R${}_{2}$ = 4-NO${}_{2}$-C${}_{6}$H${}_{4}$-), **2g** (R${}_{2}$ = 4-F-C${}_{6}$H${}_{4}$-), sterilization data show that increasing the electron-withdrawing ability of the substituent on the benzene ring can improve the fungicidal activity. (4) When R${}_{2}$ is a substituted phenyl group, the fungicidal activity of the series of compounds is superior to the compound with alkyl. (5) In general, compared to the series of compounds **1** and **2**, the latter (R${}_{1}$ = Bn) showed better fungicidal activity (R${}_{1}$ = Me) than the former.

### 2.2. Bioassay of Enzyme Inhibitory Activities

Inhibitory activities of all the synthesized compounds towards *Candida albicans* GlcN-6-P synthase were evaluated using the optimized Elson–Morgan method as previously reported [25]. The absorption value of the solution was measured at 585 nm, and then the concentration was measured using the specification curve which was determined by the relation between the absorption value and the concentration of glucosamine-6-phosphate. The inhibition rates are given in Table 3 at 0.35 mm.

The enzyme activity of compounds with a methyl substituent was slightly better than those possessing a benzyl substituent. However, this observation was not particularly obvious. When R${}_{2}$ was a halogen-substituted aryl (**1e**, **1g**), the inhibitory activity of the compound was better than that of others in the same series. Benzyl substituted compounds (**2**) also showed relatively good inhibitory activity.

## 3. Experimental Section

### 3.1. Reagents and Materials

Pyridine (Py), tetrahydrofuran (THF), methanol (MeOH), N, N-dimethylformamide (DMF), ethyl acetate (EtOAc) and petroleum (60–90${}^{\circ }C$) were purchased from Sigma-Aldrich (Beijing, China). All solvents are dried by calcium hydride or molecular sieve. Oleanolic acid (OA), methyl iodide (MeI), benzyl bromide (BnBr), sodium hydride (NaH), Lithium Aluminum Hydride (LAH), trifluoromethanesulfonic anhydride (Tf${}_{2}$O), sodium azide (NaN${}_{3}$), anhydrous potassium carbonate (K${}_{2}$CO${}_{3}$), copper sulfate pentahydrate (CuSO${}_{4}$·5H${}_{2}$O), sodium ascorbate and substituted phenylacetylene were purchased from Sinopharm Chemical Reagent Beijing Co., Ltd. (Beijing, China). All reagents are not further purified. All reactions were carried out under a nitrogen atmosphere where necessary. All reactions were monitored by thin-layer chromatography (TLC) (silica gel thin plate purchased from Yantai Dexin Biological Technology Co., Ltd., Yantai, China) analysis and TLC was performed on silica gel HF with detection by charring with 30% (*v/v*) H${}_{2}$SO${}_{4}$ in CH${}_{3}$OH or by UV detection (254 nm). Column chromatography was conducted by elution of a column (8 × 100, 16 × 240, 18 × 300, 35 × 400 mm) of silica gel (200–300 mesh) with EtOAc–PE (b. p. 60–90 ${}^{\circ }C$) as the eluent. ${}^{1}$H NMR (400 MHz) and ${}^{13}$C NMR (100 MHz) spectra were recorded in CDCl${}_{3}$ with a Bruker DPX400 spectrometer (Brook (Beijing) Science and Technology Co., Ltd., Beijing, China), using Tetramethyl silane (TMS) as internal standard. ${}^{1}$H NMR Spectroscopy splitting patterns were designated as singlet (s), doublet (d), triplet (t), and quartet (q). Splitting patterns that could not be interpreted or easily visualized were designated as multiplet (m) or broad (br). Mass spectra were obtained with Agilent 1100 series LC/MSD mass spectrometer (Agilent Technologies Inc., Beijing, China). High-resolution mass spectra (HRMS) were performed by researchers at Peking University. Melting points were measured on a Yanagimoto melting-point apparatus (Yanagimoto MFG CO, Kyoto, Japan) and are uncorrected. Solutions were concentrated at a temperature <60 ${}^{\circ }C$ under diminished pressure.

### 3.2. Chemical Synthesis

*General procedure for the synthesis of compounds **3** and **4***. Slight modifications of the synthesis methods referring to the literature [26,27,28] have been made. We use a one-pot method to synthesize **3** and **4** instead of the step-by-step method. The yield of the intermediate product **3** or **4** prepared by this method is desirably more than 90%. This method is innovative and reduces the cumbersome post-processing steps.

*General procedure for the synthesis of compounds **5** and **6***. Under the protection of nitrogen, place the reaction device in a low-temperature tank, add dry pyridine (20 mmol, 1.6 mL) to a three-neck round bottom flask, and add trifluoromethanesulfonic anhydride (7.5 mmol, 1.3 mL). The raw materials **3** and **4** (5 mmol) dissolved in anhydrous CH${}_{2}$Cl${}_{2}$ in a constant-pressure dropping funnel were slowly released. Reaction at low temperature lasted 1 h. The reaction was monitored by thin-layer chromatography (TLC) [V (ethyl acetate): V (petroleum ether) = 1:12]. After the reaction had completed, dichloromethane was added for extraction (3 × 50 mL), washed with water, dried, and concentrated. The product was dissolved in anhydrous DMF, sodium azide (20 mmol, 1.49 g) was added, and the reaction was carried out at 60 ${}^{\circ }C$ for 4 h. The reaction was monitored by thin-layer chromatography (TLC) [V (ethyl acetate): V (petroleum ether) = 1: 12] until the reaction was complete, and extracted with dichloromethane (3 × 50 mL), washed with water, dried and concentrated. Purification by silica gel chromatography with 20:1 petroleum ether–EtOAc as the eluent afforded **5** or **6**.

*General procedure for the synthesis of title compounds **1** and **2***. Intermediate **5** or **6** (1 mmol) was placed in a 250 mL round-bottom flask, a mixture of 10 mL of methanol and 10 mL of water was added, then catalytic amount of CuSO${}_{4}$·5H${}_{2}$O and sodium ascorbate were added, and finally substituted phenylacetylene or alkyl alkyne (1.2 mmol) was added. The reaction was monitored by thin-layer chromatography (TLC) [V (ethyl acetate): V (petroleum ether) = 1:15] and the reaction was stopped after 10 h. After being filtered, extracted with CH${}_{2}$Cl${}_{2}$ (3 × 50 mL), dried (Na${}_{2}$SO${}_{4}$ anhydrous), and taking [V (ethyl acetate): V (petroleum ether) = 1:30] as an eluent, the target compound **1a∼1i**/**2a∼2i** was obtained by silica gel column chromatography.

**((6a*****S*****,6b*****R*****,12a*****R*****)-10-methoxy-2,2,6a,6b,9,9,12a-heptamethyl-1,3,4,5,6,6a,6b,7,8,8a,9,10, 11,12,12a,12b,13,14b-octadecahydropicen-4a(2*****H*****)-yl)methanol (3)** [29]. Yield: 92%. White foamy solid, m.p. 293.0∼293.5 ${}^{\circ }C$. ${}^{1}$H NMR (400 MHz, CDCl${}_{3}$): δ 5.19 (t, *J* = 3.2 Hz, 1H), 3.54 (d, *J* = 10.9 Hz, 1H), 3.35 (s, 3H), 3.20 (d, *J* = 10.9 Hz, 1H), 2.67 (dd, *J* = 11.7, 4.3 Hz, 1H), 2.00–1.94 (m, 1H), 1.90–1.84 (m, 3H), 1.83–1.74 (m, 2H), 1.74–1.67 (m, 2H), 1.64 (d, J = 3.3 Hz, 1H), 1.57–1.46 (m, 5H), 1.45–1.35 (m, 3H), 1.32 (d, *J* = 10.8 Hz, 3H), 1.19 (s, 1H), 1.16 (s, 3H), 1.10–1.03 (m, 1H), 0.98 (s, 3H), 0.93 (d, *J* = 2.5 Hz, 6H), 0.88 (d, *J* = 5.3 Hz, 6H), 0.77 (s, 3H), 0.72 (d, *J* = 11.2 Hz, 1H).

**((6a*****S*****,6b*****R*****,12a*****R*****)-10-(benzyloxy)-2,2,6a,6b,9,9,12a-heptamethyl-1,3,4,5,6,6a,6b,7,8,8a, 9,10,11,12,12a,12b,13,14b-octadecahydropicen-4a(2*****H*****)-yl)methanol (4)** [30]. Yield 90%. White foamy solid, m.p. 228.0∼228.5 ${}^{\circ }C$. ${}^{1}$H NMR (400 MHz, CDCl${}_{3}$): δ 7.37–7.23 (m, 5H), 5.19 (s, 1H), 4.66 (d, *J* = 11.9 Hz, 1H), 4.42 (d, *J* = 11.9 Hz, 1H), 3.54 (d, *J* = 10.8 Hz, 1H), 3.19 (d, *J* = 10.9 Hz, 1H), 2.93 (dd, *J* = 11.6, 4.3 Hz, 1H), 1.97 (dd, *J* = 13.4, 3.7 Hz, 1H), 1.88 (dd, *J* = 13.4, 9.4 Hz, 3H), 1.82–1.74 (m, 2H), 1.74–1.68 (m, 2H), 1.65 (d, *J* = 13.1 Hz, 1H), 1.52 (dt, *J* = 14.3, 7.8 Hz, 5H), 1.47–1.39 (m, 2H), 1.31 (q, *J* = 13.5 Hz, 4H), 1.18 (s, 1H), 1.15 (s, 3H), 1.06 (d, *J* = 12.1 Hz, 1H), 1.00 (s, 3H), 0.96–0.93 (m, 6H), 0.90–0.85 (m, 9H), 0.73 (d, *J* = 11.1 Hz, 1H).

**(6a*****S*****,6b*****R*****,12a*****R*****)-4a-(azidomethyl)-10-methoxy-2,2,6a,6b,9,9,12a-heptamethyl-1,2,3,4,4a, 5,6,6a,6b,7,8,8a,9,10,11,12,12a,12b,13,14b-icosahydropicene (5)**. Yield: 87%. White foamy solid, m.p. 175.5∼177.2 ${}^{\circ }C$. ${}^{1}$H NMR (400 MHz, CDCl${}_{3}$): δ 5.24 (s, 1H), 3.36 (s, 3H), 3.30 (d, *J* = 12.1 Hz, 1H), 3.02 (d, *J* = 12.1 Hz, 1H), 2.67 (dd, *J* = 11.5, 3.8 Hz, 1H), 2.06–1.99 (m, 1H), 1.96–1.85 (m, 3H), 1.81–1.74 (m, 1H), 1.73–1.62 (m, 3H), 1.58–1.51 (m, 3H), 1.48–1.40 (m, 2H), 1.32 (dd, *J* = 22.0, 11.9 Hz, 5H), 1.26 (s, 3H), 1.19 (s, 1H), 1.15 (s, 3H), 1.12–1.01 (m, 3H), 0.98 (s, 3H), 0.95 (d, *J* = 6.1 Hz, 6H), 0.88 (s, 9H), 0.77 (s, 3H), 0.73 (d, *J* = 11.4 Hz, 1H); ${}^{13}$C NMR (100 MHz, CDCl${}_{3}$): δ 143.67 (C-13), 123.34 (C-12), 88.81, 60.59, 57.69, 55.91, 47.72, 46.54, 43.67, 40.05, 38.89, 38.71, 37.49, 37.11, 34.25, 33.26, 32.68, 32.14, 31.04, 28.28, 26.19, 25.68, 23.77, 23.69, 22.97, 22.21, 18.38, 17.01, 16.49, 15.64, 14.25.

**(6a*****S*****,6b*****R*****,12a*****R*****)-4a-(azidomethyl)-10-(benzyloxy)-2,2,6a,6b,9,9,12a-heptamethyl-1,2, 3,4,4a,5,6,6a,6b,7,8,8a,9,10,11,12,12a,12b,13,14b-icosahydropicene (6)**. Yield: 89%. White foamy solid, m.p. 158.5∼160.1 ${}^{\circ }C$. ${}^{1}$H NMR (400 MHz, CDCl${}_{3}$): δ 7.37–7.30 (m, 4H), 7.28–7.24 (m, 1H), 5.23 (s, 1H), 4.66 (d, *J* = 11.9 Hz, 1H), 4.42 (d, *J* = 11.9 Hz, 1H), 3.30 (d, *J* = 12.1 Hz, 1H), 3.02 (d, *J* = 12.1 Hz, 1H), 2.93 (dd, *J* = 11.6, 4.2 Hz, 1H), 2.05–1.99 (m, 1H), 1.95–1.85 (m, 3H), 1.82–1.76 (m, 1H), 1.69 (dd, *J* = 27.9, 14.4 Hz, 3H), 1.53 (dt, *J* = 11.9, 5.5 Hz, 4H), 1.47 (d, *J* = 4.5 Hz, 1H), 1.45–1.38 (m, 1H), 1.32 (m, 4H), 1.26 (s, 2H), 1.19 (s, 1H), 1.15 (s, 3H), 1.10 (s, 1H), 1.00 (s, 3H), 0.95 (s, 6H), 0.88 (d, *J* = 2.7 Hz, 6H), 0.85 (s, 3H), 0.73 (d, *J* = 10.8 Hz, 1H); ${}^{13}$C NMR(100 MHz, CDCl${}_{3}$): δ 143.65 (C-13), 139.66, 128.31, 127.55, 127.32 (aromatic carbons), 123.32 (C-12), 86.64, 77.48, 77.16, 76.84, 71.51, 60.57, 55.84, 47.69, 46.52, 43.65, 41.75, 40.04, 39.08, 38.71, 37.47, 37.05, 33.26, 32.66, 32.13, 31.03, 28.45, 26.19, 25.66, 23.76, 23.69, 22.95, 22.92, 18.42, 17.00, 16.78, 15.67.

**1-(((4a*****S*****,6a*****S*****,6b*****R*****,12a*****R*****)-10-methoxy-2,2,6a,6b,9,9,12a-heptamethyl-1,3,4,5,6,6a,6b,7,8, 8a,9,10,11,12,12a,12b,13,14b-octadecahydropicen-4a(2*****H*****)-yl)methyl)-4-phenyl-1*****H*****-1,2,3- triazole (1a)**. Yield: 82%. White foamy solid, m.p. 268.4∼270.5 ${}^{\circ }C$. ${}^{1}$H NMR (400 MHz, CDCl${}_{3}$): δ 8.29 (d, *J* = 8.8 Hz, 2H), 8.01 (d, *J* = 8.8 Hz, 2H), 7.83 (s, 1H), 7.27 (s, 1H), 5.37 (s, 1H), 4.60 (d, *J* = 14.0 Hz, 1H), 3.97 (d, *J* = 14.1 Hz, 1H), 3.37 (s, 3H), 2.69 (dd, *J* = 11.6, 4.2 Hz, 1H), 2.13 (dd, *J* = 15.7, 8.3 Hz, 2H), 2.04 (d, *J* = 3.9 Hz, 1H), 2.01–1.94 (m, 2H), 1.85–1.75 (m, 2H), 1.69 (d, *J* = 13.0 Hz, 1H), 1.64–1.54 (m, 4H), 1.47–1.40 (m, 3H), 1.33 (s, 1H), 1.28 (d, *J* = 3.2 Hz, 2H), 1.26 (s, 3H), 1.22 (s, 3H), 1.19–1.15 (m, 2H), 1.12 (s, 3H), 1.00 (s, 3H), 0.98 (s, 3H), 0.90 (s, 3H), 0.83 (s, 3H), 0.80 (s, 3H); ${}^{13}$C NMR (100 MHz, CDCl${}_{3}$): δ 147.09, 143.46 (C-13), 130.90, 128.88, 128.08, 125.80, 120.99 (aromatic carbons), 123.86 (C-12), 88.74, 58.35, 57.63, 55.84, 47.69, 46.45, 44.21, 41.69, 40.17, 38.85, 38.66, 37.26, 37.07, 34.02, 33.13, 32.52, 32.09, 30.84, 29.78, 28.24, 26.46, 25.67, 23.83, 23.54, 22.69, 22.16, 18.32, 17.08, 16.45, 15.61 (8×CH${}_{3}$); HRMS calculated for C${}_{39}$H${}_{58}$ON${}_{3}$ [M + H]${}^{+}$ 584.4580, found 584.4582.

**3-(1-(((4a*****S*****,6a*****S*****,6b*****R*****,12a*****R*****)-10-methoxy-2,2,6a,6b,9,9,12a-heptamethyl-1,3,4,5,6,6a,6b, 7,8,8a,9,10,11,12,12a,12b,13,14b-octadecahydropicen-4a(2*****H*****)-yl)methyl)-1*****H*****-1,2,3-triazol- 4-yl)an iline (1b)**. Yield: 84%. White foamy solid, m.p. 239.4–240.8 ${}^{\circ }C$. ${}^{1}$H NMR (400 MHz, CDCl${}_{3}$): δ 7.81 (dd, *J* = 8.6, 5.4 Hz, 2H), 7.65 (s, 1H), 7.11 (t, *J* = 8.6 Hz, 2H), 5.35 (t, *J* = 3.6 Hz, 1H), 4.54 (d, *J* = 14.0 Hz, 1H), 4.30 (t, *J* = 6.7 Hz, 1H), 4.09 (d, *J* = 6.7 Hz, 1H), 3.92 (d, *J* = 14.0 Hz, 1H), 3.37 (s, 3H), 2.69 (dd, *J* = 11.6, 4.1 Hz, 1H), 2.14 (dd, *J* = 15.6, 6.6 Hz, 2H), 2.03 (dd, *J* = 14.0, 5.2 Hz, 1H), 1.99–1.92 (m, 2H), 1.79 (t, *J* = 13.5 Hz, 3H), 1.68 (d, *J* = 12.9 Hz, 1H), 1.58 (m, 5H), 1.43 (dt, *J* = 13.9, 8.8 Hz, 3H), 1.27 (d, *J* = 8.9 Hz, 3H), 1.21 (s, 3H), 1.15 (s, 2H), 1.10 (s, 3H), 1.00 (s, 3H), 0.97 (s, 3H), 0.89 (s, 3H), 0.82 (s, 3H), 0.79 (s, 3H), 0.75 (s, 1H); ${}^{13}$C NMR (100 MHz, CDCl${}_{3}$): δ 147.21, 147.03, 143.50 (C-13), 131.83, 129.80, 123.86, 116.11, 114.91, 112.37 (aromatic carbons), 121.03 (C-12), 88.76, 58.31, 57.65, 55.87, 47.71, 44.20, 41.71, 40.18, 38.87, 38.68, 37.27, 37.09, 34.04, 33.13, 32.55, 32.06, 30.85, 28.25, 26.46, 25.69, 23.85, 23.55, 22.18, 18.34, 17.10, 16.46, 15.62 (8×CH${}_{3}$); HRMS calculated for C${}_{39}$H${}_{59}$ON${}_{4}$ [M + H]${}^{+}$ 599.4689, found 599.4689.

**1-(((4a*****S*****,6a*****S*****,6b*****R*****,12a*****R*****)-10-methoxy-2,2,6a,6b,9,9,12a-heptamethyl-1,3,4,5,6,6a,6b,7,8,8a, 9,10,11,12,12a,12b,13,14b-octadecahydropicen-4a(2*****H*****)-yl)methyl)-4-(4-methoxyphen yl)-1*****H*****- 1,2,3-Triazole (1c)**. Yield: 86%. White foamy solid, m.p. 114.4–114.6 ${}^{\circ }C$. ${}^{1}$H NMR (400 MHz, CDCl${}_{3}$): δ 7.76 (d, *J* = 8.8 Hz, 2H), 7.60 (s, 1H), 6.95 (d, *J* = 8.8 Hz, 2H), 5.34 (t, *J* = 3.5 Hz, 1H), 4.51 (d, *J* = 14.0 Hz, 1H), 3.90 (d, *J* = 14.0 Hz, 1H), 3.83 (s, 3H), 3.36 (s, 3H), 2.68 (dd, *J* = 11.6, 4.1 Hz, 1H), 2.13 (dd, *J* = 17.6, 7.1 Hz, 2H), 2.05–1.97 (m, 2H), 1.96–1.85 (m, 2H), 1.78 (t, *J* = 13.5 Hz, 2H), 1.68 (d, *J* = 13.1 Hz, 1H), 1.58 (dt, *J* = 11.4, 6.6 Hz, 4H), 1.42 (s, 3H), 1.33–1.23 (m, 2H), 1.21 (s, 3H), 1.19–1.14 (m, 2H), 1.10 (s, 3H), 1.06 (d, *J* = 8.4 Hz, 1H), 1.00 (s, 3H), 0.97 (s, 3H), 0.93–0.90 (m, 1H), 0.88 (s, 3H), 0.81 (s, 3H), 0.79 (s, 3H), 0.76 (d, *J* = 11.5 Hz, 1H); ${}^{13}$C NMR (100 MHz, CDCl${}_{3}$): δ 159.61, 146.91, 143.49 (C-13), 127.06, 123.80, 123.69 (aromatic carbons), 120.17 (C-12), 114.32, 88.70, 58.29, 57.6, 55.84, 55.38, 47.69, 44.23, 41.68, 40.16, 38.84, 38.65, 37.24, 37.06, 33.12, 32.10, 30.82, 28.23, 27.00, 26.44, 25.67, 23.82, 23.54, 22.15, 8.32, 17.06, 16.44, 15.59 (9×CH${}_{3}$); HRMS calculated for C${}_{40}$H${}_{60}$O${}_{2}$N${}_{3}$ [M + H]${}^{+}$ 614.4686, found 614.4688.

**1-(((4a*****S*****,6a*****S*****,6b*****R*****,12a*****R*****)-10-methoxy-2,2,6a,6b,9,9,12a-heptamethyl-1,3,4,5,6,6a,6b,7,8, 8a,9,10,11,12,12a,12b,13,14b-octadecahydropicen-4a(2*****H*****)-yl)methyl)-4-(m-tolyl)-1*****H*****-1,2,3 -triazole (1d)**. Yield: 84%. White foamy solid, m.p. 230.1–230.6 ${}^{\circ }C$. ${}^{1}$H NMR (400 MHz, CDCl${}_{3}$): δ 7.68 (d, *J* = 8.1 Hz, 2H), 7.62 (d, *J* = 7.6 Hz, 1H), 7.30 (t, *J* = 7.6 Hz, 1H), 7.13 (d, *J* = 7.5 Hz, 1H), 5.35 (s, 1H), 4.53 (d, *J* = 14.0 Hz, 1H), 3.92 (d, *J* = 14.0 Hz, 1H), 3.37 (s, 3H), 2.68 (d, *J* = 7.6 Hz, 1H), 2.40 (s, 3H), 2.14 (dd, *J* = 16.0, 6.6 Hz, 2H), 2.06–1.92 (m, 3H), 1.78 (t, *J* = 13.4 Hz, 2H), 1.68 (d, *J* = 13.0 Hz, 1H), 1.63–1.52 (m, 4H), 1.42 (t, *J* = 9.4 Hz, 4H), 1.30–1.25 (m, 1H), 1.21 (s, 3H), 1.19–1.13 (m, 3H), 1.11 (s, 3H), 1.08 (s, 1H), 0.98 (d, *J* = 11.6 Hz, 6H), 0.92 (s, 1H), 0.88 (s, 3H), 0.80 (d, *J* = 6.6 Hz, 6H), 0.76 (d, *J* = 11.8 Hz, 1H); ${}^{13}$C NMR (100 MHz, CDCl${}_{3}$): δ 147.12, 143.43 (C-13), 138.45, 130.79, 128.78, 128.73, 126.42, 123.79, 120.89 (aromatic carbons), 122.86 (C-12), 88.66, 58.28, 57.57, 55.81, 47.66, 46.43, 44.20, 41.65, 40.13, 38.81, 38.62, 37.21, 37.03, 33.10, 32.07, 30.79, 28.22, 26.42, 25.64, 23.79, 23.52, 22.12, 21.50, 18.29, 17.04, 16.43, 15.58 (9×CH${}_{3}$); HRMS calculated for C${}_{40}$H${}_{60}$ON${}_{3}$ [M + H]${}^{+}$ 598.4736, found 598.4739.

**4-(4-chlorophenyl)-1-(((4a*****S*****,6a*****S*****,6b*****R*****,12a*****R*****)-10-methoxy-2,2,6a,6b,9,9,12a-heptamethyl -1,3,4,5,6,6a,6b,7,8,8a,9,10,11,12,12a,12b,13,14b-octadecahydropicen-4a(2*****H*****)-yl)methyl)-1*****H*****- 1,2,3-Triazole (1e)**. Yield: 79%. White foamy solid, m.p. 99.5–105.20 ${}^{\circ }C$. ${}^{1}$H NMR (400 MHz, CDCl${}_{3}$): δ 7.84 (d, *J* = 7.4 Hz, 2H), 7.69 (s, 1H), 7.42 (t, *J* = 7.5 Hz, 2H), 5.35 (s, 1H), 4.54 (d, *J* = 14.0 Hz, 1H), 3.93 (d, *J* = 14.0 Hz, 1H), 3.37 (s, 3H), 2.69 (dd, *J* = 11.6, 4.0 Hz, 1H), 2.21–2.08 (m, 3H), 2.06–1.94 (m, 3H), 1.78 (t, *J* = 13.3 Hz, 2H), 1.68 (d, *J* = 12.9 Hz, 1H), 1.57 (dt, *J* = 20.7, 12.2 Hz, 4H), 1.42 (t, *J* = 9.4 Hz, 3H), 1.35 (d, *J* = 11.9 Hz, 1H), 1.27 (d, *J* = 11.4 Hz, 3H), 1.21 (s, 3H), 1.16 (s, 1H), 1.11 (s, 3H), 0.99 (d, *J* = 11.9 Hz, 6H), 0.92 (s, 1H), 0.88 (s, 3H), 0.80 (d, *J* = 6.7 Hz, 6H), 0.75 (s, 1H); ${}^{13}$C NMR (100 MHz, CDCl${}_{3}$): δ 143.35 (C-13), 133.74, 129.45, 129.03, 126.99, 123.86 (aromatic carbons), 121.03 (C-12), 88.65, 58.39, 57.58, 55.81, 47.65, 46.39, 44.24, 41.65, 40.13, 38.81, 38.62, 37.23, 37.03, 33.98, 33.09, 32.10, 30.79, 28.22, 26.42, 25.63, 23.80, 23.51, 22.12, 18.29, 17.04, 16.43, 15.58 (8×CH${}_{3}$); HRMS calculated for C${}_{39}$H${}_{57}$ClON${}_{3}$ [M + H]${}^{+}$ 618.4190, found 618.4189.

**1-(((4a*****S*****,6a*****S*****,6b*****R*****,12a*****R*****)-10-methoxy-2,2,6a,6b,9,9,12a-heptamethyl-1,3,4,5,6,6a,6b,7,8, 8a,9,10,11,12,12a,12b,13,14b-octadecahydropicen-4a(2*****H*****)-yl)methyl)-4-(4-nitrophenyl)-1*****H*****-1,2,3-Triazole (1f)**. Yield: 83%. White foamy solid, m.p. 253.2–256.7 ${}^{\circ }C$. ${}^{1}$H NMR (400 MHz, CDCl${}_{3}$): δ 7.77 (d, *J* = 8.4 Hz, 2H), 7.68 (s, 1H), 7.37 (d, *J* = 8.4 Hz, 2H), 5.34 (s, 1H), 4.54 (d, *J* = 14.0 Hz, 1H), 3.90 (d, *J* = 14.0 Hz, 1H), 3.36 (s, 3H), 2.68 (dd, *J* = 11.6, 4.0 Hz, 1H), 2.13 (dd, *J* = 17.6, 6.9 Hz, 2H), 2.01 (dd, *J* = 13.6, 3.7 Hz, 1H), 1.98–1.92 (m, 2H), 1.78 (t, *J* = 13.4 Hz, 3H), 1.68 (d, *J* = 13.0 Hz, 1H), 1.57 (dt, *J* = 20.1, 11.4 Hz, 5H), 1.45–1.37 (m, 3H), 1.25 (s, 3H), 1.21 (s, 3H), 1.16 (d, *J* = 9.7 Hz, 2H), 1.10 (s, 3H), 0.98 (d, *J* = 13.5 Hz, 6H), 0.88 (s, 3H), 0.80 (d, *J* = 9.1 Hz, 6H), 0.74 (s, 1H); ${}^{13}$C NMR (100 MHz, CDCl${}_{3}$): δ 147.43, 145.05, 143.33(C-13), 137.21, 126.26, 124.44, 124.11(aromatic carbons), 122.47 (C-12), 88.78, 58.70, 57.70, 55.89, 47.72, 46.44, 44.35, 41.76, 40.24, 38.91, 38.71, 37.39, 37.13, 34.03, 33.13 32.21, 30.89, 28.28, 26.51, 23.89, 23.57, 22.20, 18.36, 17.15, 16.49, 15.66 (8×CH${}_{3}$); HRMS calculated for C${}_{39}$H${}_{57}$O${}_{3}$N${}_{4}$ [M + H]${}^{+}$ 629.4431, found 629.4432.

**4-(4-fluorophenyl)-1-(((4a*****S*****,6a*****S*****,6b*****R*****,12a*****R*****)-10-methoxy-2,2,6a,6b,9,9,12a-heptamethyl- 1,3,4,5,6,6a,6b,7,8,8a,9,10,11,12,12a,12b,13,14b-octadecahydropicen-4a(2*****H*****)-yl)methyl)-1*****H*****- 1,2,3-Triazole (1g)**. Yield: 81%. White foamy solid, m.p. 232.7–236.2 ${}^{\circ }C$. ${}^{1}$H NMR (400 MHz, CDCl${}_{3}$): δ 7.64 (s, 1H), 7.26 (d, *J* = 4.8 Hz, 1H), 7.17 (dt, *J* = 13.2, 7.4 Hz, 2H), 6.65 (d, *J* = 7.2 Hz, 1H), 5.35 (s, 1H), 4.51 (d, *J* = 14.0 Hz, 1H), 3.93 (d, *J* = 14.0 Hz, 1H), 3.37 (s, 4H), 2.69 (dd, *J* = 11.6, 4.0 Hz, 1H), 2.12 (d, *J* = 13.5 Hz, 2H), 2.05–1.92 (m, 3H), 1.80 (d, *J* = 13.6 Hz, 2H), 1.76–1.64 (m, 2H), 1.62–1.52 (m, 4H), 1.47–1.38 (m, 3H), 1.26 (s, 2H), 1.21 (s, 3H), 1.16 (d, *J* = 11.0 Hz, 3H), 1.10 (s, 3H), 0.98 (d, *J* = 12.0 Hz, 6H), 0.91 (s, 1H), 0.88 (s, 3H), 0.80 (d, *J* = 3.0 Hz, 6H), 0.75 (s, 1H); ${}^{13}$C NMR (100 MHz, CDCl${}_{3}$): δ 163.92, 161.47, 146.25, 143.45 (C-13), 130.98, 128.94, 127.55, 127.19, 120.72, 115.96, 115.74 (aromatic carbons), 123.90 (C-12), 88.73, 58.41, 57.64, 55.86, 47.70, 44.27, 41.71, 40.19, 38.86, 38.68, 37.28, 37.08, 33.13, 32.14, 30.85, 28.25, 26.47, 25.69, 23.85, 23.55, 22.17, 18.33, 17.09, 16.46, 15.62 (8×CH${}_{3}$); HRMS calculated for C${}_{39}$H${}_{56}$ON${}_{3}$F [M + H]${}^{+}$ 602.4486, found 602.4487.

**1-(1-(((4a*****S*****,6a*****S*****,6b*****R*****,12a*****R*****)-10-methoxy-2,2,6a,6b,9,9,12a-heptamethyl-1,3,4,5,6,6a,6b, 7,8,8a,9,10,11,12,12a,12b,13,14b-octadecahydropicen-4a(2*****H*****)-yl)methyl)-1*****H*****-1,2,3-triazol- 4-yl) ethan-1-ol (1h)**. Yield: 79%. White foamy solid, m.p. 104.1-108.7 ${}^{\circ }C$. ${}^{1}$H NMR (400 MHz, CDCl${}_{3}$): δ 7.43 (s, 1H), 5.33 (s, 1H), 5.07 (s, 1H), 4.63–4.36 (m, 1H), 3.98–3.77 (m, 1H), 3.36 (s, 3H), 2.69 (d, *J* = 11.1 Hz, 1H), 2.21–2.01 (m, 3H), 1.93 (s, 3H), 1.83–1.63 (m, 3H), 1.58 (s, 3H), 1.57 (s, 3H), 1.46 (d, *J* = 38.9 Hz, 5H), 1.25 (s, 1H), 1.19 (s, 3H), 1.12 (d, *J* = 18.1 Hz, 4H), 1.06 (s, 3H), 0.97 (d, *J* = 14.7 Hz, 6H), 0.88 (s, 3H), 0.79 (s, 6H), 0.74 (s, 1H); ${}^{13}$C NMR (100 MHz, CDCl${}_{3}$): δ 151.80, 143.32 (C-13), 123.77, 121.50 (C-12), 88.70, 62.92, 58.20, 57.57, 55.78, 47.61, 46.4, 44.21, 44.15, 41.62, 40.08, 38.79, 38.6, 37.14, 37.00, 33.93, 33.06, 32.46, 30.76, 28.18, 26.37, 25.59, 23.74, 23.44, 23.41, 23.28, 22.10, 18.26, 16.99, 16.39, 15.53 (9×CH${}_{3}$); HRMS calculated for C${}_{35}$H${}_{58}$O${}_{2}$N${}_{3}$ [M + H]${}^{+}$ 552.4529, found 552.4528.

**2-(1-(((4a*****S*****,6a*****S*****,6b*****R*****,12a*****R*****)-10-methoxy-2,2,6a,6b,9,9,12a-heptamethyl-1,3,4,5,6,6a,6b, 7,8,8a,9,10,11,12,12a,12b,13,14b-octadecahydropicen-4a(2*****H*****)-yl)methyl)-1*****H*****-1,2,3-triazol- 4-yl)propan-2-ol (1i)**. Yield: 77%. Oily. ${}^{1}$H NMR (400 MHz, CDCl${}_{3}$): δ 7.39 (s, 1H), 5.33 (s, 1H), 4.47 (d, *J* = 14.0 Hz, 1H), 3.87 (d, *J* = 14.0 Hz, 1H), 3.36 (s, 3H), 2.69 (dd, *J* = 11.7, 4.3 Hz, 1H), 2.12–2.05 (m, 2H), 2.05–2.00 (m, 1H), 2.00–1.89 (m, 3H), 1.82–1.76 (m, 2H), 1.71 (d, *J* = 16.0 Hz, 1H), 1.64 (s, 6H), 1.59–1.54 (m, 3H), 1.52–1.49 (m, 1H), 1.46–1.35 (m, 4H), 1.26 (s, 2H), 1.20 (s, 3H), 1.14 (dd, *J* = 11.2, 5.4 Hz, 2H), 1.07 (s, 3H), 0.98 (d, *J* = 14.5 Hz, 6H), 0.91 (s, 1H), 0.88 (s, 3H), 0.79 (d, *J* = 4.2 Hz, 6H), 0.74 (s, 1H).; ${}^{13}$C NMR (100 MHz, CDCl${}_{3}$): δ 155.03, 143.37 (C-13), 123.80 (C-12), 120.54, 88.73, 71.88, 58.23, 57.60, 55.81, 47.65, 44.17, 41.64, 40.12, 38.82, 38.63, 37.17, 37.03, 33.09, 31.94, 30.80, 30.56, 28.21, 26.40, 25.61, 23.77, 23.42, 22.13, 18.29, 17.01, 16.42, 15.57 (10×CH${}_{3}$); HRMS calculated for C${}_{36}$H${}_{60}$O${}_{2}$N${}_{3}$ [M + H]${}^{+}$ 566.4686, found 556.4689.

**1-(((4a*****S*****,6a*****S*****,6b*****R*****,12a*****R*****)-10-(benzyloxy)-2,2,6a,6b,9,9,12a-heptamethyl-1,3,4,5,6,6a,6b, 7,8,8a,9,10,11,12,12a,12b,13,14b-octadecahydropicen-4a(2*****H*****)-yl)methyl)-4-phenyl-1*****H*****-1,2, 3-triazole (2a)**. Yield: 82%. White foamy solid, m.p. 252.6–255.9 ${}^{\circ }C$. ${}^{1}$H NMR (400 MHz, CDCl${}_{3}$): δ 7.84 (d, *J* = 7.3 Hz, 2H), 7.68 (s, 1H), 7.42 (t, *J* = 7.6 Hz, 2H), 7.36 (t, *J* = 5.4 Hz, 3H), 7.33 (s, 1H), 7.32–7.29 (m, 1H), 7.28–7.24 (m, 1H), 5.35 (t, *J* = 3.5 Hz, 1H), 4.67 (d, *J* = 11.9 Hz, 1H), 4.54 (d, *J* = 14.0 Hz, 1H), 4.44 (d, *J* = 11.9 Hz, 1H), 3.93 (d, *J* = 14.0 Hz, 1H), 2.95 (dd, *J* = 11.6, 4.2 Hz, 1H), 2.17–2.08 (m, 2H), 2.05–1.92 (m, 3H), 1.80 (d, *J* = 13.5 Hz, 2H), 1.76–1.65 (m, 2H), 1.58 (t, *J* = 14.5 Hz, 5H), 1.42 (s, 2H), 1.32–1.23 (m, 2H), 1.20 (s, 3H), 1.16 (s, 2H), 1.11 (s, 3H), 1.00 (d, *J* = 14.5 Hz, 6H), 0.96 (d, *J* = 7.9 Hz, 2H), 0.88 (d, *J* = 4.8 Hz, 6H), 0.81 (s, 3H), 0.77 (d, *J* = 11.6 Hz, 1H); ${}^{13}$C NMR (100 MHz, CDCl${}_{3}$): δ 147.12, 143.51 (C-13), 139.65, 130.98, 128.91, 128.30, 128.10, 127.54, 127.31, 125.82, 123.88(aromatic carbons), 120.97 (C-12), 86.62, 71.51, 58.39, 55.84, 47.72, 44.25, 41.74, 40.22, 39.09, 37.30, 33.16, 30.87, 28.45, 26.50, 23.87, 23.58, 18.41, 17.12, 16.77, 15.68 (7×CH${}_{3}$); HRMS calculated for C${}_{45}$H${}_{62}$ON${}_{3}$ [M + H]${}^{+}$ 660.4893, found 660.4891.

**3-(1-(((4a*****S*****,6a*****S*****,6b*****R*****,12a*****R*****)-10-(benzyloxy)-2,2,6a,6b,9,9,12a-heptamethyl-1,3,4,5,6,6a, 6b,7,8,8a,9,10,11,12,12a,12b,13,14b-octadecahydropicen-4a(2*****H*****)-yl)methyl)-1*****H*****-1,2,3-triazol- 4-yl)aniline (2b)**. Yield: 84%. White foamy solid, m.p. 112.8–113.0 ${}^{\circ }C$. ${}^{1}$H NMR (400 MHz, CDCl${}_{3}$): δ 7.63 (s, 1H), 7.34 (q, *J* = 7.1 Hz, 4H), 7.26 (d, *J* = 7.0 Hz, 2H), 7.17 (d, *J* = 10.0 Hz, 2H), 6.63 (d, *J* = 6.2 Hz, 1H), 5.34 (s, 1H), 4.67 (d, *J* = 11.8 Hz, 1H), 4.50 (d, *J* = 14.0 Hz, 1H), 4.43 (d, *J* = 11.9 Hz, 1H), 3.91 (d, *J* = 14.0 Hz, 1H), 2.99–2.89 (m, 1H), 2.11 (d, *J* = 13.3 Hz, 2H), 2.04–1.90 (m, 3H), 1.85–1.72 (m, 2H), 1.69 (s, 1H), 1.64–1.47 (m, 6H), 1.43 (d, *J* = 11.4 Hz, 2H), 1.26 (s, 2H), 1.20 (s, 3H), 1.15 (d, *J* = 11.6 Hz, 2H), 1.09 (s, 3H), 1.00 (d, *J* = 16.6 Hz, 6H), 0.94 (s, 2H), 0.87 (s, 6H), 0.80 (s, 3H), 0.75 (s, 1H); ${}^{13}$C NMR (100 MHz, CDCl${}_{3}$): δ 147.12, 147.00, 143.39 (C-13), 131.70, 129.71, 128.20, 127.44, 127.22, 123.75 (aromatic carbons), 121.00 (C-12), 115.98, 114.84, 112.28(aromatic carbons), 86.53, 71.40, 58.21, 55.72, 47.61, 44.11, 41.61, 40.09, 38.98, 38.61, 37.16, 36.95, 33.07, 31.98, 30.75, 28.36, 26.39, 23.77, 23.48, 22.82, 18.31, 17.02, 16.70, 15.58 (7×CH${}_{3}$); HRMS calcd for C${}_{45}$H${}_{63}$O${}_{4}$ [M + H]${}^{+}$ 675.5002, found 675.5002.

**1-(((4a*****S*****,6a*****S*****,6b*****R*****,12a*****R*****)-10-(benzyloxy)-2,2,6a,6b,9,9,12a-heptamethyl-1,3,4,5,6,6a,6b, 7,8,8a,9,10,11,12,12a,12b,13,14b-octadecahydropicen-4a(2*****H*****)-yl)methyl)-4-(4-methoxyphenyl)- 1*****H*****-1,2,3-Triazole (2c)**. Yield: 79%. White foamy solid, m.p. 125.6–127.0 ${}^{\circ }C$. ${}^{1}$H NMR (400 MHz, CDCl${}_{3}$): δ 7.76 (d, *J* = 8.3 Hz, 2H), 7.60 (s, 1H), 7.34 (m, 4H), 7.26 (d, *J* = 7.1 Hz, 1H), 6.95 (d, *J* = 8.4 Hz, 2H), 5.34 (s, 1H), 4.67 (d, *J* = 11.9 Hz, 1H), 4.51 (d, *J* = 14.0 Hz, 1H), 4.43 (d, *J* = 11.9 Hz, 1H), 3.89 (d, *J* = 14.0 Hz, 1H), 3.82 (s, 3H), 2.94 (dd, *J* = 11.5, 3.9 Hz, 1H), 2.17–2.08 (m, 2H), 2.03–1.92 (m, 3H), 1.79 (dd, *J* = 8.4, 4.7 Hz, 2H), 1.67 (d, *J* = 12.9 Hz, 1H), 1.57 (t, *J* = 10.1 Hz, 5H), 1.45–1.38 (m, 2H), 1.24 (d, *J* = 13.1 Hz, 2H), 1.20 (s, 3H), 1.15 (s, 2H), 1.10 (s, 3H), 1.06 (s, 1H), 1.00 (d, *J* = 16.5 Hz, 6H), 0.93 (d, *J* = 14.3 Hz, 2H), 0.87 (d, *J* = 4.0 Hz, 6H), 0.81 (s, 3H), 0.77 (d, *J* = 11.1 Hz, 1H); ${}^{13}$C NMR (100 MHz, CDCl${}_{3}$): δ 159.60, 143.49 (C-13), 139.61, 128.24, 127.25, 127.05, 123.79, 120.16, 114.31 (aromatic carbons), 123.70 (C-12), 86.57, 71.45, 58.29, 55.78, 55.37, 47.67, 46.45, 44.22, 41.68, 40.16, 39.03, 38.66, 37.23, 37.01, 34.02, 33.12, 32.10, 30.82, 28.41, 26.45, 25.66, 23.82, 23.54, 22.87, 18.37, 17.06, 16.74, 15.63 (8×CH${}_{3}$); HRMS calculated for C${}_{46}$H${}_{64}$O${}_{2}$N${}_{3}$ [M + H]${}^{+}$ 690.4999, found 690.4999.

**1-(((4a*****S*****,6a*****S*****,6b*****R*****,12a*****R*****)-10-(benzyloxy)-2,2,6a,6b,9,9,12a-heptamethyl-1,3,4,5,6,6a,6b, 7,8,8a,9,10,11,12,12a,12b,13,14b-octadecahydropicen-4a(2*****H*****)-yl)methyl)-4-(m-tolyl)-1*****H*****-1, 2,3-triazole (2d)**. Yield: 78%. White foamy solid, m.p. 220.8–223.7 ${}^{\circ }C$. ${}^{1}$H NMR (400 MHz, CDCl${}_{3}$): δ 7.67 (d, *J* = 11.2 Hz, 2H), 7.61 (d, *J* = 7.6 Hz, 1H), 7.36 (t, *J* = 5.4 Hz, 3H), 7.32 (s, 1H), 7.31–7.29 (m, 1H), 7.26 (dd, *J* = 11.1, 4.1 Hz, 1H), 7.13 (d, *J* = 7.5 Hz, 1H), 5.35 (d, *J* = 3.5 Hz, 1H), 4.67 (d, *J* = 11.9 Hz, 1H), 4.53 (d, *J* = 14.0 Hz, 1H), 4.43 (d, *J* = 11.9 Hz, 1H), 3.91 (d, *J* = 14.0 Hz, 1H), 2.95 (dd, *J* = 11.6, 4.1 Hz, 1H), 2.40 (s, 3H), 2.19–2.07 (m, 2H), 2.00 (dd, *J* = 17.8, 13.5 Hz, 3H), 1.80 (dd, *J* = 8.6, 4.7 Hz, 2H), 1.68 (d, *J* = 13.0 Hz, 1H), 1.61–1.53 (m, 4H), 1.44 (d, *J* = 11.3 Hz, 2H), 1.26 (t, *J* = 12.6 Hz, 2H), 1.20 (s, 3H), 1.16 (d, *J* = 11.4 Hz, 3H), 1.11 (s, 3H), 1.07 (s, 1H), 1.02 (s, 3H), 0.93 (d, *J* = 14.2 Hz, 2H), 0.88 (d, *J* = 3.9 Hz, 6H), 0.81 (s, 3H), 0.77 (d, *J* = 11.3 Hz, 1H); ${}^{13}$C NMR (100 MHz, CDCl${}_{3}$): δ 147.18, 143.47 (C-13), 139.62, 138.52, 130.81, 128.83, 128.78, 128.26, 127.50, 127.27, 126.46, 123.83, 122.90 (aromatic carbons), 120.90 (C-12), 86.58, 71.47, 58.33, 55.80, 47.69, 41.70, 40.18, 39.05, 37.25, 37.02, 30.83, 28.42, 26.46, 23.55, 21.53, 17.08, 16.75, 15.65, 6.69, 5.94 (8×CH${}_{3}$); HRMS calculated for C${}_{46}$H${}_{64}$ON${}_{3}$ [M + H]${}^{+}$ 674.5049, found 674.5052.

**1-(((4a*****S*****,6a*****S*****,6b*****R*****,12a*****R*****)-10-(benzyloxy)-2,2,6a,6b,9,9,12a-heptamethyl-1,3,4,5,6,6a,6b, 7,8,8a,9,10,11,12,12a,12b,13,14b-octadecahydropicen-4a(2*****H*****)-yl)methyl)-4-(4-chlorophenyl)- 1*****H*****-1,2,3-Triazole (2e)**. Yield: 74%. White foamy solid, m.p. 111.0–113.3 ${}^{\circ }C$. ${}^{1}$H NMR (400 MHz, CDCl${}_{3}$): δ 7.77 (d, *J* = 8.5 Hz, 2H), 7.67 (s, 1H), 7.38 (d, *J* = 8.5 Hz, 2H), 7.35 (d, *J* = 3.6 Hz, 2H), 7.32 (d, *J* = 7.6 Hz, 2H), 7.28–7.23 (m, 1H), 5.34 (s, 1H), 4.67 (d, *J* = 11.9 Hz, 1H), 4.54 (d, *J* = 14.0 Hz, 1H), 4.44 (d, *J* = 11.9 Hz, 1H), 3.91 (d, *J* = 14.1 Hz, 1H), 2.95 (dd, *J* = 11.6, 4.2 Hz, 1H), 2.11 (d, *J* = 9.7 Hz, 2H), 2.01–1.93 (m, 2H), 1.82–1.73 (m, 2H), 1.68 (d, *J* = 13.0 Hz, 1H), 1.56 (dd, *J* = 15.4, 7.7 Hz, 5H), 1.46–1.37 (m, 2H), 1.26 (t, *J* = 12.1 Hz, 2H), 1.20 (s, 3H), 1.16 (d, *J* = 10.9 Hz, 3H), 1.10 (s, 4H), 1.07 (s, 1H), 1.00 (d, *J* = 15.8 Hz, 6H), 0.93 (d, *J* = 13.5 Hz, 2H), 0.88 (d, *J* = 6.5 Hz, 6H), 0.81 (s, 3H), 0.77 (d, *J* = 11.3 Hz, 1H).; ${}^{13}$C NMR (100 MHz, CDCl${}_{3}$): δ 146.08, 143.43 (C-13), 139.64, 133.83, 129.49, 129.11, 128.29, 127.53, 127.31, 127.06, 123.93 (aromatic carbons), 121.02 (C-12), 86.61, 71.50, 58.46, 55.82, 47.70, 46.46, 44.29, 41.73, 40.21, 39.08, 38.71, 37.30, 37.05, 34.03, 33.14, 32.16, 30.86, 28.44, 26.49, 25.70, 23.86, 23.56, 22.91, 18.40, 17.11, 16.77, 15.67 (7×CH${}_{3}$); HRMS calculated for C${}_{45}$H${}_{61}$ClON${}_{3}$ [M + H]${}^{+}$ 694.4503, found 694.4503.

**1-(((4a*****S*****,6a*****S*****,6b*****R*****,12a*****R*****)-10-(benzyloxy)-2,2,6a,6b,9,9,12a-heptamethyl-1,3,4,5,6,6a,6b, 7,8,8a,9,10,11,12,12a,12b,13,14b-octadecahydropicen-4a(2*****H*****)-yl)methyl)-4-(4-nitrophenyl)- 1*****H*****-1,2,3-Triazole (2f)**. Yield: 79%. White foamy solid, m.p. 142.1-143.9 ${}^{\circ }C$. ${}^{1}$H NMR (400 MHz, CDCl${}_{3}$): δ 8.26 (d, *J* = 8.7 Hz, 2H), 8.01 (d, *J* = 8.7 Hz, 2H), 7.86 (s, 1H), 7.41–7.30 (m, 4H), 7.29–7.19 (m, 1H), 5.36 (s, 1H), 4.67 (d, *J* = 11.9 Hz, 1H), 4.60 (d, *J* = 14.0 Hz, 1H), 4.43 (d, *J* = 11.9 Hz, 1H), 3.94 (d, *J* = 14.0 Hz, 1H), 2.95 (dd, *J* = 11.4, 3.8 Hz, 1H), 2.12 (d, *J* = 10.0 Hz, 2H), 1.99 (dd, *J* = 21.4, 7.5 Hz, 3H), 1.79 (t, *J* = 13.7 Hz, 2H), 1.68 (d, *J* = 12.9 Hz, 1H), 1.58 (m, 5H), 1.42 (t, *J* = 10.8 Hz, 2H), 1.30–1.23 (m, 2H), 1.21 (s, 3H), 1.16 (d, *J* = 14.0 Hz, 3H), 1.11 (s, 3H), 1.00 (d, *J* = 16.9 Hz, 6H), 0.94 (d, *J* = 8.6 Hz, 2H), 0.88 (d, *J* = 10.6 Hz, 6H), 0.83 (s, 3H), 0.77 (d, *J* = 11.3 Hz, 1H); ${}^{13}$C NMR (100 MHz, CDCl${}_{3}$): δ 147.27, 144.91, 143.22 (C-13), 139.57, 137.17, 128.24, 127.47, 127.26, 126.17, 124.32, 123.99 (aromatic carbons), 122.52 (C-12), 86.52, 71.44, 58.60, 55.73, 47.62, 46.34, 44.28, 41.66, 40.15, 39.01, 38.63, 37.29, 36.99, 33.95, 33.07, 32.13, 30.80, 28.39, 26.45, 25.63, 23.81, 23.50, 22.84, 18.34, 17.06, 16.73, 15.62 (7×CH${}_{3}$); HRMS calculated for C${}_{45}$H${}_{61}$O${}_{3}$N${}_{4}$ [M + H]${}^{+}$ 705.4744, found 705.4743.

**1-(((4a*****S*****,6a*****S*****,6b*****R*****,12a*****R*****)-10-(benzyloxy)-2,2,6a,6b,9,9,12a-heptamethyl-1,3,4,5,6,6a,6b, 7,8,8a,9,10,11,12,12a,12b,13,14b-octadecahydropicen-4a(2*****H*****)-yl)methyl)-4-(4-fluorophenyl)- 1*****H*****-1,2,3-Triazole (2g)**. Yield: 76%. White foamy solid, m.p. 205.2–208.5 ${}^{\circ }C$. ${}^{1}$H NMR (400 MHz, CDCl${}_{3}$): δ 7.80 (dd, *J* = 8.7, 5.4 Hz, 2H), 7.64 (s, 1H), 7.36 (t, *J* = 5.6 Hz, 3H), 7.31 (d, *J* = 7.6 Hz, 1H), 7.28–7.24 (m, 1H), 7.10 (t, *J* = 8.7 Hz, 2H), 5.34 (s, 1H), 4.67 (d, *J* = 11.9 Hz, 1H), 4.54 (d, *J* = 14.0 Hz, 1H), 4.43 (d, *J* = 11.9 Hz, 1H), 3.91 (d, *J* = 14.1 Hz, 1H), 2.95 (dd, *J* = 11.6, 4.2 Hz, 1H), 2.17–2.08 (m, 2H), 2.01–1.94 (m, 2H), 1.80 (dd, *J* = 8.7, 4.7 Hz, 2H), 1.68 (d, *J* = 13.0 Hz, 1H), 1.62–1.52 (m, 5H), 1.45–1.39 (m, 2H), 1.29 (d, *J* = 6.1 Hz, 2H), 1.25 (s, 3H), 1.20 (s, 3H), 1.16 (d, *J* = 11.1 Hz, 3H), 1.10 (s, 3H), 1.07 (s, 1H), 1.00 (d, *J* = 16.1 Hz, 6H), 0.93 (d, *J* = 13.4 Hz, 2H), 0.88 (d, *J* = 6.1 Hz, 6H), 0.81 (s, 3H), 0.77 (d, *J* = 11.4 Hz, 1H); ${}^{13}$C NMR (100 MHz, CDCl${}_{3}$): δ 163.93, 161.47, 146.25, 143.45 (C-13), 139.63, 128.28, 127.56, 127.52, 127.48, 127.30, 120.72, 115.96, 115.75 (aromatic carbons), 123.89 (C-12), 86.60, 71.49, 58.41, 55.81, 47.70, 46.45, 44.28, 41.71, 40.20, 39.07, 38.70, 37.28, 37.04, 33.14, 32.15, 30.85, 29.80, 28.43, 26.48, 25.69, 23.85, 23.55, 22.89, 18.39, 17.10, 16.76, 15.66 (7×CH${}_{3}$); HRMS calculated for C${}_{45}$H${}_{61}$ON${}_{3}$F [M+ H]${}^{+}$ 678.4799, found 678.4801.

**1-(1-(((4a*****S*****,6a*****S*****,6b*****R*****,12a*****R*****)-10-(benzyloxy)-2,2,6a,6b,9,9,12a-heptamethyl-1,3,4,5,6,6a, 6b,7,8,8a,9,10,11,12,12a,12b,13,14b-octadecahydropicen-4a(2*****H*****)-yl)methyl)-1*****H*****-1,2,3-triazol- 4-yl)ethan-1-ol (2h)**. Yield: 89%. White foamy solid, m.p. 126.8–134.6 ${}^{\circ }C$. ${}^{1}$H NMR (400 MHz, CDCl${}_{3}$): δ 7.41 (s, 1H), 7.37 (s, 1H), 7.35 (d, *J* = 3.4 Hz, 2H), 7.32 (d, *J* = 7.6 Hz, 1H), 7.28–7.24 (m, 1H), 5.32 (s, 1H), 5.19–4.97 (m, 1H), 4.67 (d, *J* = 11.9 Hz, 1H), 4.49–4.40 (m, 2H), 3.87 (dd, *J* = 14.0, 5.6 Hz, 1H), 2.94 (dd, *J* = 11.5, 4.2 Hz, 1H), 2.11–2.02 (m, 2H), 1.98–1.91 (m, 2H), 1.78 (d, *J* = 13.6 Hz, 2H), 1.67 (d, *J* = 13.0 Hz, 1H), 1.58 (d, *J* = 6.2 Hz, 5H), 1.54 (s, 1H), 1.49–1.31 (m, 3H), 1.25 (s, 1H), 1.19 (s, 3H), 1.18–1.09 (m, 3H), 1.07 (s, 3H), 1.02 (s, 3H), 0.97 (s, 3H), 0.91 (s, 1H), 0.87 (d, *J* = 5.9 Hz, 6H), 0.80 (d, *J* = 3.4 Hz, 3H), 0.76 (d, *J* = 11.6 Hz, 1H); ${}^{13}$C NMR (100 MHz, CDCl${}_{3}$): δ 143.38 (C-13), 139.57, 128.25, 127.50, 127.27 (aromatic carbons), 123.81 (C-12), 86.57, 71.46, 63.08, 58.28, 58.25, 55.77, 47.65, 44.24, 44.18, 41.66, 40.13, 39.03, 38.65, 37.17, 37.00, 31.97, 30.81, 28.40, 26.42, 25.64, 23.79, 23.48, 23.46, 23.35, 22.86, 18.35, 17.03, 16.73, 15.62 (8×CH${}_{3}$); HRMS calculated for C${}_{41}$H${}_{62}$O${}_{2}$N${}_{3}$ [M + H]${}^{+}$ 628.4842, found 628.4842.

**2-(1-(((4a*****S*****,6a*****S*****,6b*****R*****,12a*****R*****)-10-(benzyloxy)-2,2,6a,6b,9,9,12a-heptamethyl-1,3,4,5,6,6a, 6b,7,8,8a,9,10,11,12,12a,12b,13,14b-octadecahydropicen-4a(2*****H*****)-yl)methyl)-1*****H*****-1,2,3-triazol- 4-yl)propan-2-ol (2i)**. Yield: 78%. White foamy solid, m.p. 123.3–124.4 ${}^{\circ }C$. ${}^{1}$H NMR (400 MHz, CDCl${}_{3}$): δ 7.38 (s, 1H), 7.35 (d, *J* = 3.6 Hz, 2H), 7.32 (d, *J* = 7.7 Hz, 1H), 7.26 (d, *J* = 5.9 Hz, 1H), 5.32 (s, 1H), 4.67 (d, *J* = 11.9 Hz, 1H), 4.45 (t, *J* = 12.7 Hz, 2H), 3.86 (d, *J* = 14.1 Hz, 1H), 2.94 (dd, *J* = 11.6, 4.2 Hz, 1H), 2.07 (dd, *J* = 15.5, 9.1 Hz, 2H), 2.00–1.92 (m, 2H), 1.84–1.76 (m, 2H), 1.71 (d, *J* = 17.8 Hz, 1H), 1.63 (s, 6H), 1.61–1.55 (m, 3H), 1.55–1.48 (m, 2H), 1.49–1.43 (m, 1H), 1.41 (s, 1H), 1.29–1.22 (m, 2H), 1.19 (s, 3H), 1.12 (dd, *J* = 15.3, 7.3 Hz, 3H), 1.07 (s, 3H), 1.04 (d, *J* = 4.9 Hz, 1H), 1.02 (s, 3H), 0.97 (s, 3H), 0.95–0.89 (m, 2H), 0.87 (d, *J* = 6.4 Hz, 6H), 0.79 (s, 3H), 0.75 (s, 1H); ${}^{13}$C NMR (100 MHz, CDCl${}_{3}$): δ 155.04, 143.41 (C-13), 139.59, 128.25, 127.50, 123.81, 120.49 (aromatic carbons), 127.27 (C-12), 86.57, 71.45, 68.58, 60.45, 58.23, 55.79, 47.66, 44.19, 41.67, 40.15, 39.04, 38.67, 37.19, 37.01, 33.11, 31.97, 30.82, 30.66, 30.59, 28.41, 26.43, 25.64, 23.80, 23.44, 22.87, 18.36, 17.03, 16.73, 15.63 (9×CH${}_{3}$); HRMS calculated for C${}_{42}$H${}_{63}$O${}_{2}$N${}_{3}$ [M + H]${}^{+}$ 642.4999, found 642.5000.

### 3.3. Fungicidal Assays

The antifungal activity was measured by a mycelial growth rate assay. The sample compounds were seperately weighed at 50 mg, dissolved in 10 mL DMSO (5 g/L), and then the solution was prepared into a drug solution for testing. The drug solution and potato dextrose agar (PDA) were uniformly mixed to prepare a toxic medium at a concentration of 50 μg/mL. Chlorothalonil was used as the drug control, and a blank control was set at the same time, with three repetitions. The culture temperature was 24 ± 0.5 °C. After the colonies in the blank control were sufficiently grown, the diameters of the colonies of each treatment were measured by the cross method, and the average value was taken.

## 4. Conclusions

According to the structural characteristics of the reported inhibitor of glucosamine-6-phosphate synthase (GlmS), and based on the concept of developing a natural green pesticide lead compound, the structurally similar natural product oleanolic acid was used as the parent molecule. According to the principle of active substructure splicing, 18 oleanane-type conjugates with 1,2,3-Triazole fragment were designed and synthesized by introducing the effective and active group triazole structure of pesticides. In addition, all of the synthesized structures were new compounds. The structure of the synthesized triazole compound and some intermediates was confirmed by ${}^{1}$H-NMR, ${}^{13}$C-NMR and HRMS, and the results were in accordance with the intended design idea.

The results of the antifungal activity assay showed that the oleanane-type conjugates with 1,2,3-Triazole fragment had certain antifungal activity against the six pathogenic fungi at a concentration of 50 μg/mL. The target compounds demonstrated conspicuous inhibitory effects on ***Sclerotinia sclerotiorum***, ***Botrytis cinerea Pers*** and ***Rhizoctonia solani Kuhn***, with an inhibition rate higher than 50%. Compared with the raw material oleanolic acid OA, the antifungal activity of the target was significantly improved. The antifungal effects of compounds **1e**, **1f**, **1g**, **2e**, **2f** and **2g** on ***Sclerotinia sclerotiorum*** were particularly prominent, 85.6%, 83.1%, 87.6%, 86.8%, 87.7%, and 89.6%, respectively. Structural analysis of the compound revealed that the benzene ring (e.g., fluorine, chlorine, nitro) of the electron-withdrawing substituent increases the activity. Subsequent work will further explore the development of new lead compounds through the study of structure–activity relationships, laying the experimental foundation for the research of novel glucosamine-6-phosphate synthase inhibitors.

## 5. Patents

Zhao, H.Q; Chen, Z.L; Xu, C; Zhao, W.T; Zhao, J.Z; Hu; P.Y. Preparation Method and Application of Oleanolic Acid Derivative. China Patent, CN: 107857791A, 2018-03-30.

## Figures and Tables

**Table 1:** Physical data of intermediate products and target compounds.

Compd.	R${}_{1}$	R${}_{2}$	Formula	Status	m.p./°C	Yield (%)
**3**	-CH${}_{3}$	-	C${}_{31}$H${}_{52}$O${}_{2}$	White foamy solid	293.0–293.5	92
**4**	Bn	-	C${}_{37}$H${}_{56}$O${}_{2}$	White foamy solid	228.0-228.5	90
**5**	-CH${}_{3}$	-	C${}_{31}$H${}_{51}$N${}_{3}$O	White foamy solid	175.5–177.2	87
**6**	Bn	-	C${}_{37}$H${}_{55}$N${}_{3}$O	White foamy solid	158.5–160.1	89
**1a**	-CH${}_{3}$	C${}_{6}$H${}_{5}$-	C${}_{39}$H${}_{57}$ON${}_{3}$	White foamy solid	268.4–270.5	82
**1b**	-CH${}_{3}$	3-NH${}_{2}$-C${}_{6}$H${}_{4}$-	C${}_{39}$H${}_{58}$ON${}_{4}$	White foamy solid	239.4–240.8	84
**1c**	-CH${}_{3}$	4-CH${}_{3}$O-C${}_{6}$H${}_{4}$-	C${}_{40}$H${}_{59}$O${}_{2}$N${}_{3}$	White foamy solid	114.4–114.6	86
**1d**	-CH${}_{3}$	3-CH${}_{3}$-C${}_{6}$H${}_{4}$-	C${}_{40}$H${}_{59}$ON${}_{3}$	White foamy solid	230.1–230.6	84
**1e**	-CH${}_{3}$	4-Cl-C${}_{6}$H${}_{4}$-	C${}_{39}$H${}_{56}$ON${}_{3}$Cl	White foamy solid	99.5–105.2	79
**1f**	-CH${}_{3}$	4-NO${}_{2}$-C${}_{6}$H${}_{4}$-	C${}_{39}$H${}_{56}$O${}_{3}$N${}_{4}$	White foamy solid	253.2–256.7	83
**1g**	-CH${}_{3}$	4-F-C${}_{6}$H${}_{4}$-	C${}_{39}$H${}_{56}$ON${}_{3}$F	White foamy solid	232.7–236.2	81
**1h**	-CH${}_{3}$	CH${}_{3}$-CHOH-	C${}_{35}$H${}_{57}$O${}_{2}$N${}_{3}$	White foamy solid	104.1–108.7	79
**1i**	-CH${}_{3}$	(CH${}_{3}$)${}_{2}$-C(OH)-	C${}_{36}$H${}_{59}$O${}_{2}$N${}_{3}$	Oily	——	77
**2a**	Bn	C${}_{6}$H${}_{5}$-	C${}_{45}$H${}_{61}$ON${}_{3}$	White foamy solid	252.6–255.9	82
**2b**	Bn	3-NH${}_{2}$-C${}_{6}$H${}_{4}$-	C${}_{45}$H${}_{62}$ON${}_{4}$	White foamy solid	112.8–113.0	84
**2c**	Bn	4-CH${}_{3}$O-C${}_{6}$H${}_{4}$-	C${}_{46}$H${}_{63}$O${}_{2}$N${}_{3}$	White foamy solid	125.6–127.0	79
**2d**	Bn	3-CH${}_{3}$-C${}_{6}$H${}_{4}$-	C${}_{46}$H${}_{63}$ON${}_{3}$	White foamy solid	220.8–223.7	78
**2e**	Bn	4-Cl-C${}_{6}$H${}_{4}$-	C${}_{45}$H${}_{60}$ON${}_{3}$Cl	White foamy solid	111.0–113.3	74
**2f**	Bn	4-NO${}_{2}$-C${}_{6}$H${}_{4}$-	C${}_{45}$H${}_{60}$O${}_{3}$N${}_{4}$	White foamy solid	142.1–143.9	79
**2g**	Bn	4-F-C${}_{6}$H${}_{4}$-	C${}_{45}$H${}_{60}$O${}_{2}$N${}_{3}$F	White foamy solid	205.2–208.5	76
**2h**	Bn	CH${}_{3}$-CHOH-	C${}_{41}$H${}_{61}$O${}_{2}$N${}_{3}$	White foamy solid	126.8–134.6	89
**2i**	Bn	(CH${}_{3}$)${}_{2}$-C(OH)-	C${}_{42}$H${}_{63}$O${}_{2}$N${}_{3}$	White foamy solid	123.3–124.4	78

**Table 2:** Inhibition rate of target compounds against six fungus species (% control at 50 μg/mL).

Compds No.	Inhibition Ratio (100%)					
	***Sclerotinia*** *sclerotiorum*	*Phytophthora* *boehmeriae* *Saw *	*Botrytis* *cinerea* *Pers *	*Rhizoctonia* *solani* *Kuhn *	*Pyricularia* *oryzae* *Cav. *	*Fusarium* *oxysporum Schl.* *F.sp.vasinfectum* *(Atk.) Snyd. & Hans.*
**1a**	70.1	50.3	75.6	70.4	40.2	30.5
**1b**	61.2	46.8	68.5	64.3	35.2	32.6
**1c**	69.8	49.9	60.2	63.8	36.6	28.4
**1d**	68.5	53.4	60.9	60.4	38.1	29.9
**1e**	85.6	66.8	66.9	69.6	39.4	31.4
**1f**	83.1	60.8	70.5	72.5	37.8	27.6
**1g**	87.6	57.9	71.6	75.6	36.6	23.5
**1h**	61.5	50.8	68.7	65.2	30.1	20.8
**1i**	65.4	49.6	59.1	69.4	28.1	19.9
**2a**	72.5	40.8	65.8	69.8	26.5	23.5
**2b**	67.3	46.9	63.5	61.5	24.8	18.9
**2c**	66.7	40.5	59.8	60.7	23.9	19.7
**2d**	68.7	43.8	56.8	66.4	26.8	17.8
**2e**	86.8	50.8	75.9	75.8	20.5	23.8
**2f**	87.7	55.7	77.4	78.9	22.4	26.7
**2g**	89.6	54.1	78.9	79.7	30.5	25.4
**2h**	75.8	50.6	68.9	70.1	29.8	21.5
**2i**	77.9	48.7	67.4	68.7	26.8	22.9
OA	20.1	11.5	13.7	19.2	16.9	9.5
Chlorothalonil	92.7	94.2	98	98.4	89.2	94.2

**OA** represents oleanolic acid.

**Table 3:** Enzyme-inhibition Rate of Compounds **1** and **2** at 0.35 mm.

Compd.	Inhibition Rate (%)	Compd.	Inhibition Rate (%)
**1a**	17.6	**2a**	15.8
**1b**	15.1	**2b**	16.2
**1c**	19.1	**2c**	13.5
**1d**	13.8	**2d**	14.3
**1e**	27.8	**2e**	20.9
**1f**	19.6	**2f**	22.2
**1g**	23.2	**2g**	19.9
**1h**	20.1	**2h**	18.7
**1i**	18.3	**2i**	17.6
OA	21.8		

**OA** represents oleanolic acid.

## Data Availability

The data presented in this study are available on request from the corresponding author.

## Supplementary Materials

The following are available online at https://www.mdpi.com/article/10.3390/molecules27154928/s1, Figures S1–S60: ${}^{1}$H NMR, ${}^{1}$${}^{3}$C NMR and HRMS spectra including intermediate compounds and target compounds.
