# Supplementary material for: Oleanane-Type Triterpene Conjugates with 1H-1,2,3-Triazole Possessing of Fungicidal Activity
Source: Molecules. 2022 Aug 2;27(15):4928. doi: 10.3390/molecules27154928 (PMC9370531; doi:10.3390/molecules27154928)

Article

Oleanane -Type Triterpene Conjugates with 1*H*-1,2,3-Triazole Possessing of Fungicidal Activity

Zili Chen[†] [1], Yu Jiang[†] [1], Chen Xu[1], Xiangyu Sun[2], Chao Ma[3], Zihao Xia[*] [1] and Hanqing Zhao[*] [1]

[1] Key Laboratory for Northern Urban Agriculture of Ministry of Agriculture and Rural Affairs, Beijing University of Agriculture, Beijing 102206,China; chenzili1995@163.com (Z.C.); jy1013937462@126.com(Y.J.); 13691458370@163.com (C.X.);

[2] Torch High Technology Industry Development Center, Ministry of Science &technology, Beijing 100045, China; sunxiangyu1990@126.com(X.S.);

[3] Beijing Zhong Bao Green Agriculture Technology Group Co.Ltd, Beijing 100193,China; racoon_happy@126.com(C.M.)

* Correspondence: 20209201@bua.edu.cn (Z.X.); zhaohanqingbua@163.com; Tel.: + 86-10-8079-9234; Fax: + 86-10-8079-9234 (H.Z.)

† These authors contributed equally to this work.

Supporting Information

Page No.

Spectrum section

1 ^{1}H NMR spectrum of compound **3** S-3
2 ^{13}C NMR spectrum of compound **3** S-3
3 ^{1}H NMR spectrum of compound **5** S-4
4 ^{13}C NMR spectrum of compound **5** S-4
5 ^{1}H NMR spectrum of compound **6** S-5
6 ^{13}C NMR spectrum of compound **6** S-5
7 ^{1}H NMR spectrum of compound **1a** S-6
8 ^{13}C NMR spectrum of compound **1a** S-6
9 HRMS spectrum of compound **1a** S-7
10 ^{1}H NMR spectrum of compound **1b** S-7
11 ^{13}C NMR spectrum of compound **1b** S-8
12 HRMS spectrum of compound **1b** S-8
13 ^{1}H NMR spectrum of compound **1c** S-9
14 ^{13}C NMR spectrum of compound **1c** S-9
15 HRMS spectrum of compound **1c** S-10
16 ^{1}H NMR spectrum of compound **1d** S-10
17 ^{13}C NMR spectrum of compound **1d** S-11
18 HRMS spectrum of compound **1d** S-11
19 ^{1}H NMR spectrum of compound **1e** S-12
20 ^{13}C NMR spectrum of compound **1e** S-12
21 HRMS spectrum of compound **1e** S-13
22 ^{1}H NMR spectrum of compound **1f** S-13

23 ^{13}C NMR spectrum of compound **1f** S-14
24 HRMS spectrum of compound **1f** S-14
25 ^{1}H NMR spectrum of compound 1**g** S-15
26 ^{13}C NMR spectrum of compound **1g** S-15
27 HRMS spectrum of compound **1g** S-16
28 ^{1}H NMR spectrum of compound **1h** S-16
29 ^{13}C NMR spectrum of compound **1h** S-17
30 HRMS spectrum of compound **1h** S-17
31 ^{1}H NMR spectrum of compound **1i** S-18
32 ^{13}C NMR spectrum of compound **1i** S-18
33 HRMS spectrum of compound **1i** S-19
34 ^{1}H NMR spectrum of compound **2a** S-19
35 ^{13}C NMR spectrum of compound **2a** S-20
36 HRMS spectrum of compound **2a** S-20
37 ^{1}H NMR spectrum of compound **2b** S-21
38 ^{13}C NMR spectrum of compound **2b** S-21
39 HRMS spectrum of compound **2b** S-22
40 ^{1}H NMR spectrum of compound **2c** S-22
41 ^{13}C NMR spectrum of compound **2c** S-23
42 HRMS spectrum of compound **2c** S-23
43 ^{1}H NMR spectrum of compound **2d** S-24
44 ^{13}C NMR spectrum of compound **2d** S-24
45 HRMS spectrum of compound **2d** S-25
46 ^{1}H NMR spectrum of compound **2e** S-25
47 ^{13}C NMR spectrum of compound **2e** S-26
48 HRMS spectrum of compound **2e** S-26
49 ^{1}H NMR spectrum of compound **2f** S-27
50 ^{13}C NMR spectrum of compound **2f** S-27
51 HRMS spectrum of compound **2f** S-28
52 ^{1}H NMR spectrum of compound **2g** S-28
53 ^{13}C NMR spectrum of compound **2g** S-29
54 HRMS spectrum of compound **2g** S-29
55 ^{1}H NMR spectrum of compound **2h** S-30
56 ^{13}C NMR spectrum of compound **2h** S-30
57 HRMS spectrum of compound **2h** S-31
58 ^{1}H NMR spectrum of compound **2i** S-31
59 ^{13}C NMR spectrum of compound **2i** S-32
60 HR4MS spectrum of compound **2i** S-32

^{1}H NMR spectrum of compound **3**

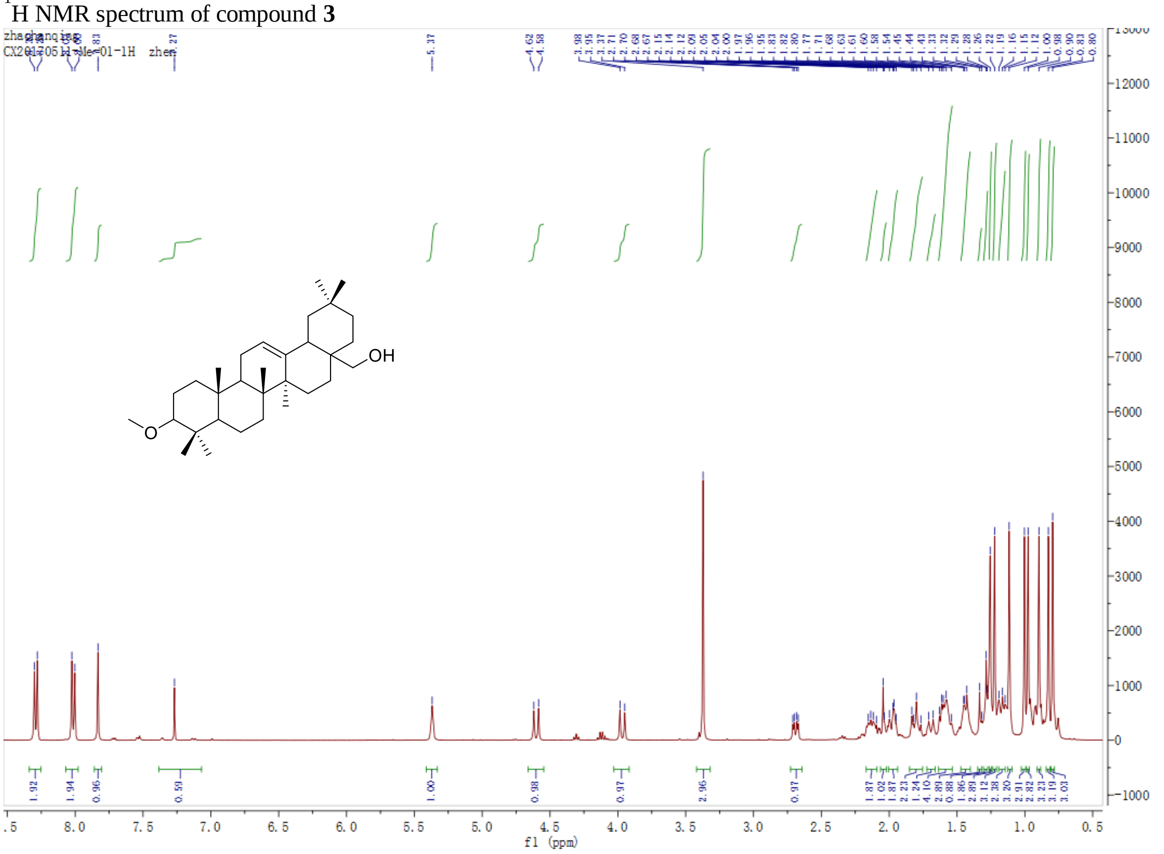

^{13}C NMR spectrum of compound **3**

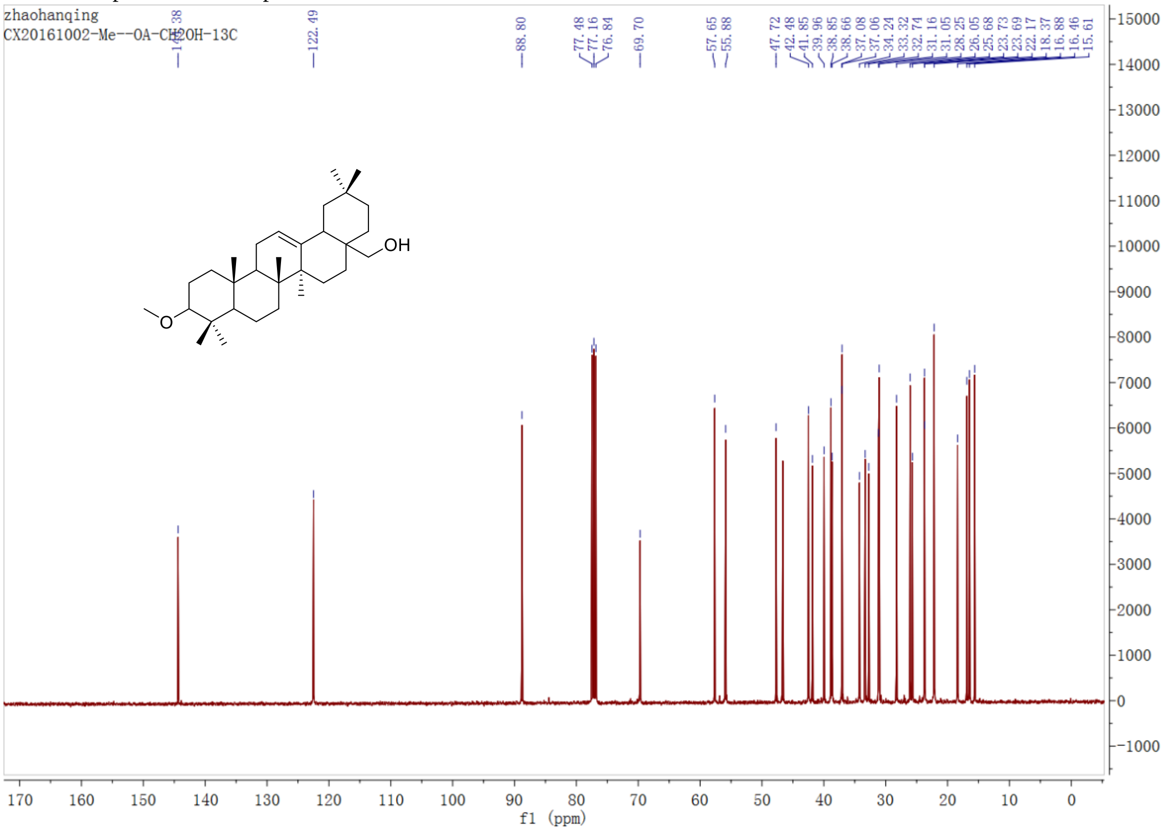

^{1}H NMR spectrum of compound **5**

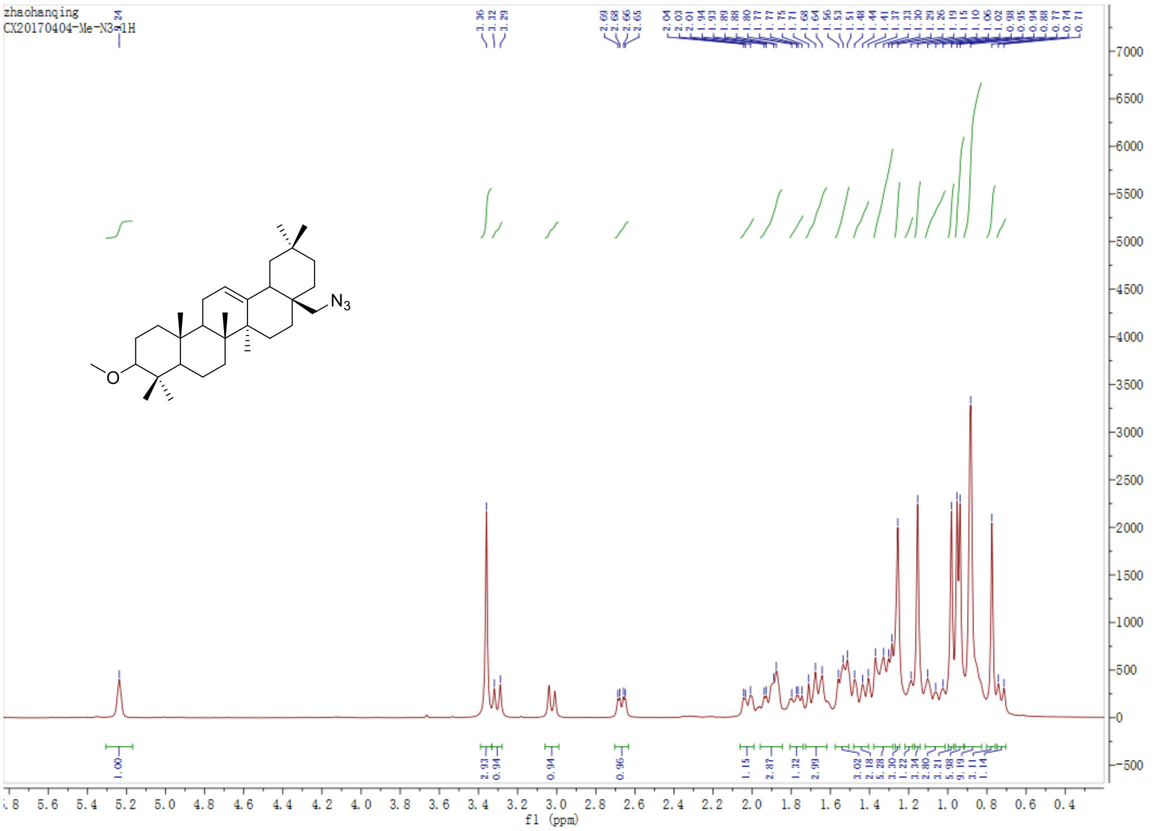

^{13}C NMR spectrum of compound **5**

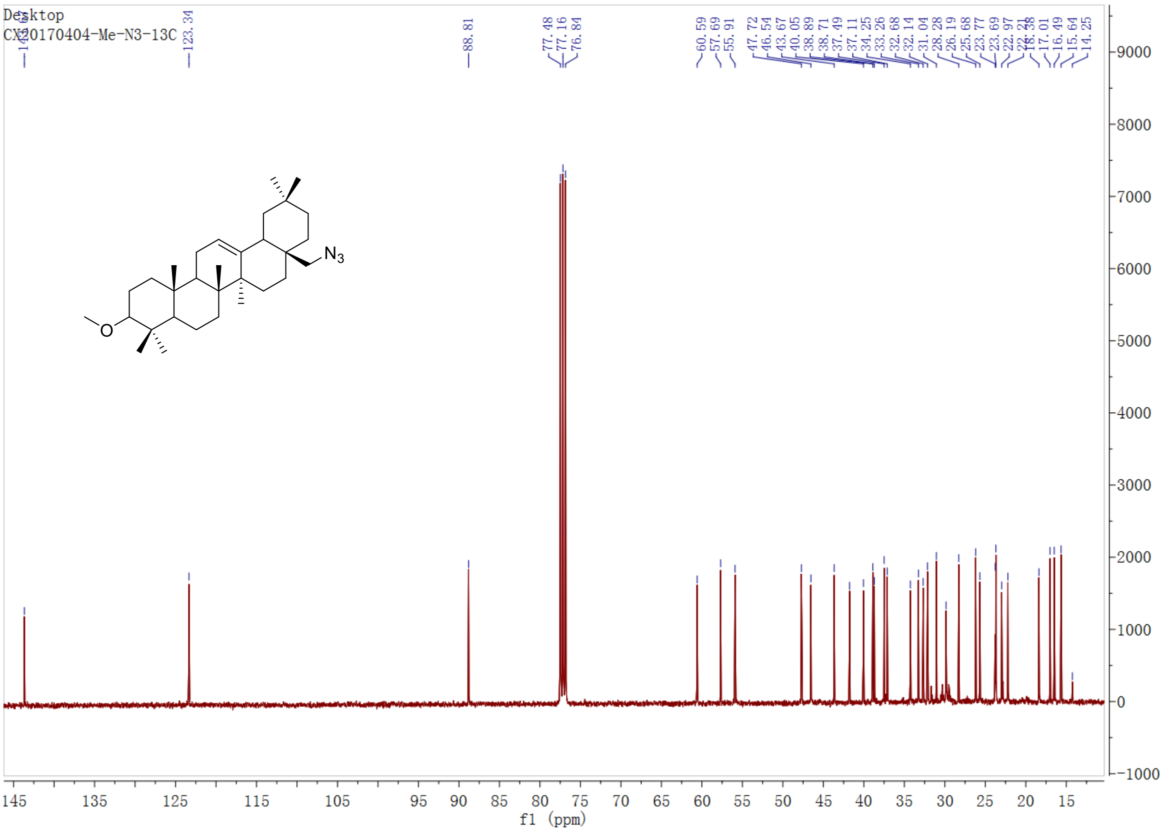

^{1}H NMR spectrum of compound **6**

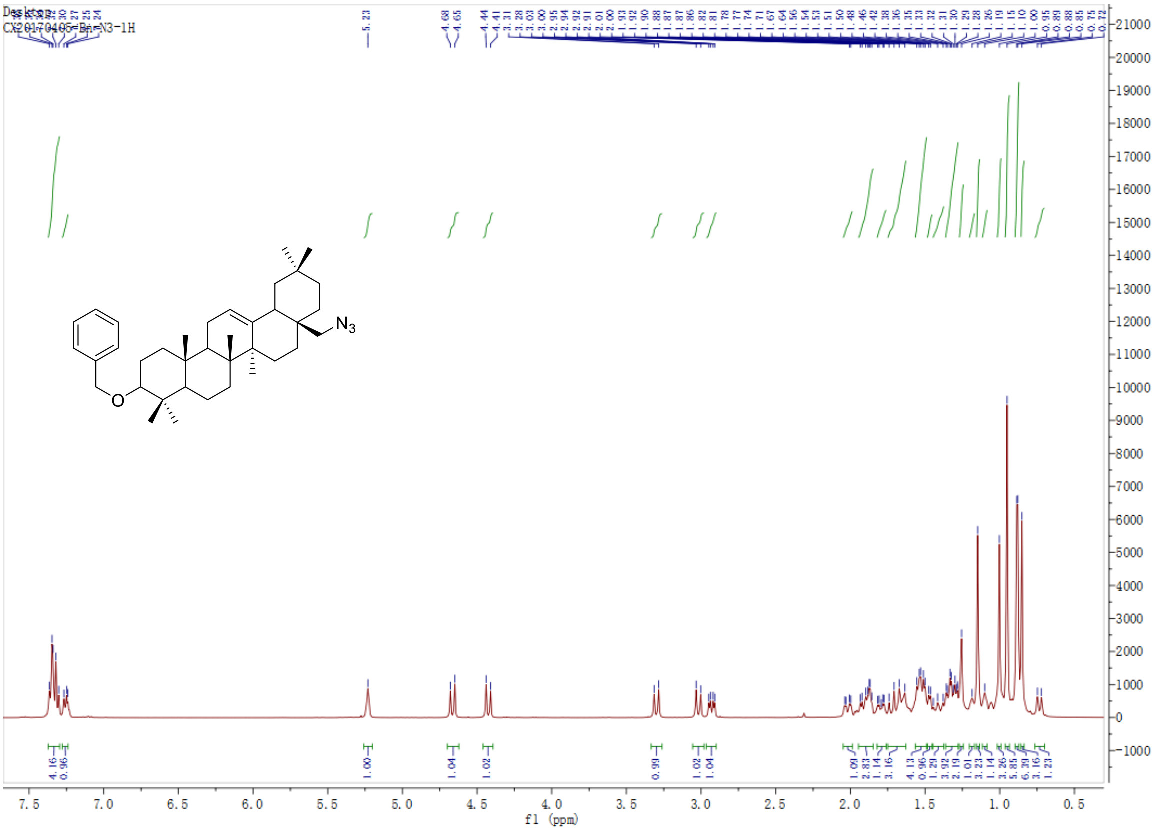

^{13}C NMR spectrum of compound **6**

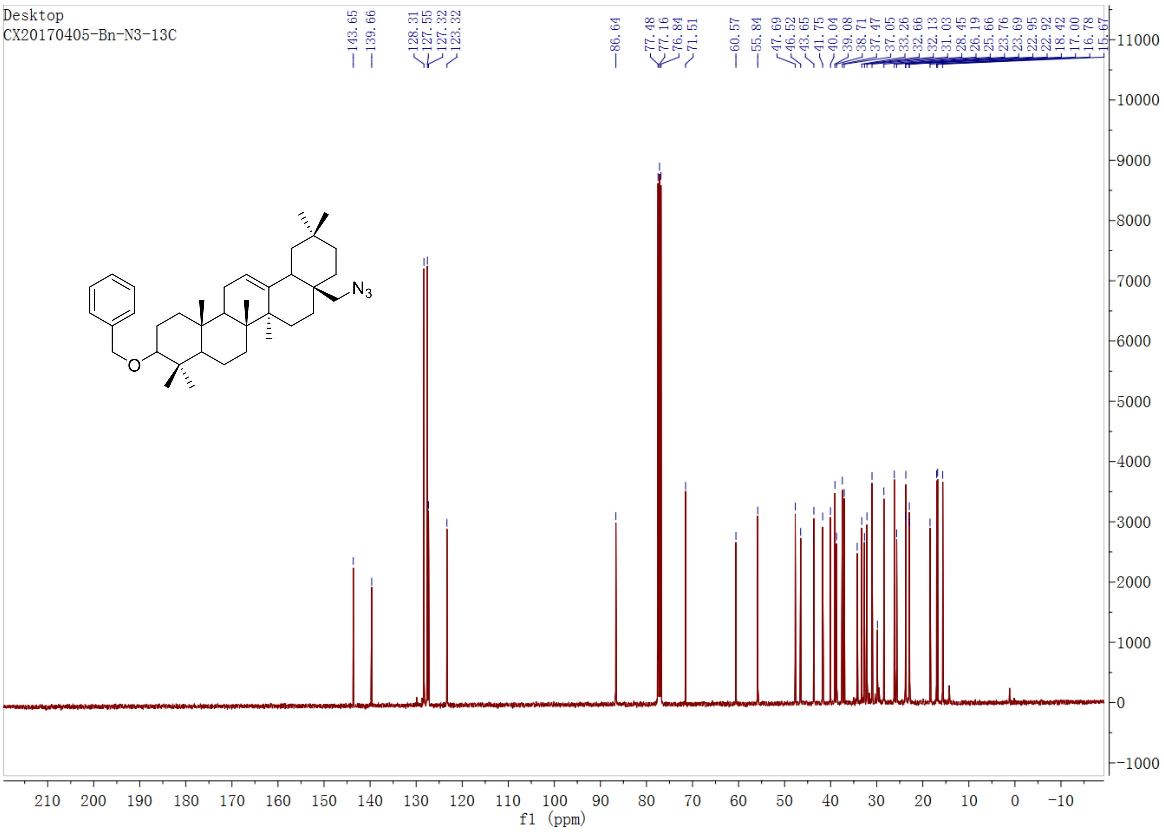

^{1}H NMR spectrum of compound **1a**

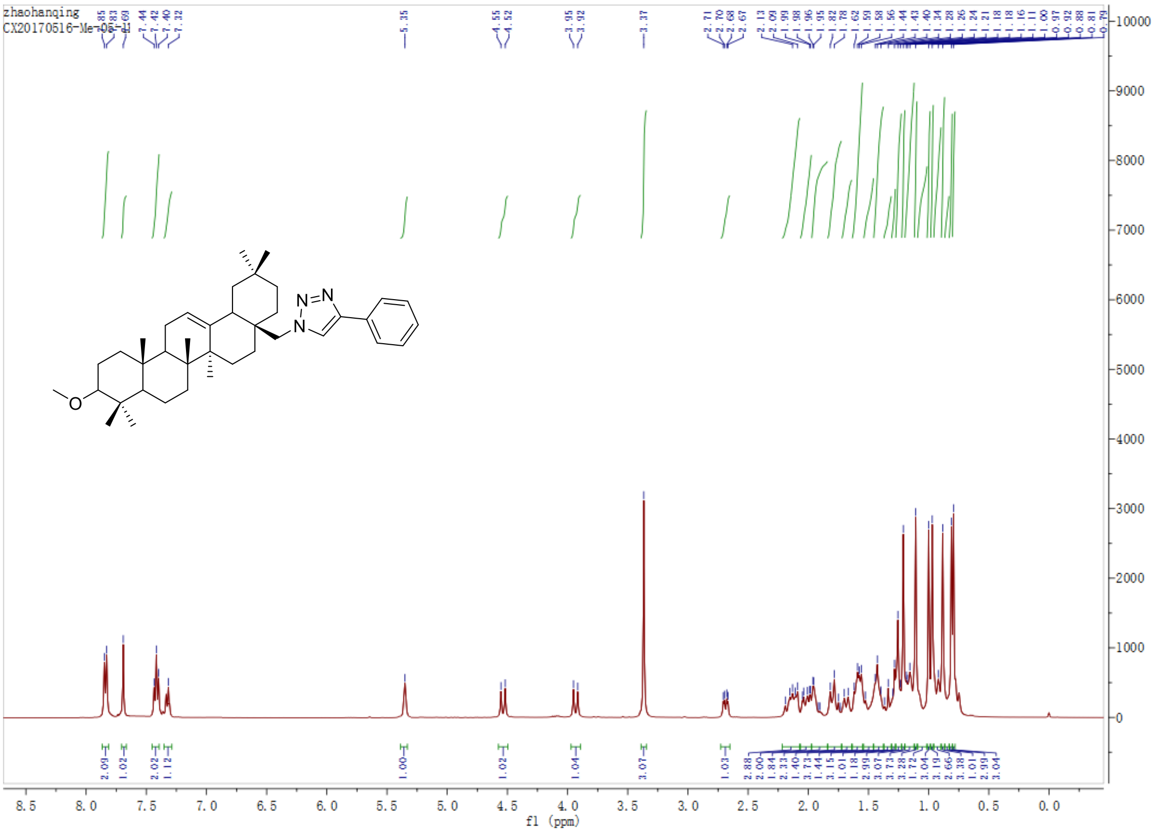

^{13}C NMR spectrum of compound **1a**

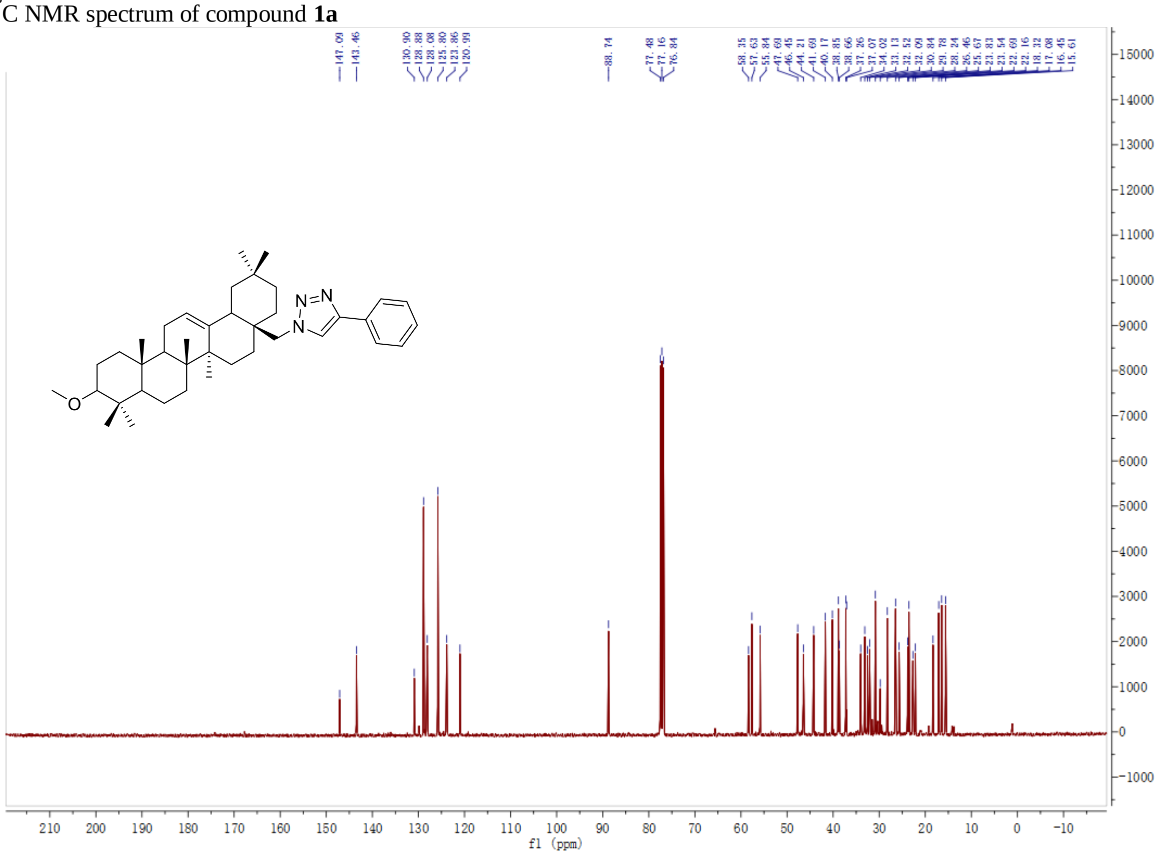

HRMS spectrum of compound **1a**

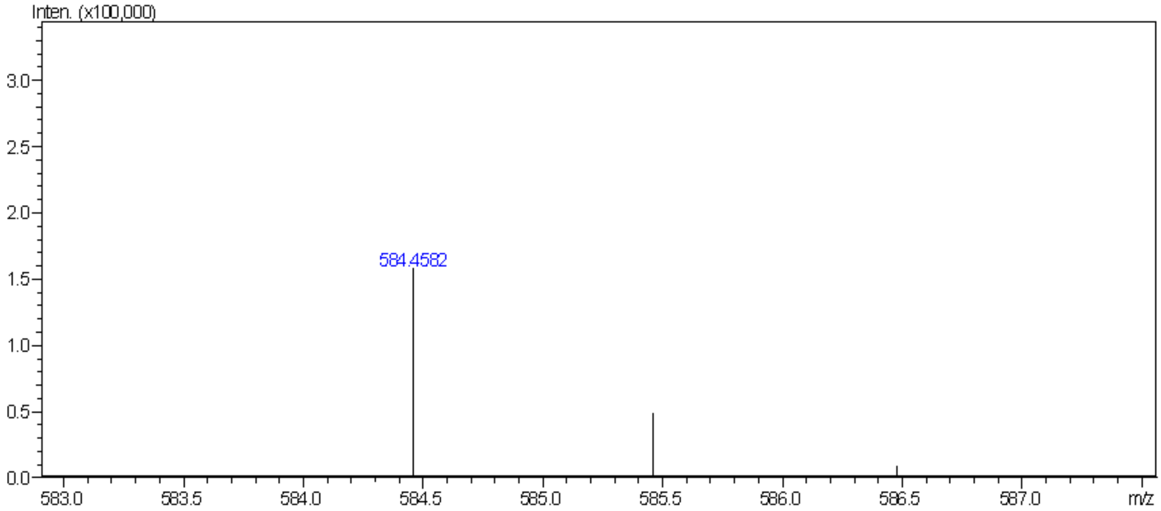

^{1}H NMR spectrum of compound **1b**

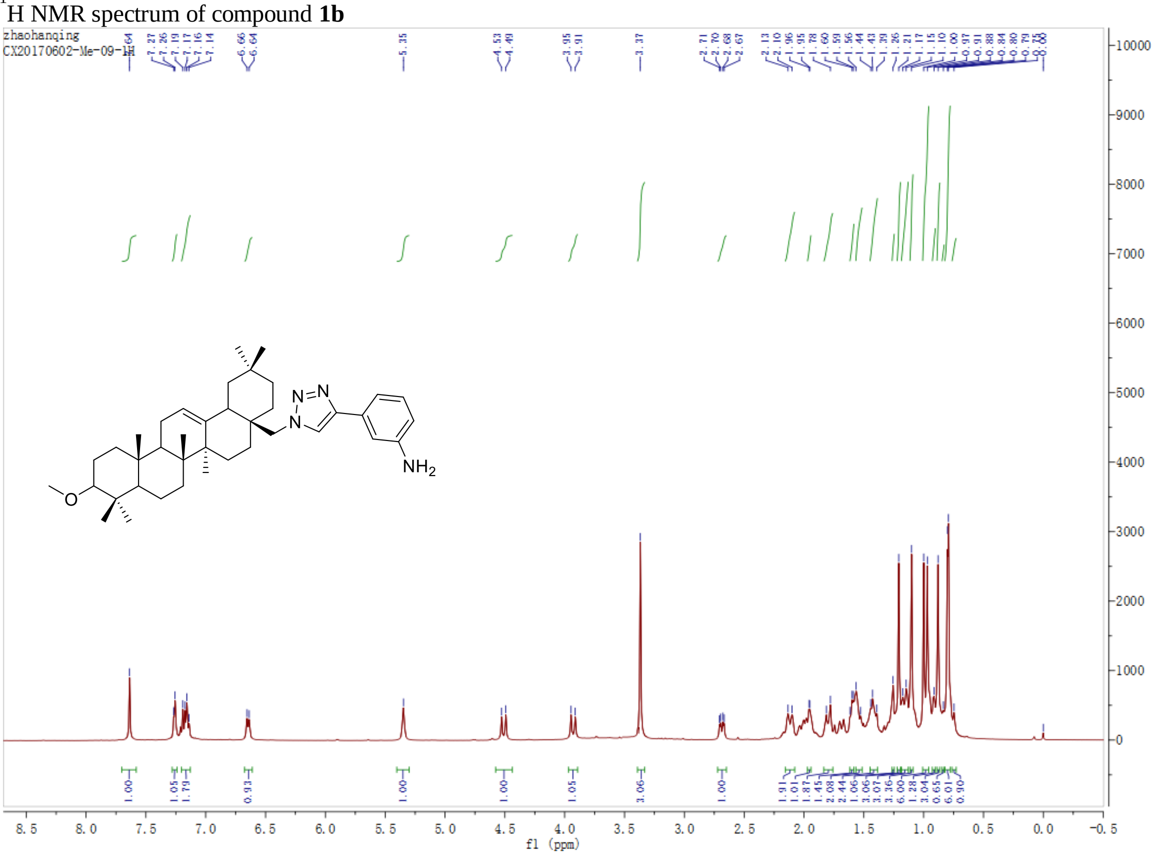

^{13}C NMR spectrum of compound **1b**

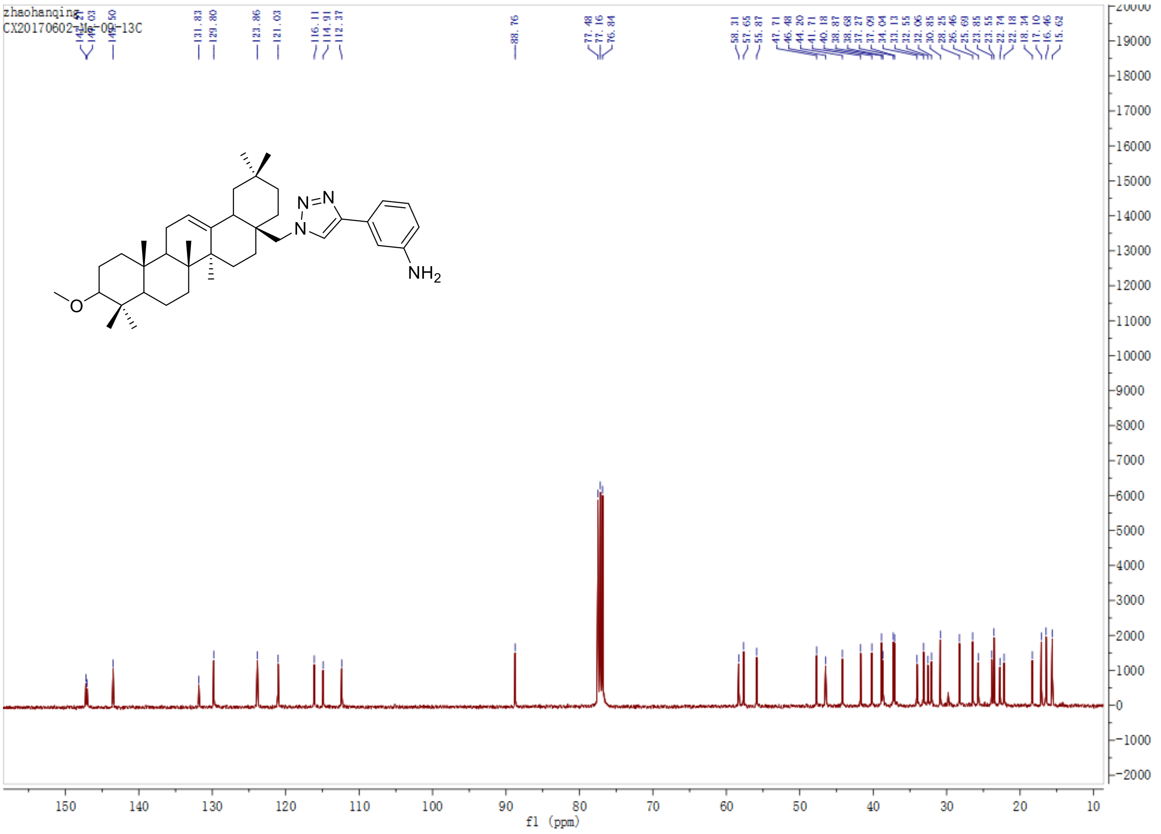

HRMS spectrum of compound **1b**

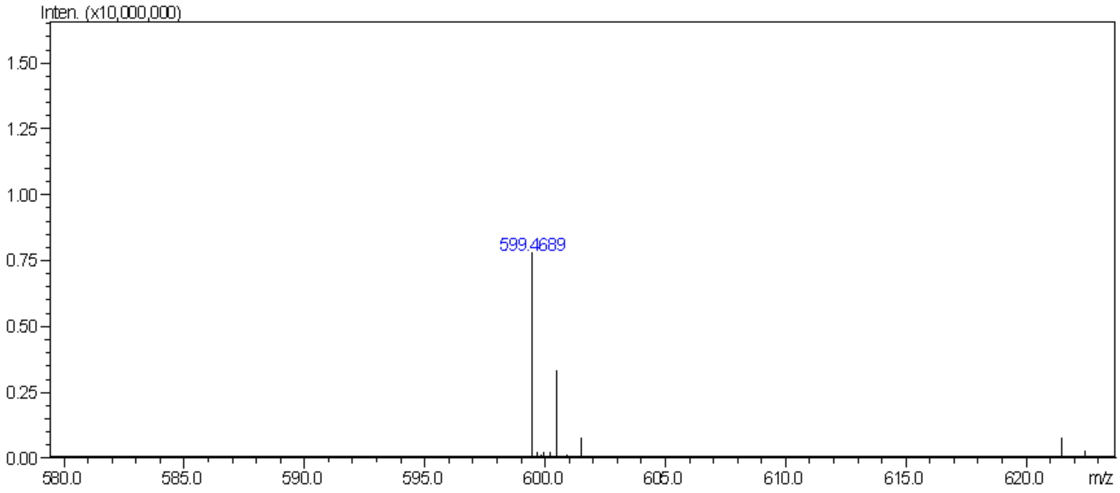

^{1}H NMR spectrum of compound **1c**

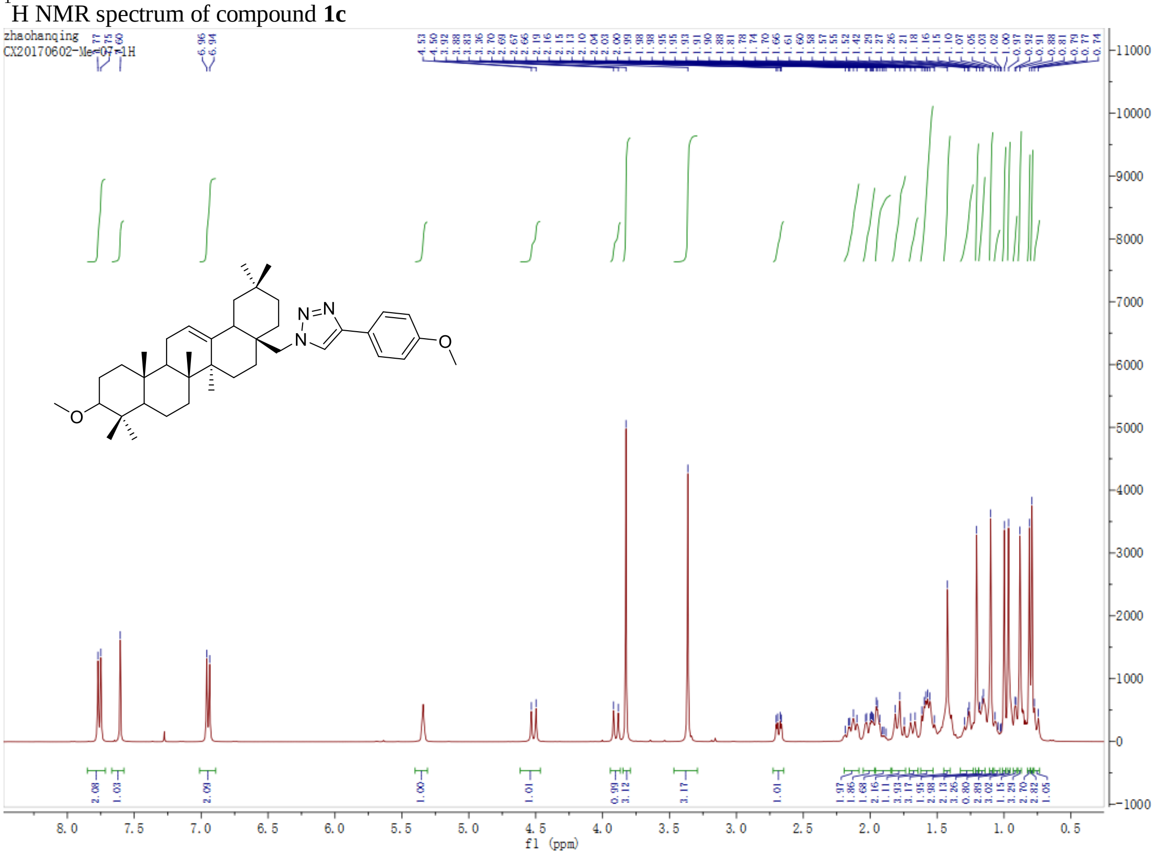

^{13}C NMR spectrum of compound **1c**

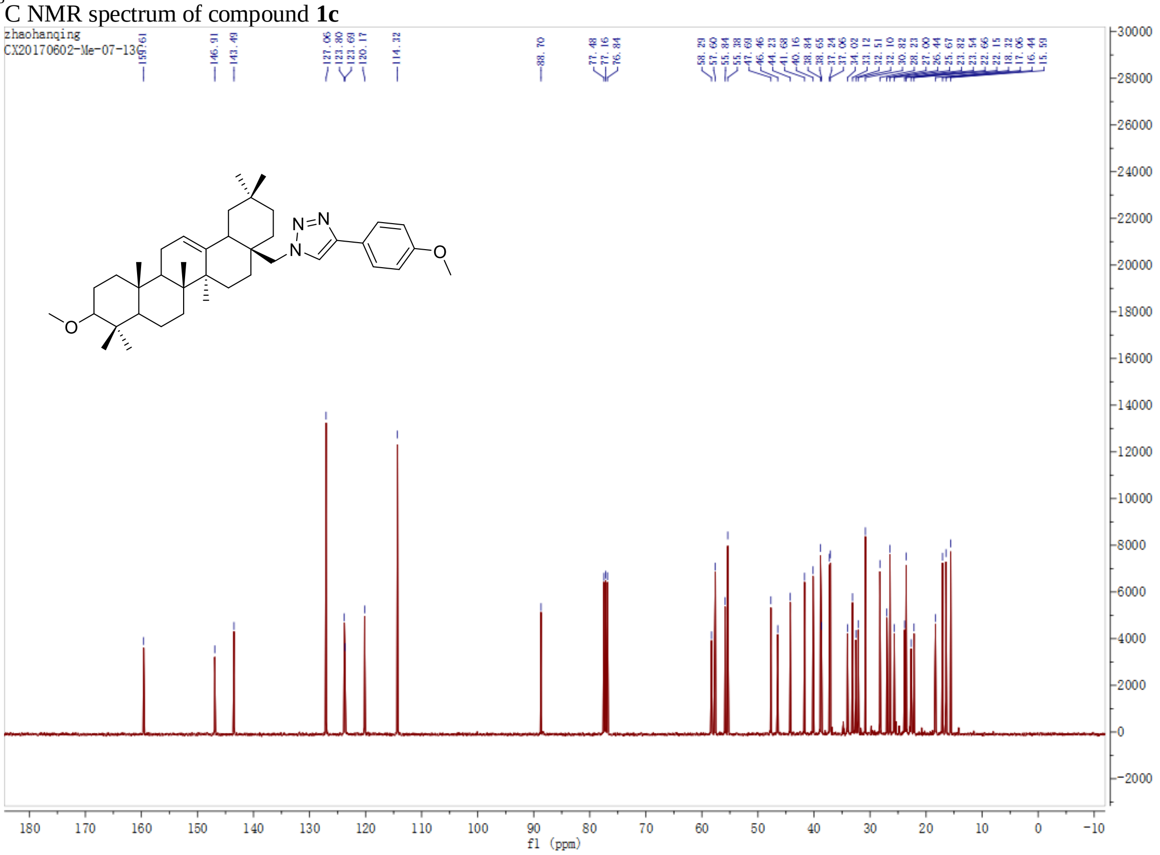

HRMS spectrum of compound **1c**

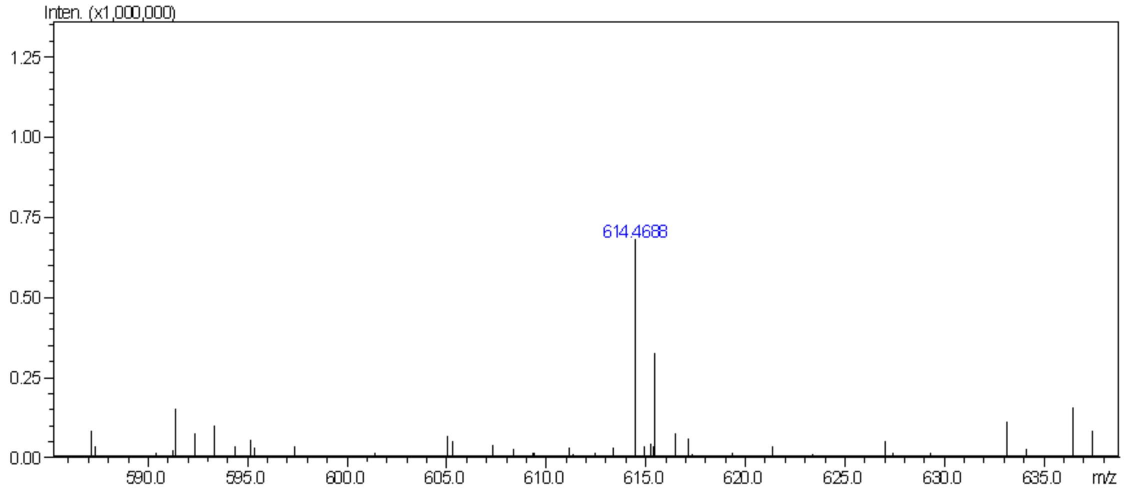

^{1}H NMR spectrum of compound **1d**

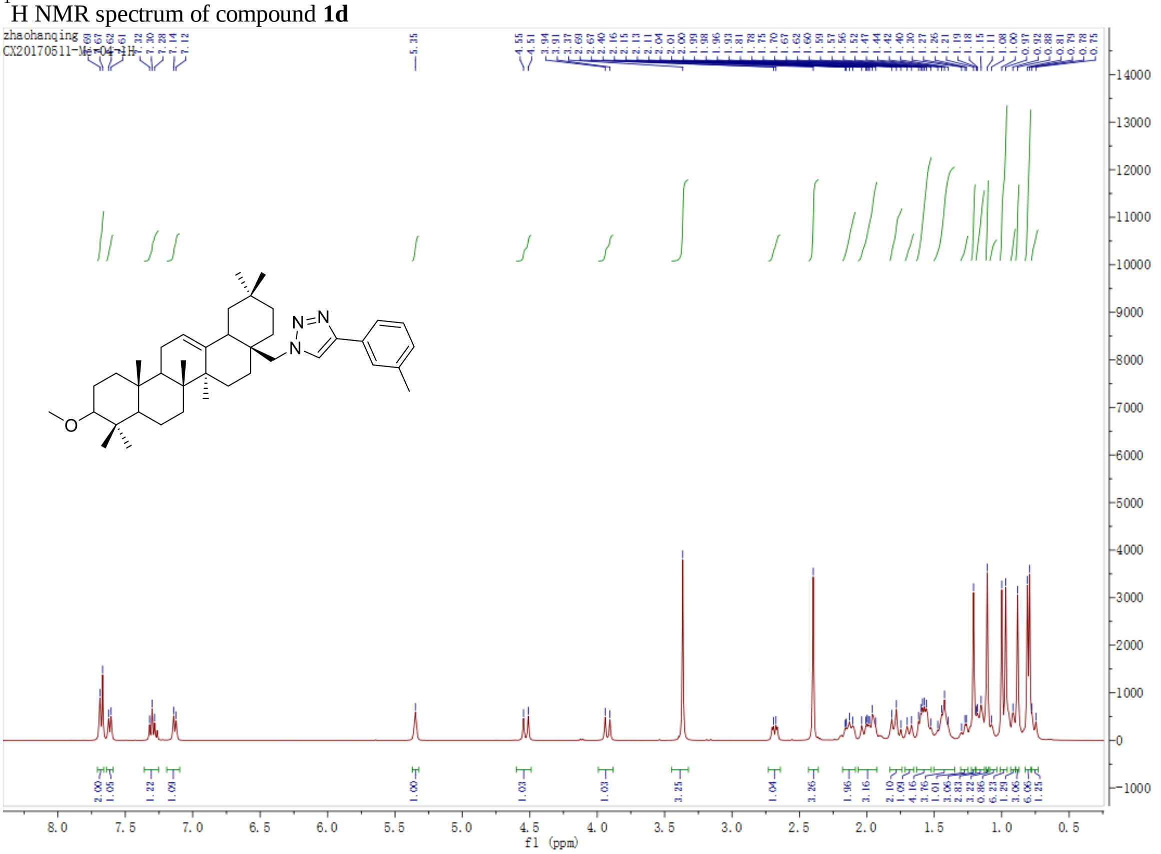

^{13}C NMR spectrum of compound **1d**

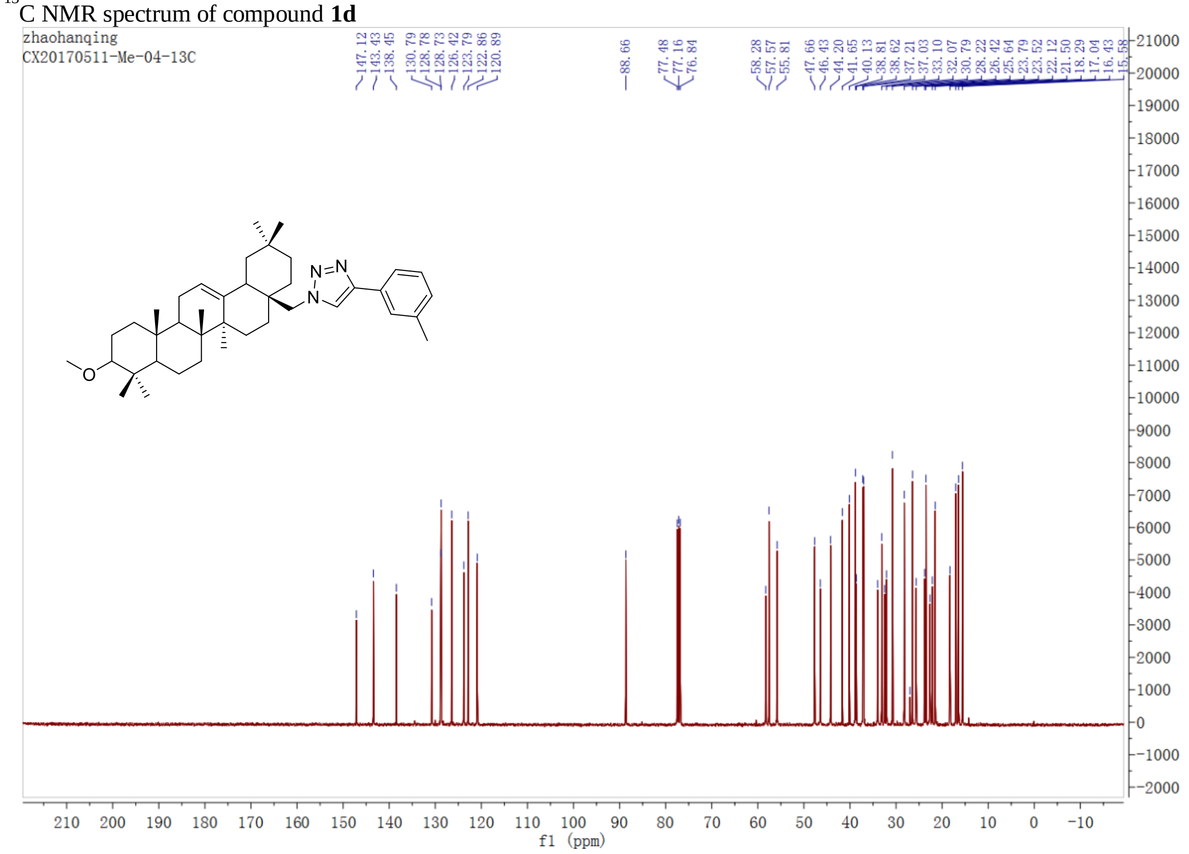

HRMS spectrum of compound **1d**

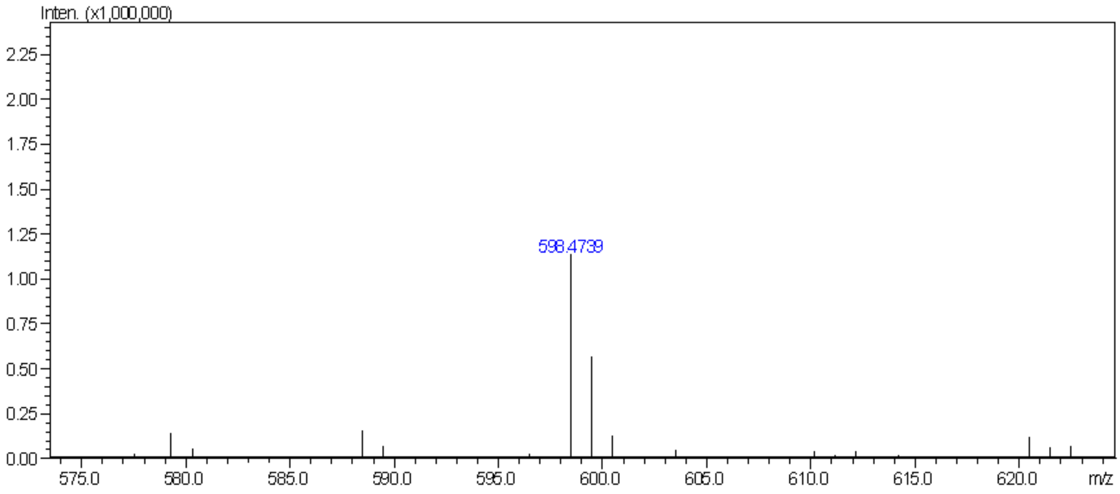

^{1}H NMR spectrum of compound **1e**

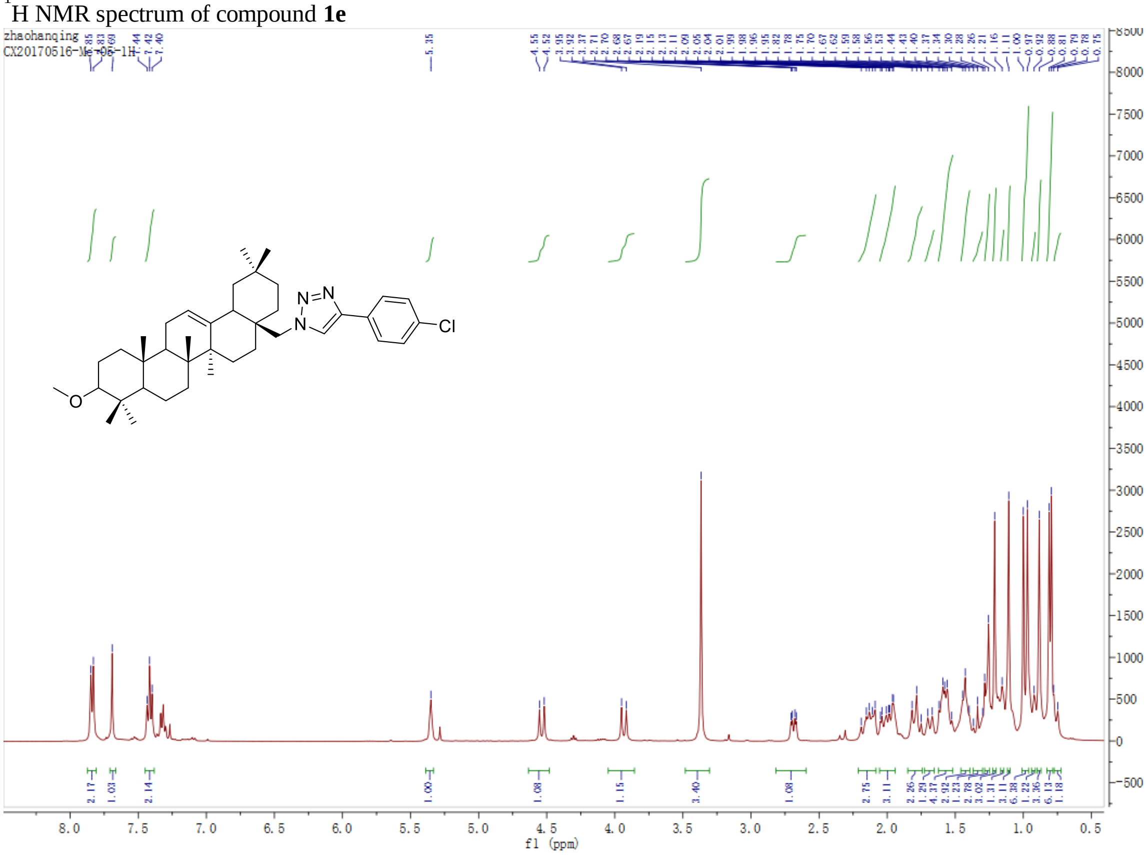

^{13}C NMR spectrum of compound **1e**

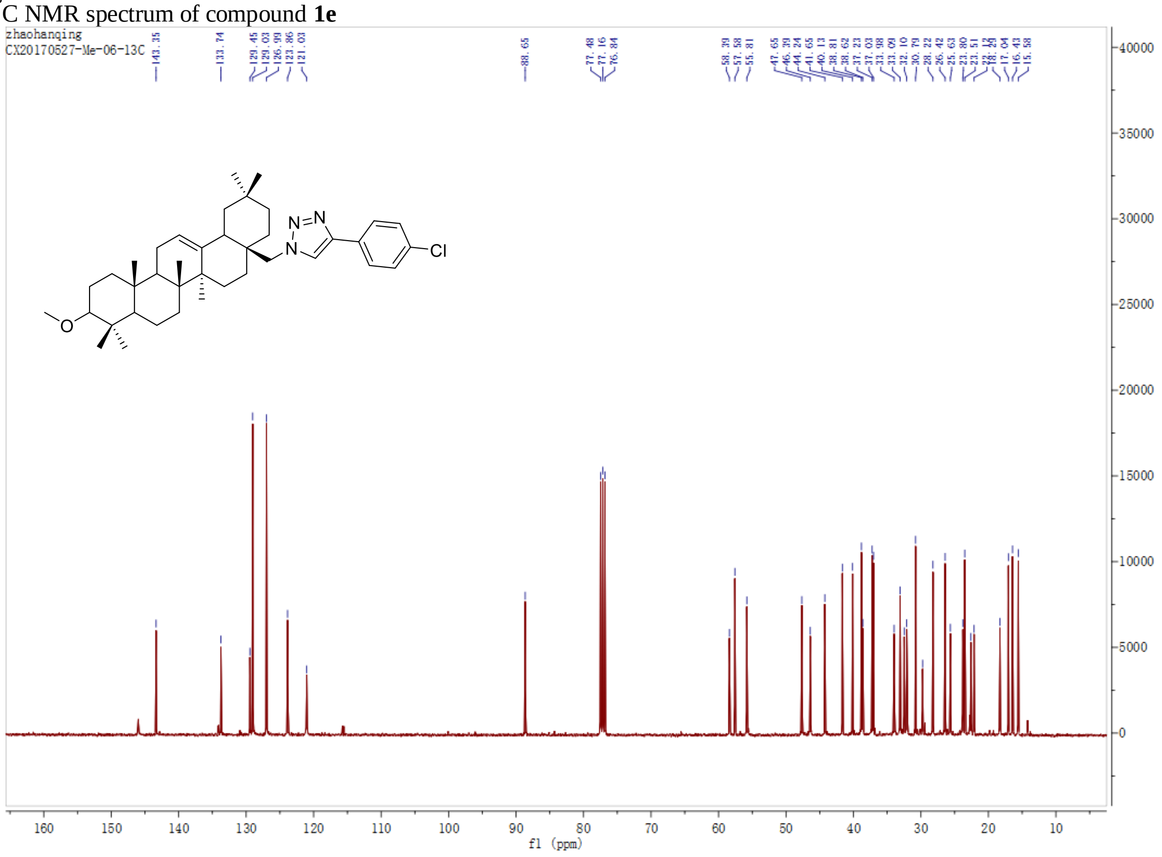

HRMS spectrum of compound **1e**

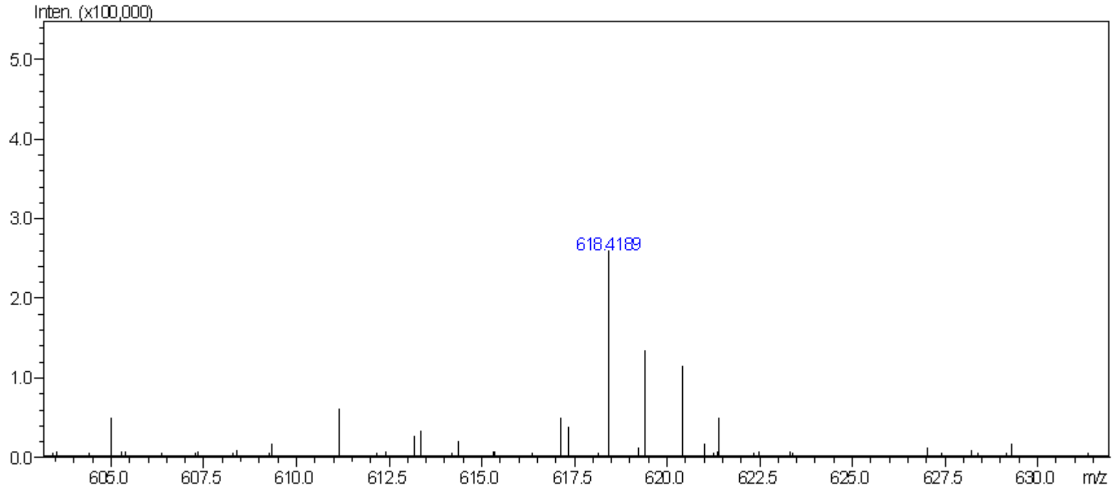

^{1}H NMR spectrum of compound **1f**

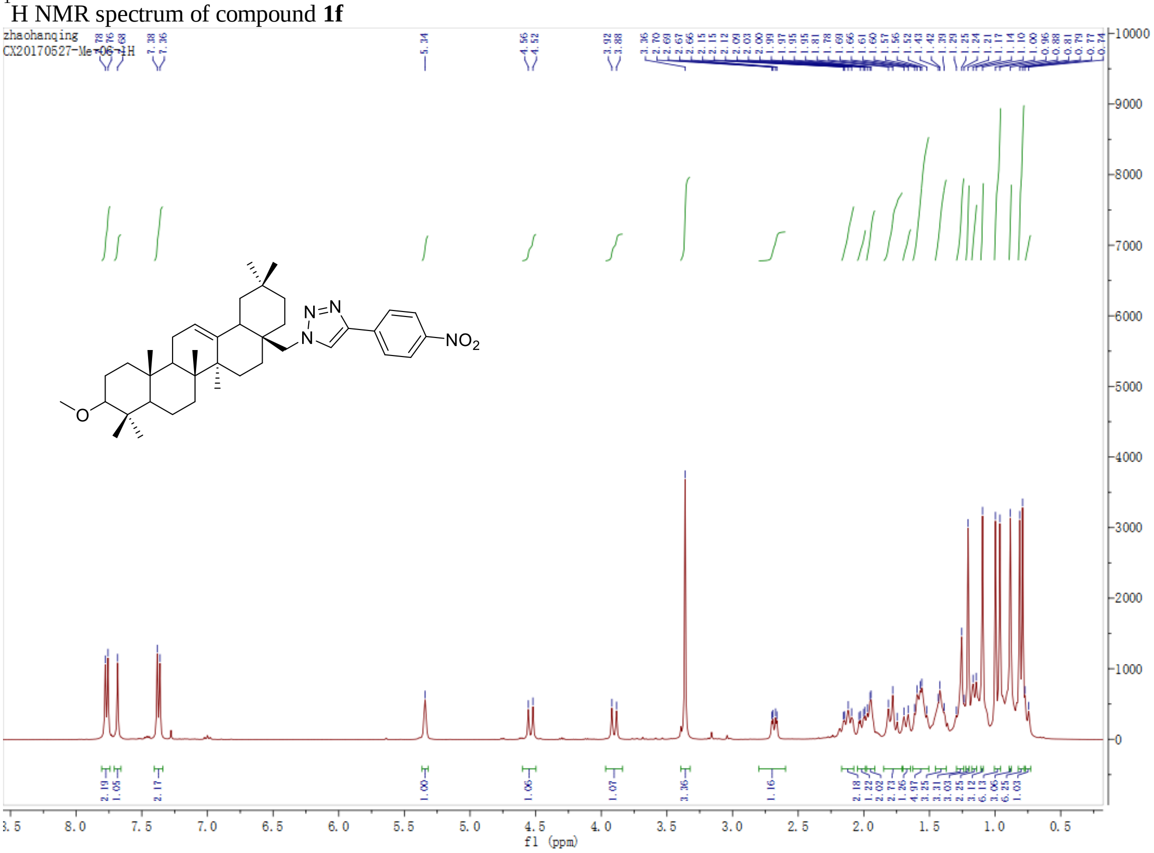

^{13}C NMR spectrum of compound **1f**

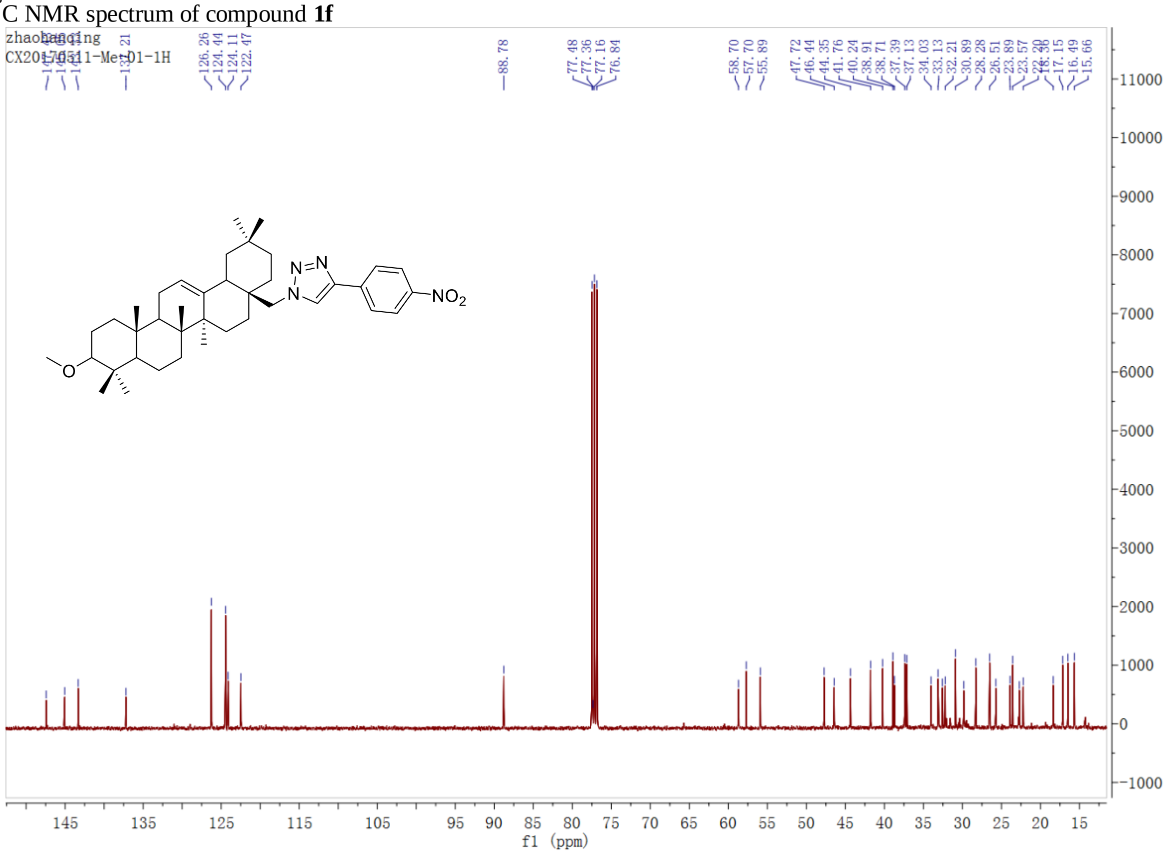

HRMS spectrum of compound **1f**

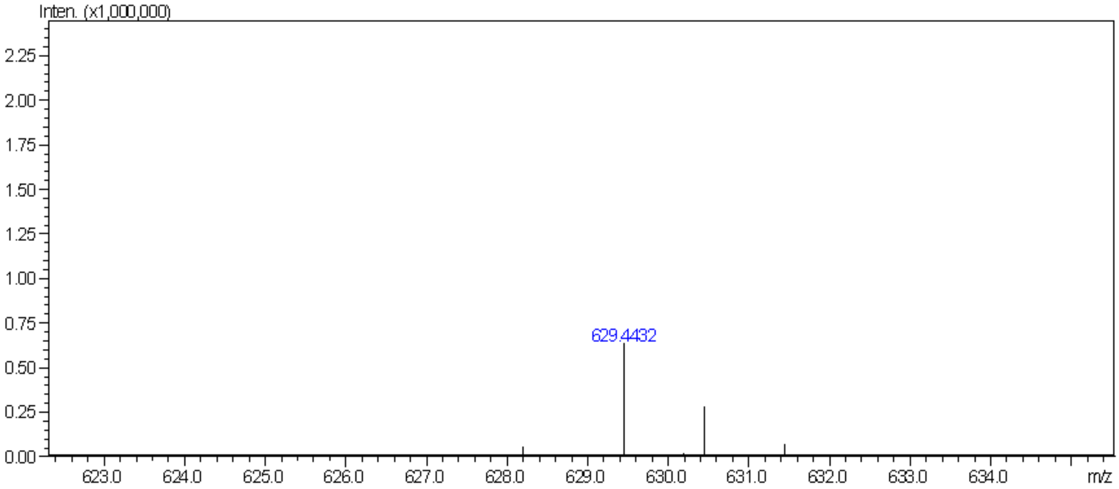

^{1}H NMR spectrum of compound **1g**

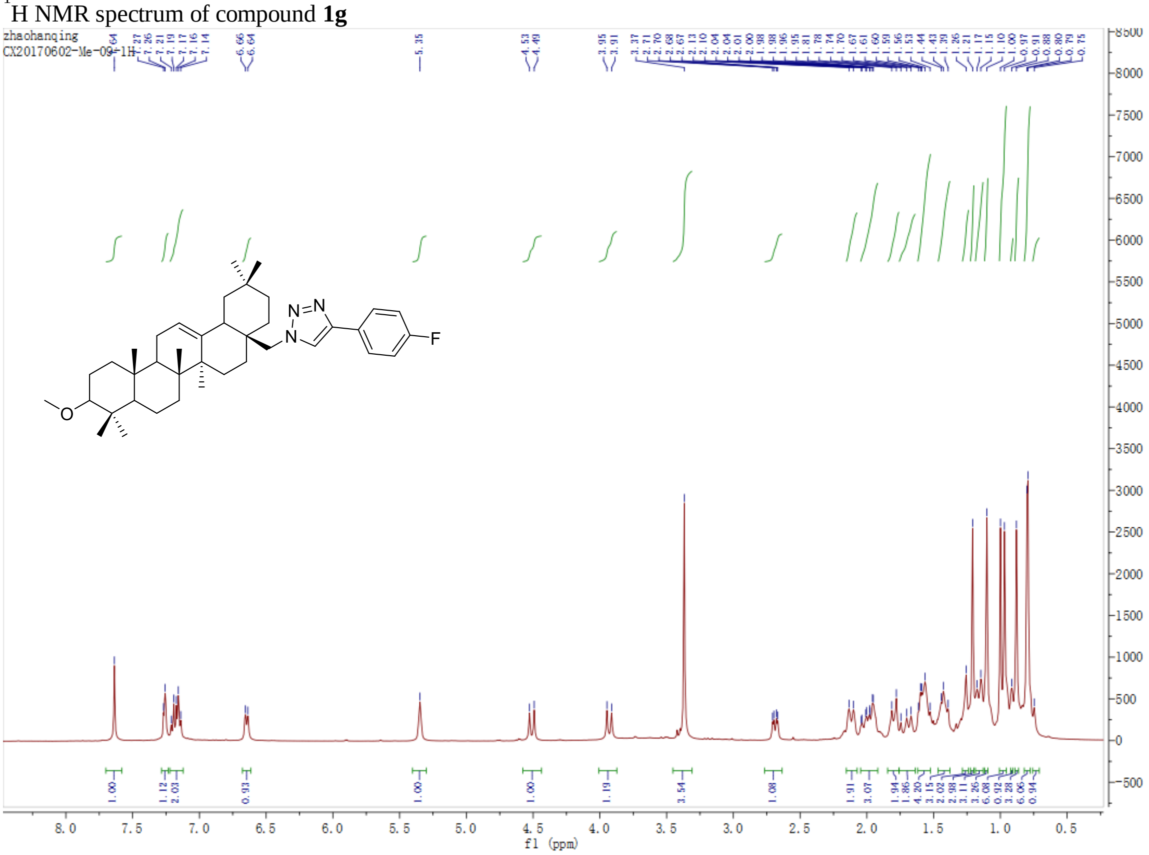

^{13}C NMR spectrum of compound **1g**

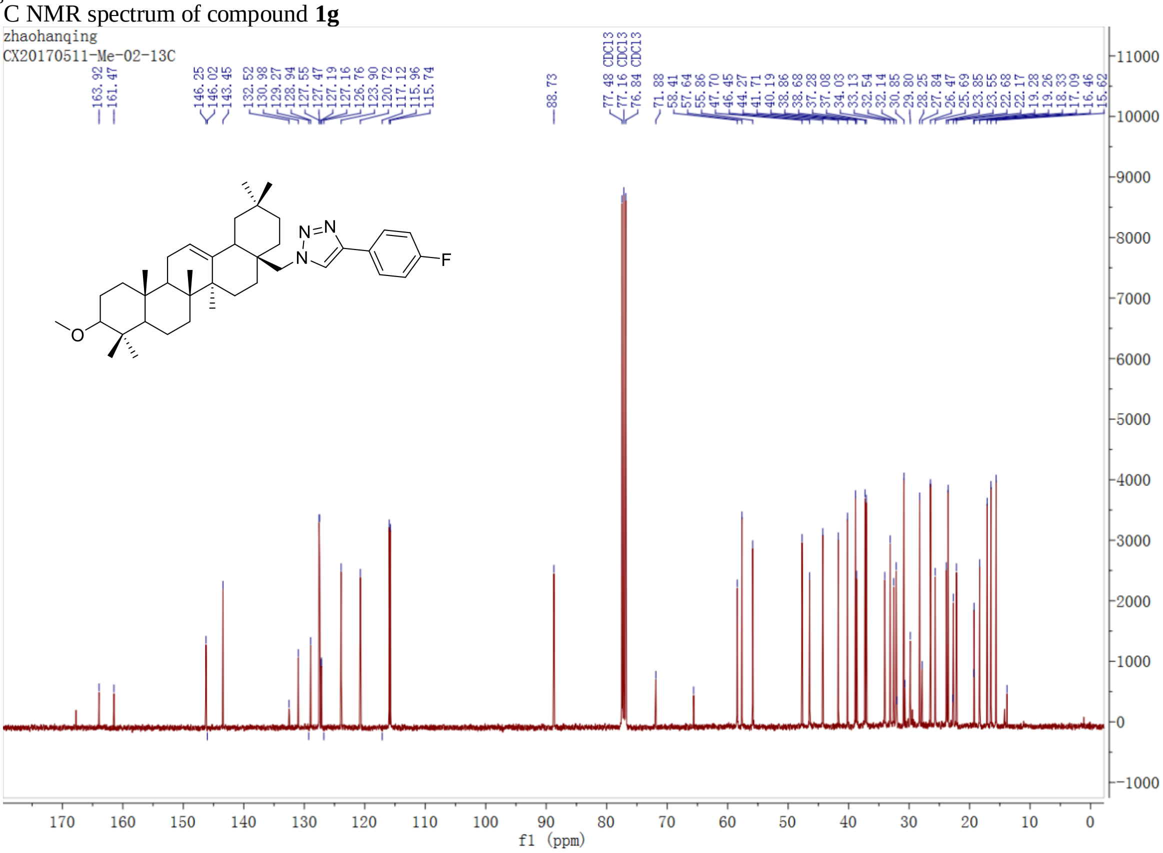

HRMS spectrum of compound **1g**

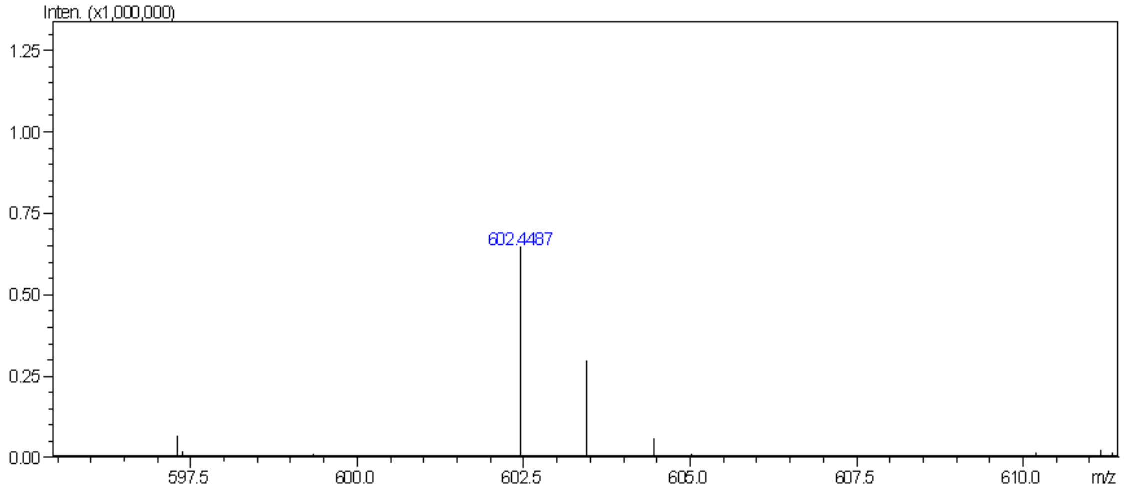

^{1}H NMR spectrum of compound **1h**

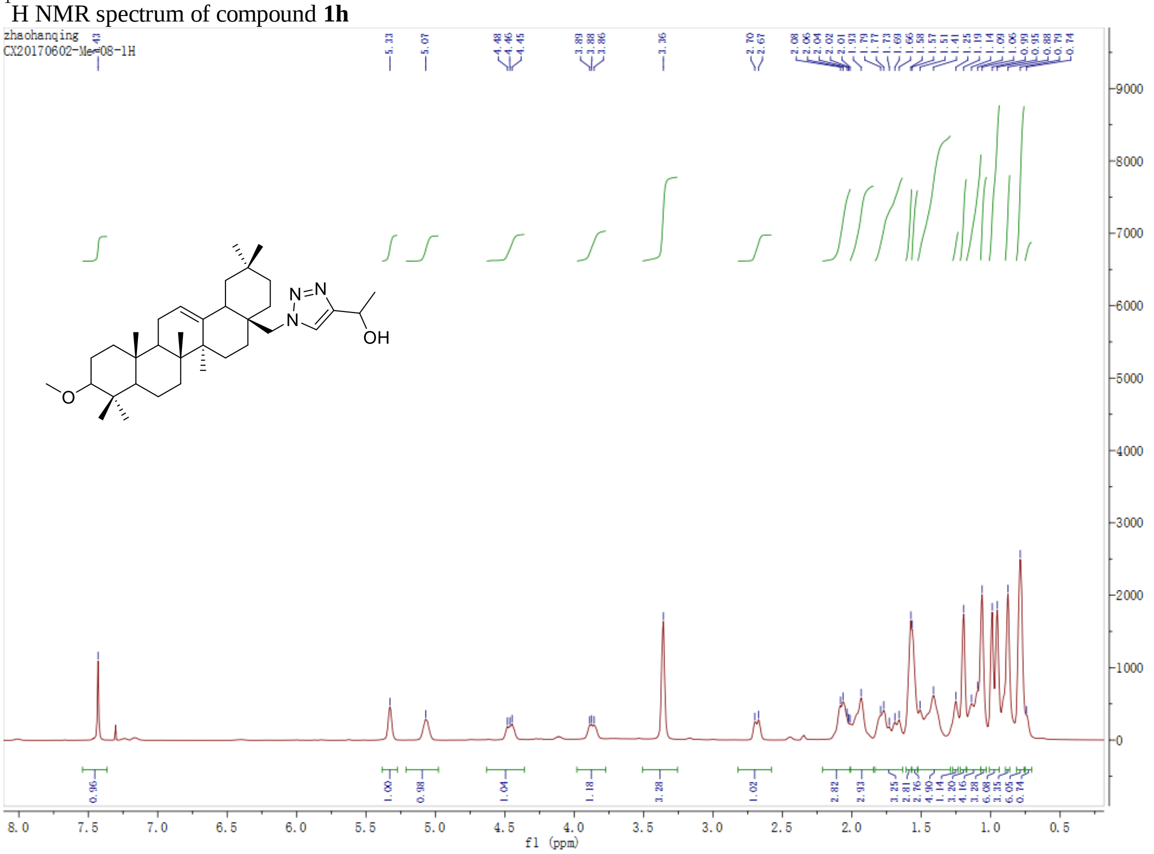

^{13}C NMR spectrum of compound **1h**

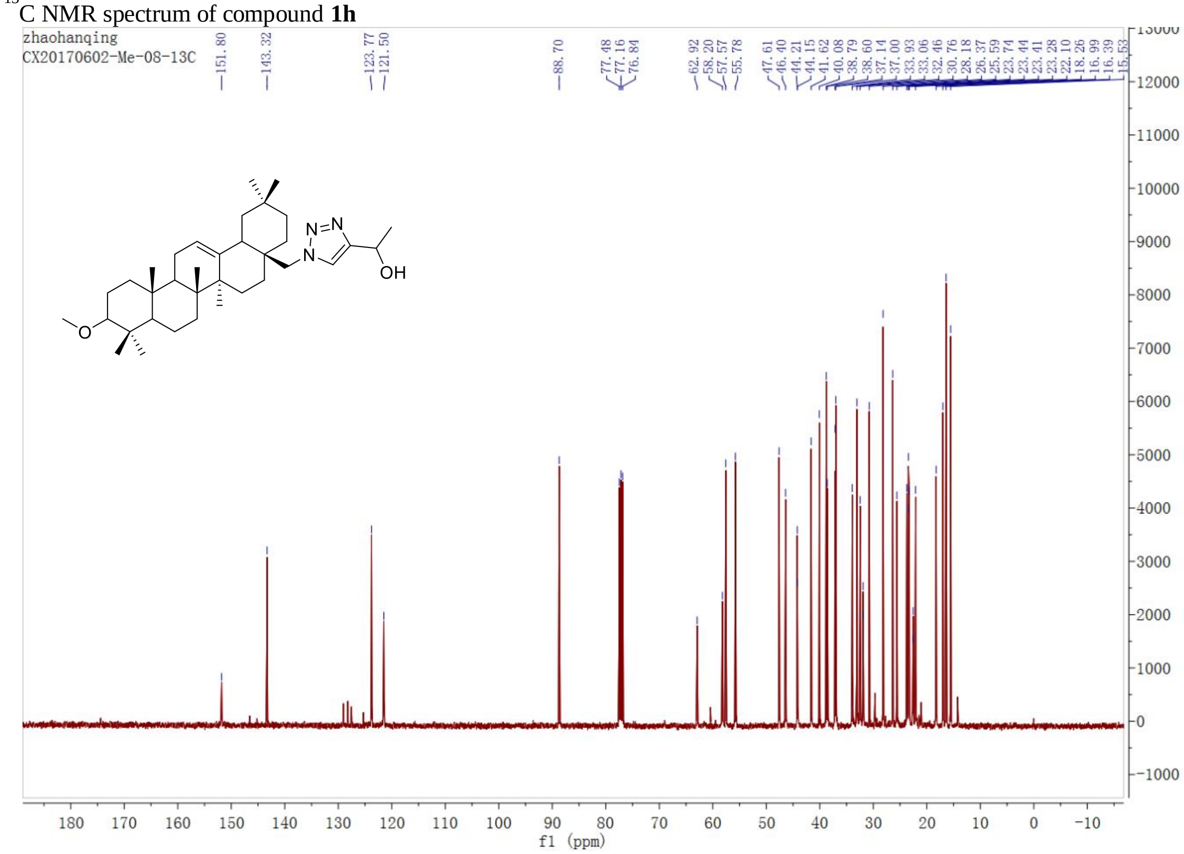

HRMS spectrum of compound **1h**

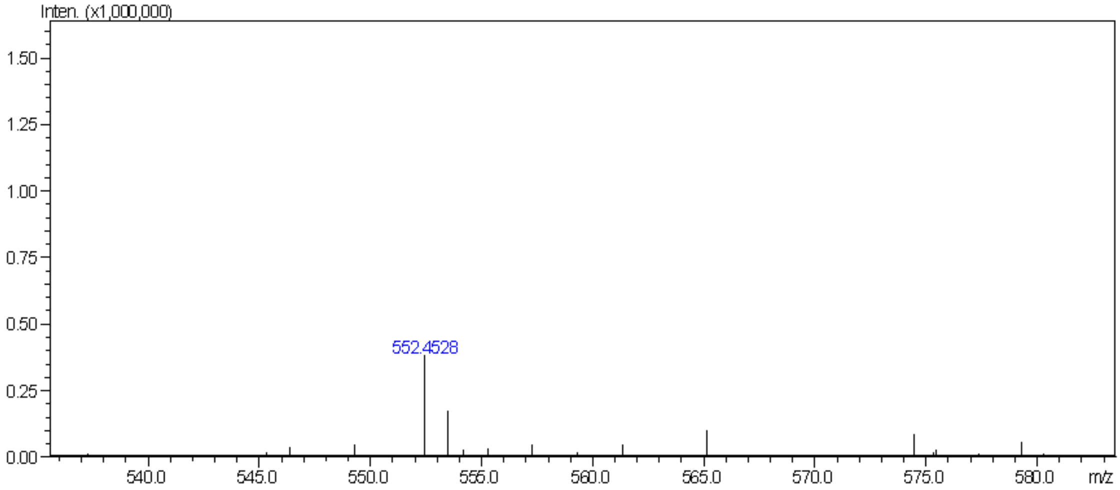

-

^{1}H NMR spectrum of compound **1i**

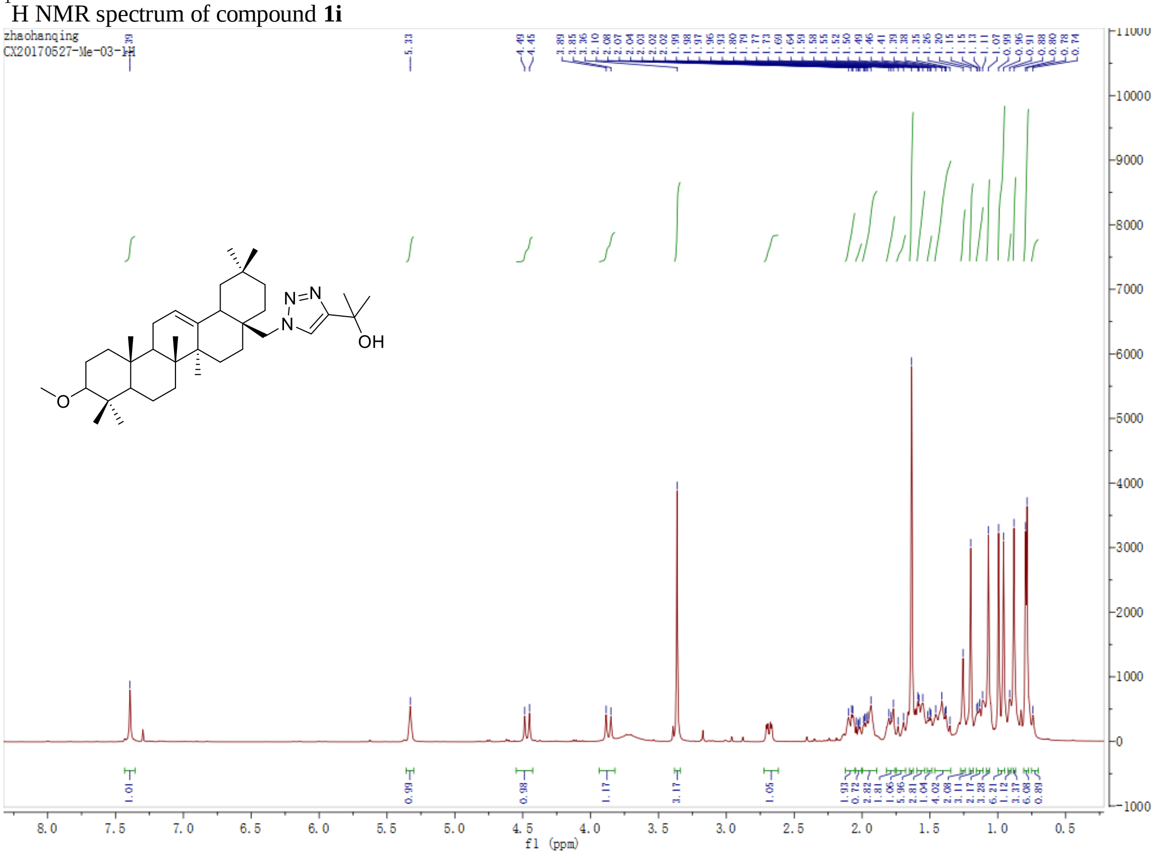

^{13}C NMR spectrum of compound **1i**

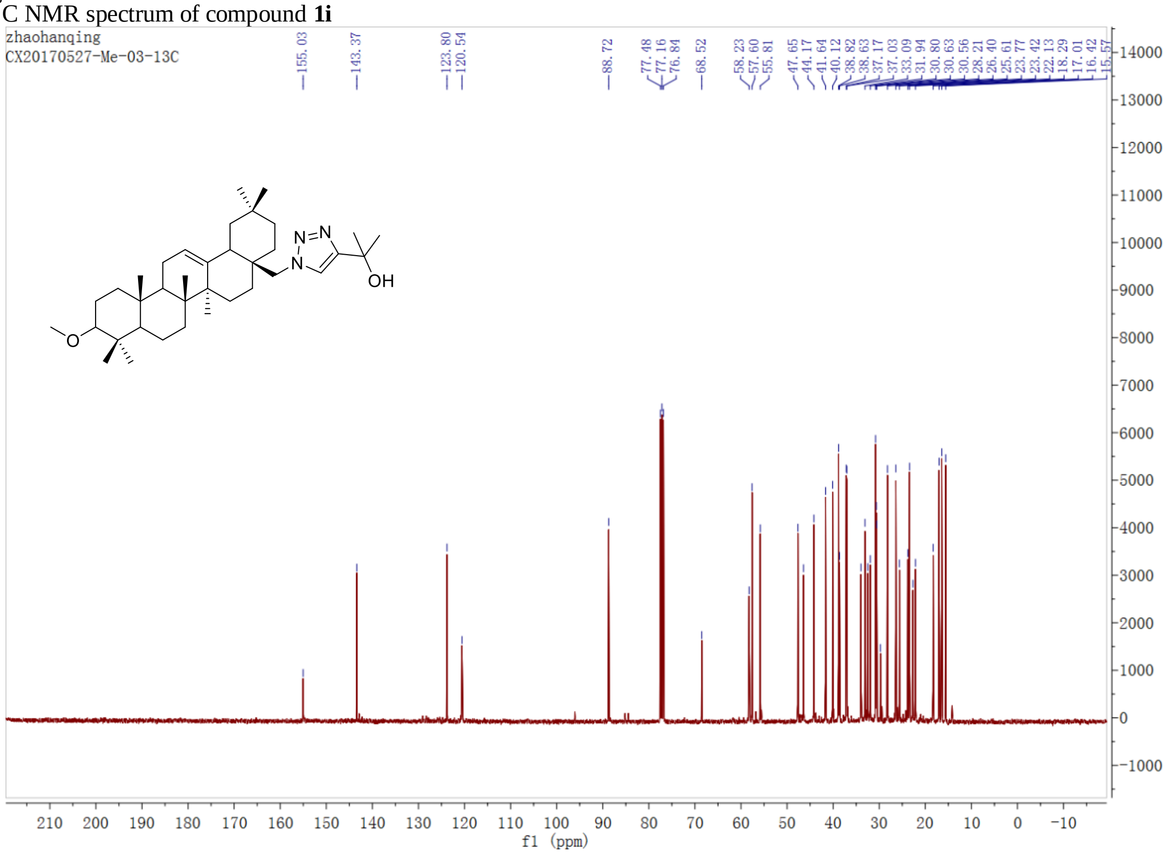

HRMS spectrum of compound **1i**

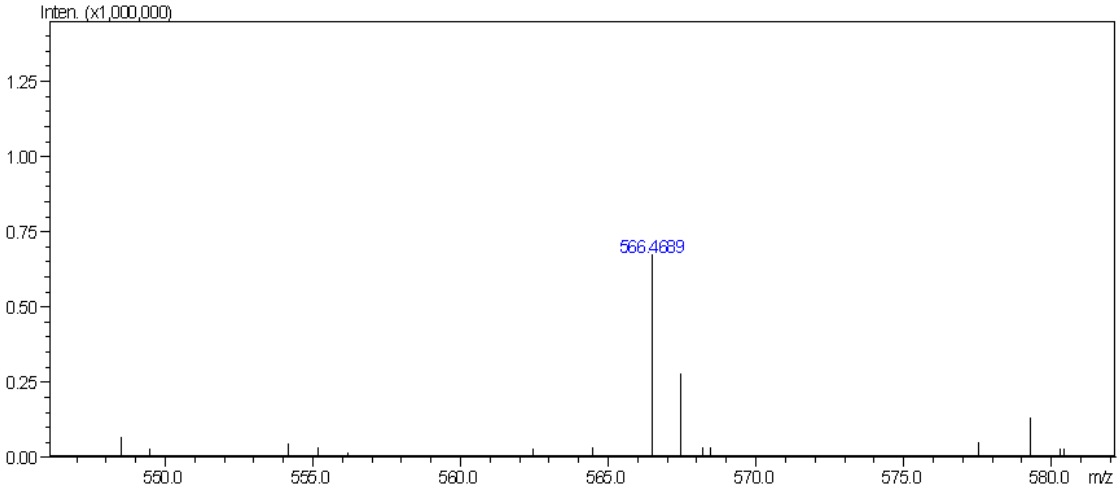

^{1}H NMR spectrum of compound **2a**

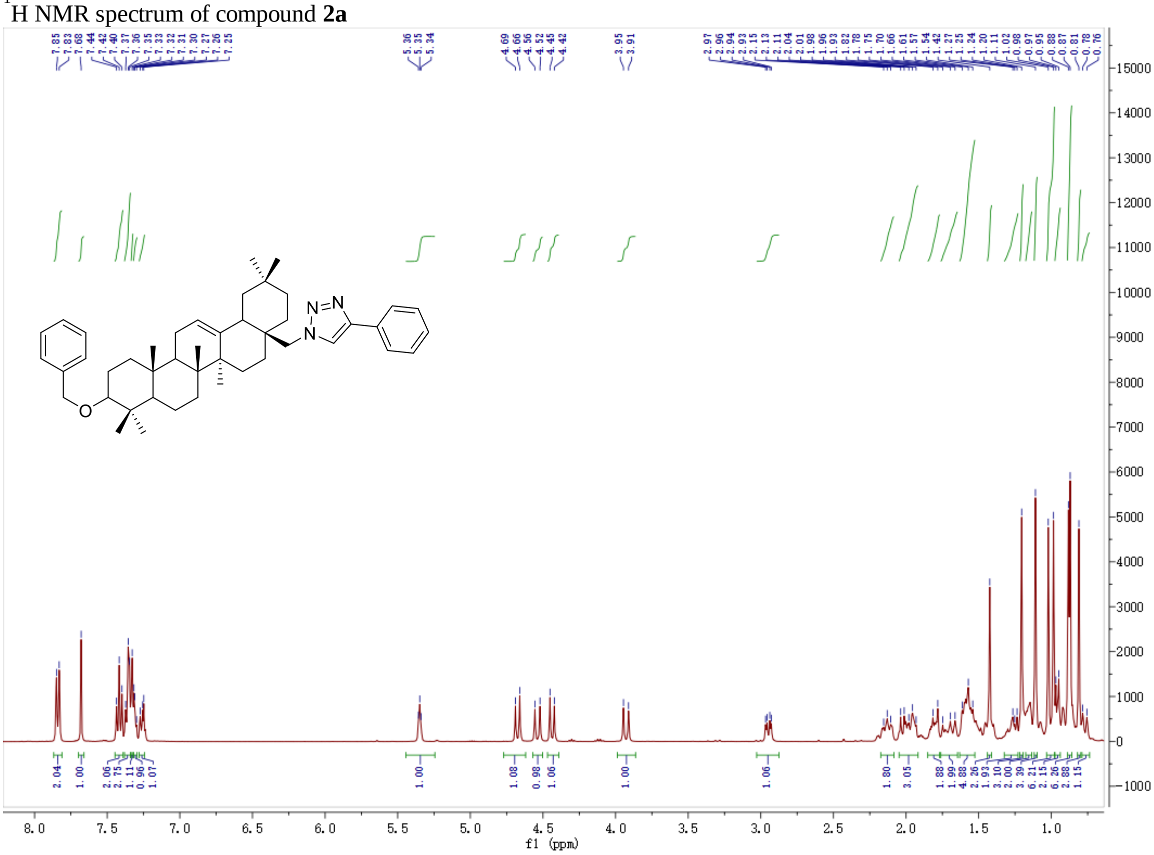

^{13}C NMR spectrum of compound **2a**

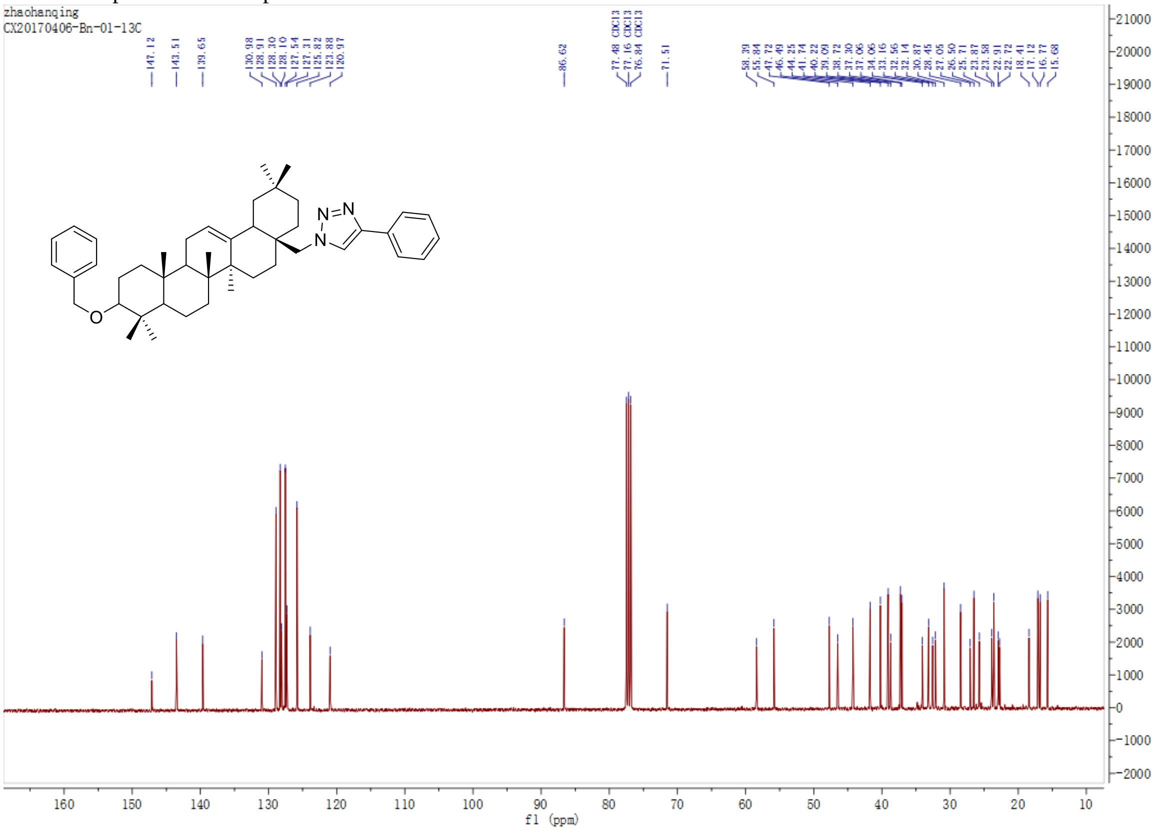

HRMS spectrum of compound **2a**

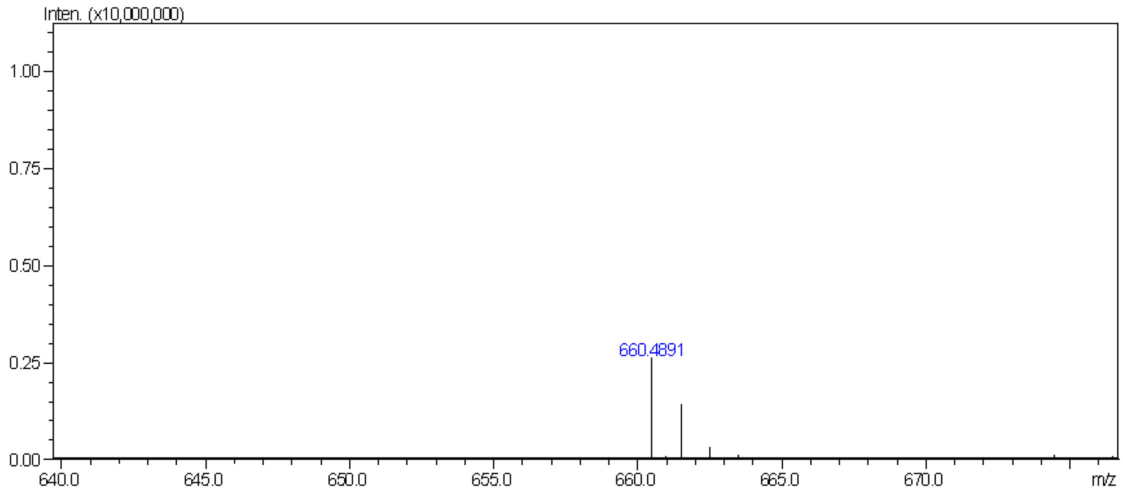

^{1}H NMR spectrum of compound **2b**

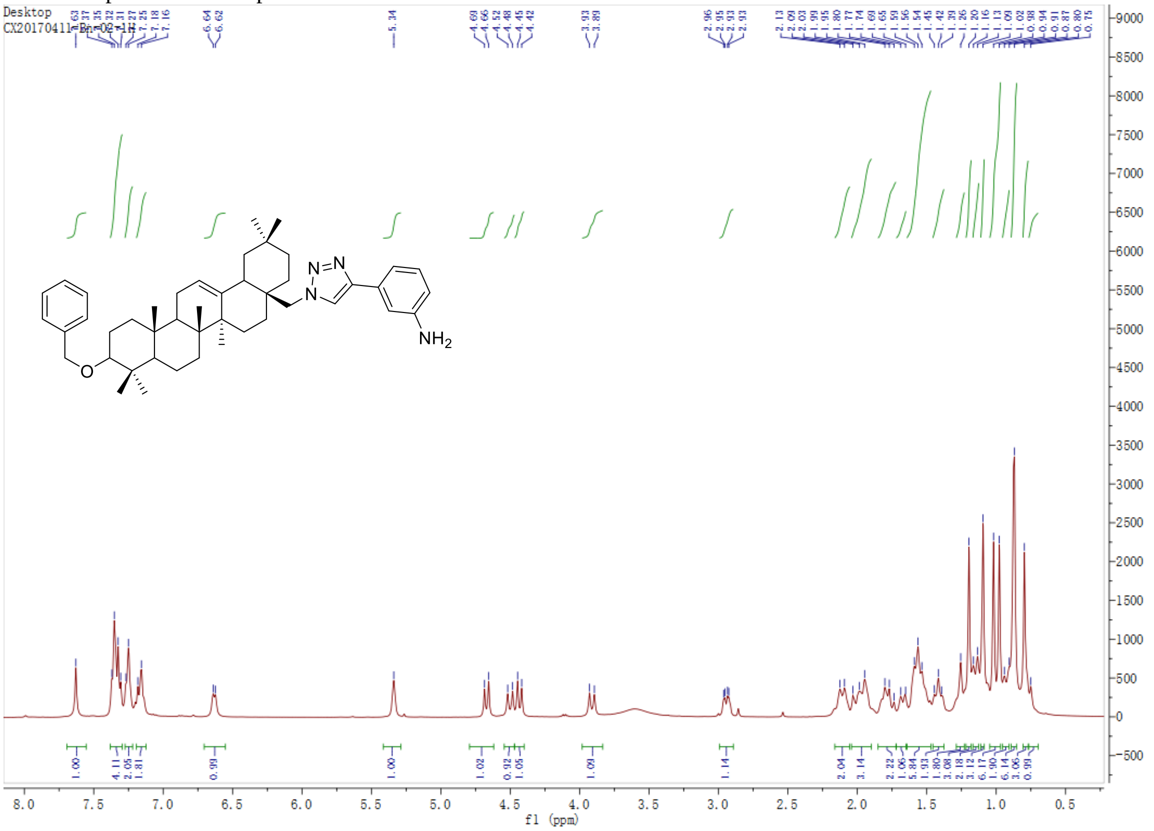

^{13}C NMR spectrum of compound **2b**

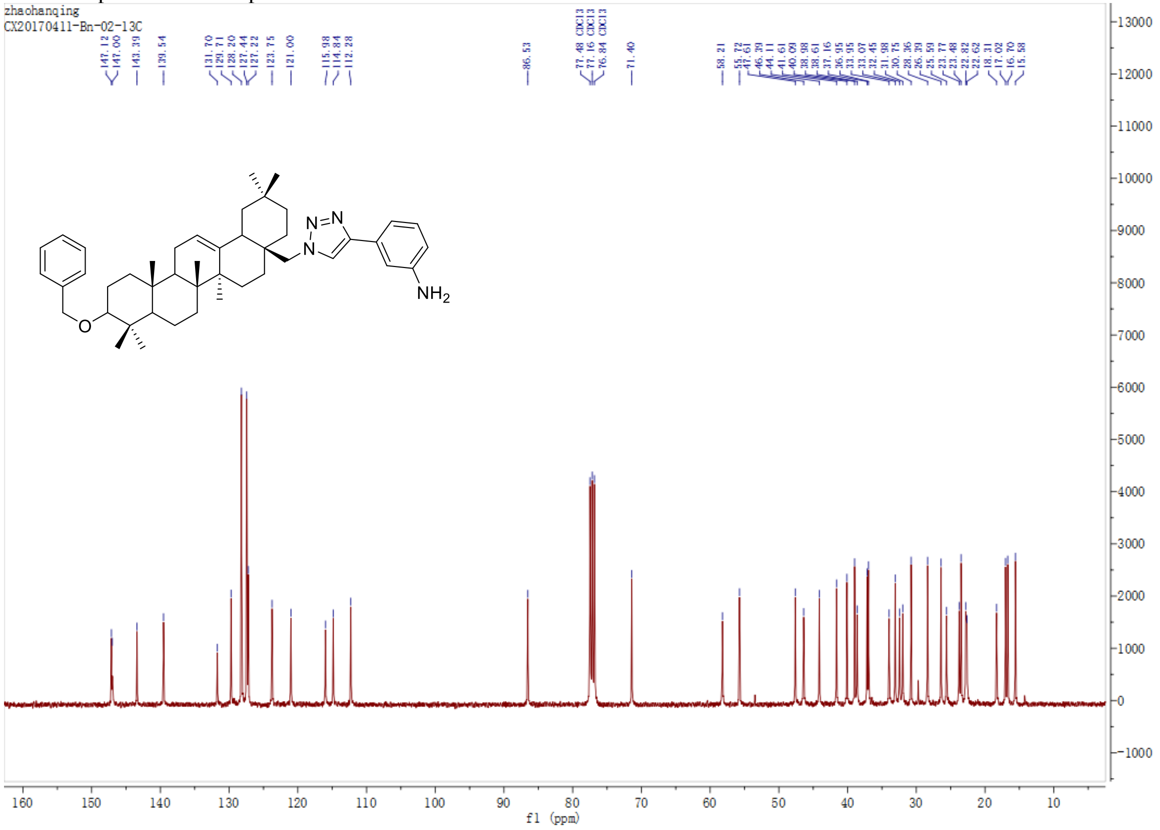

HRMS spectrum of compound **2b**

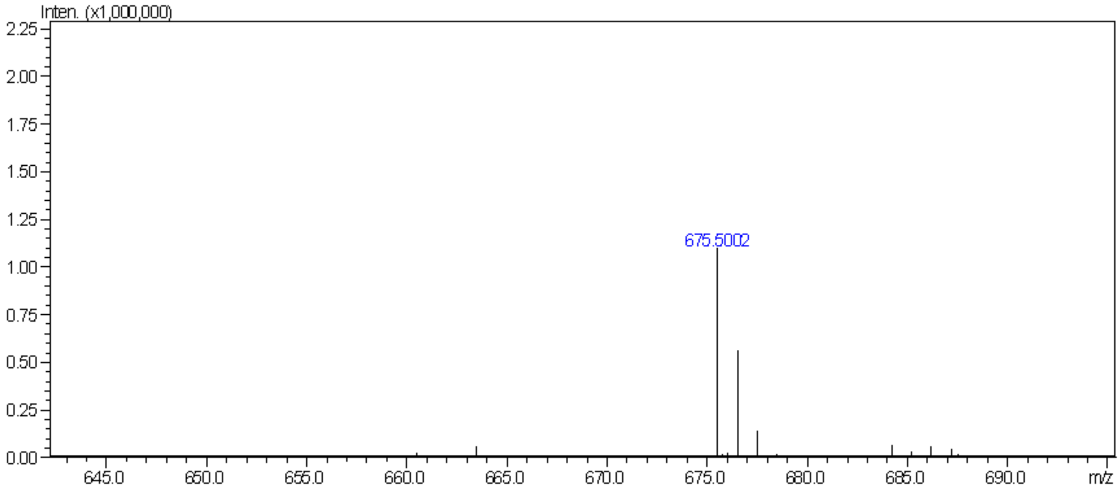

^{1}H NMR spectrum of compound **2c**

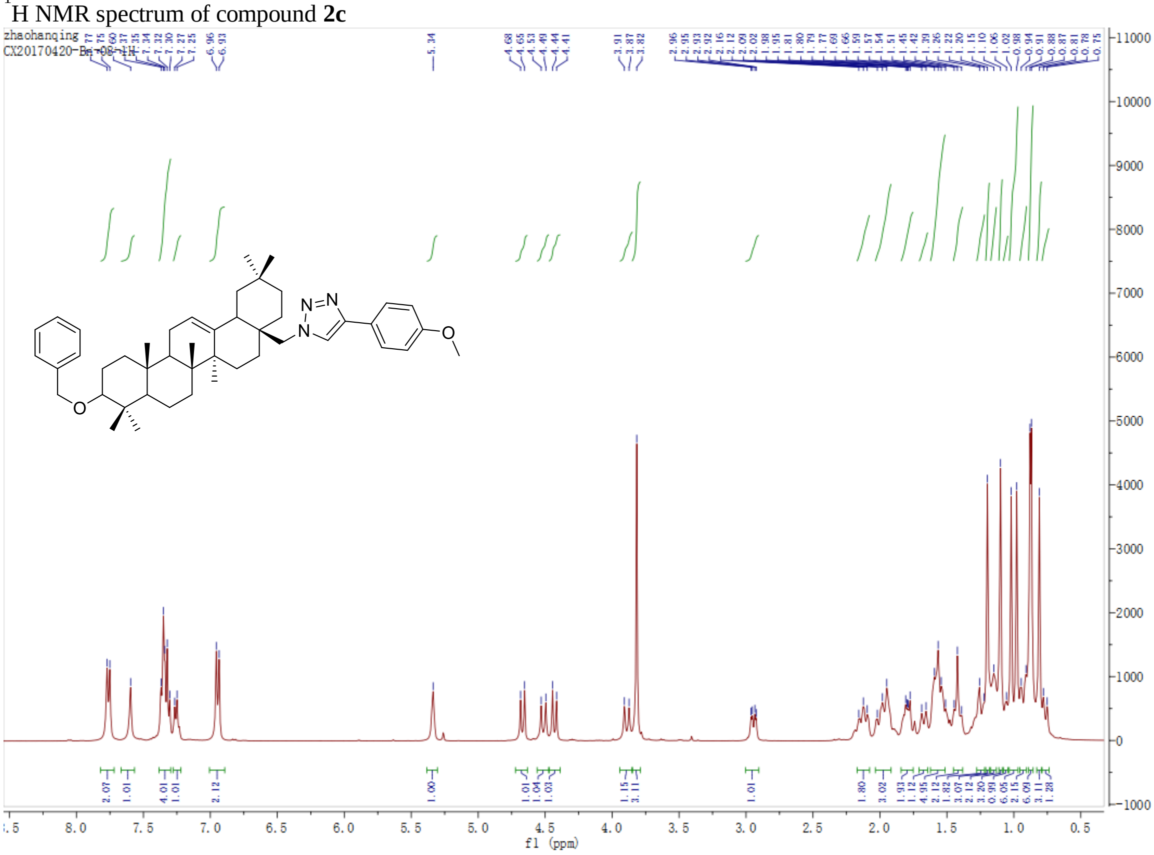

^{13}C NMR spectrum of compound **2c**

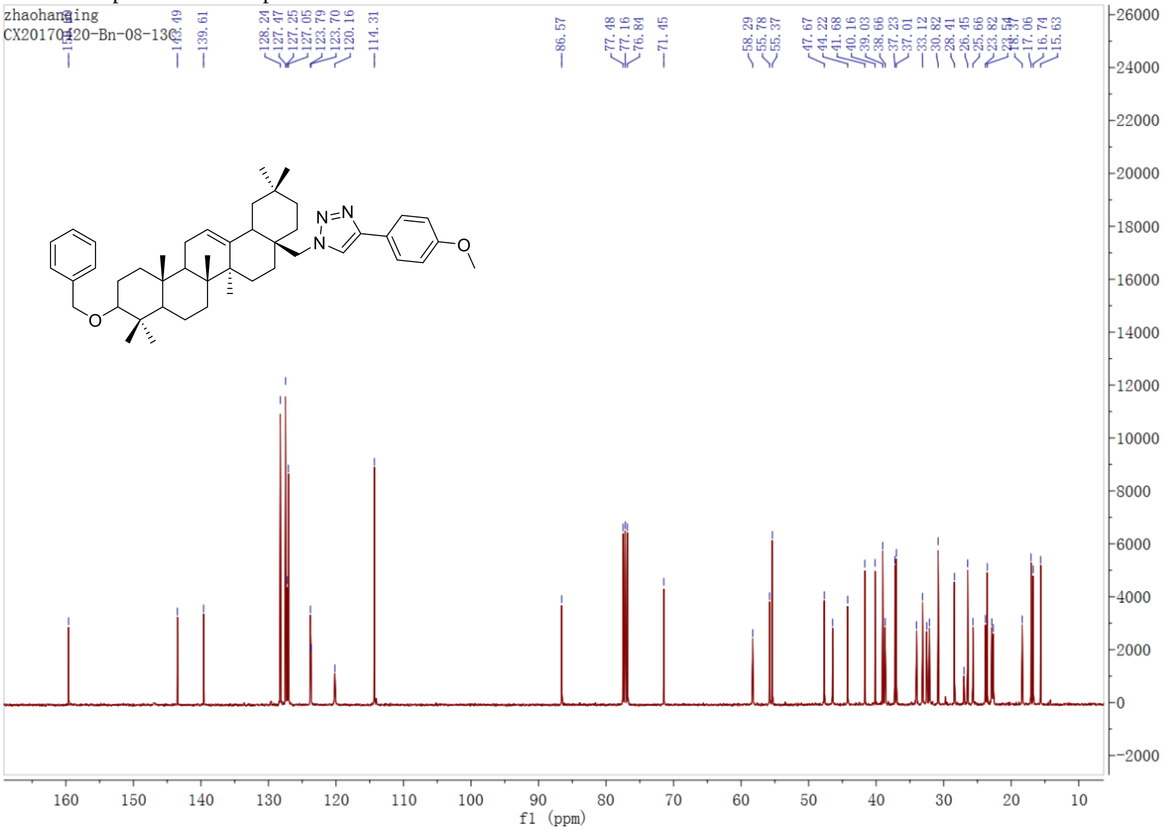

HRMS spectrum of compound **2c**

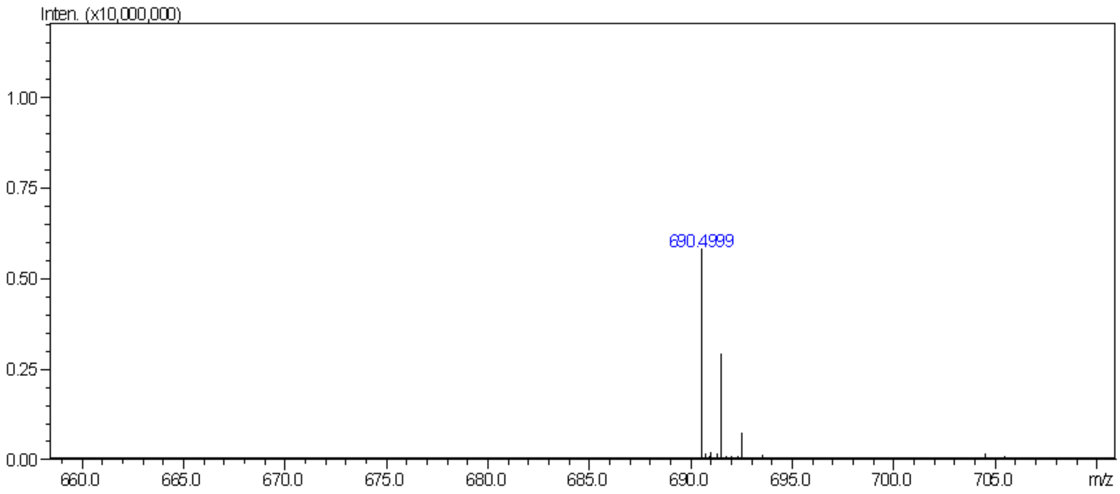

^{1}H NMR spectrum of compound **2d**

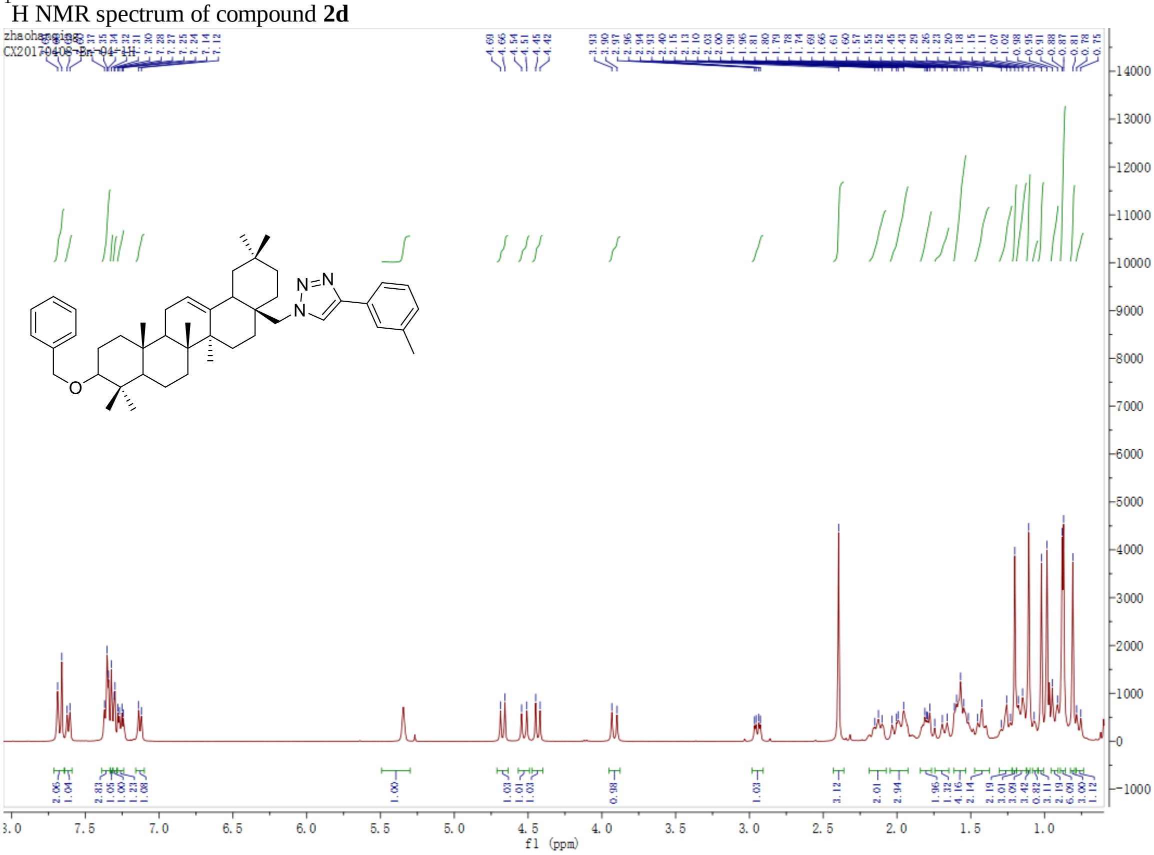

^{13}C NMR spectrum of compound **2d**

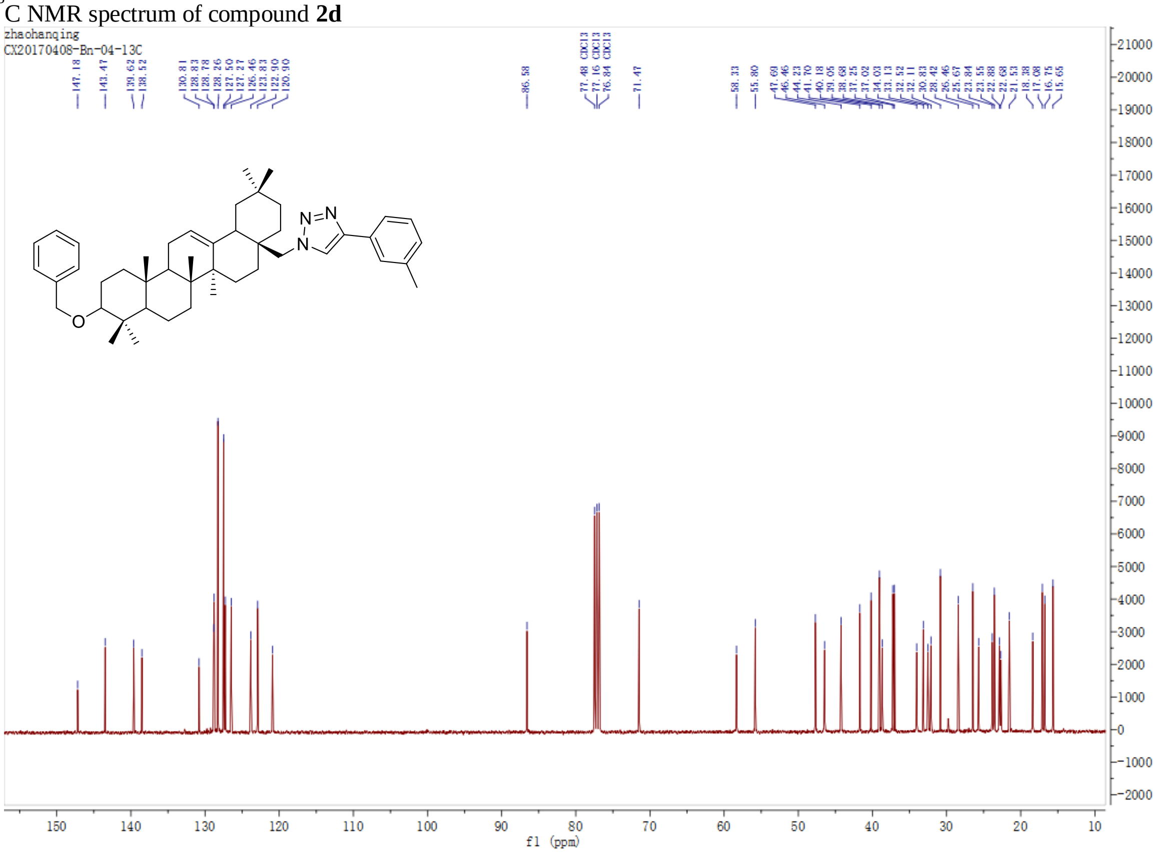

HRMS spectrum of compound **2d**

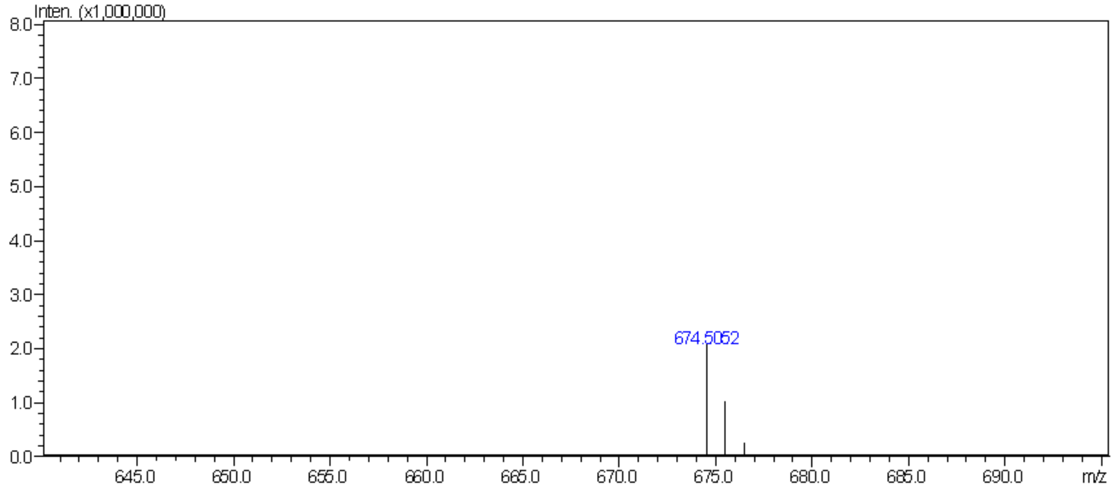

^{1}H NMR spectrum of compound **2e**

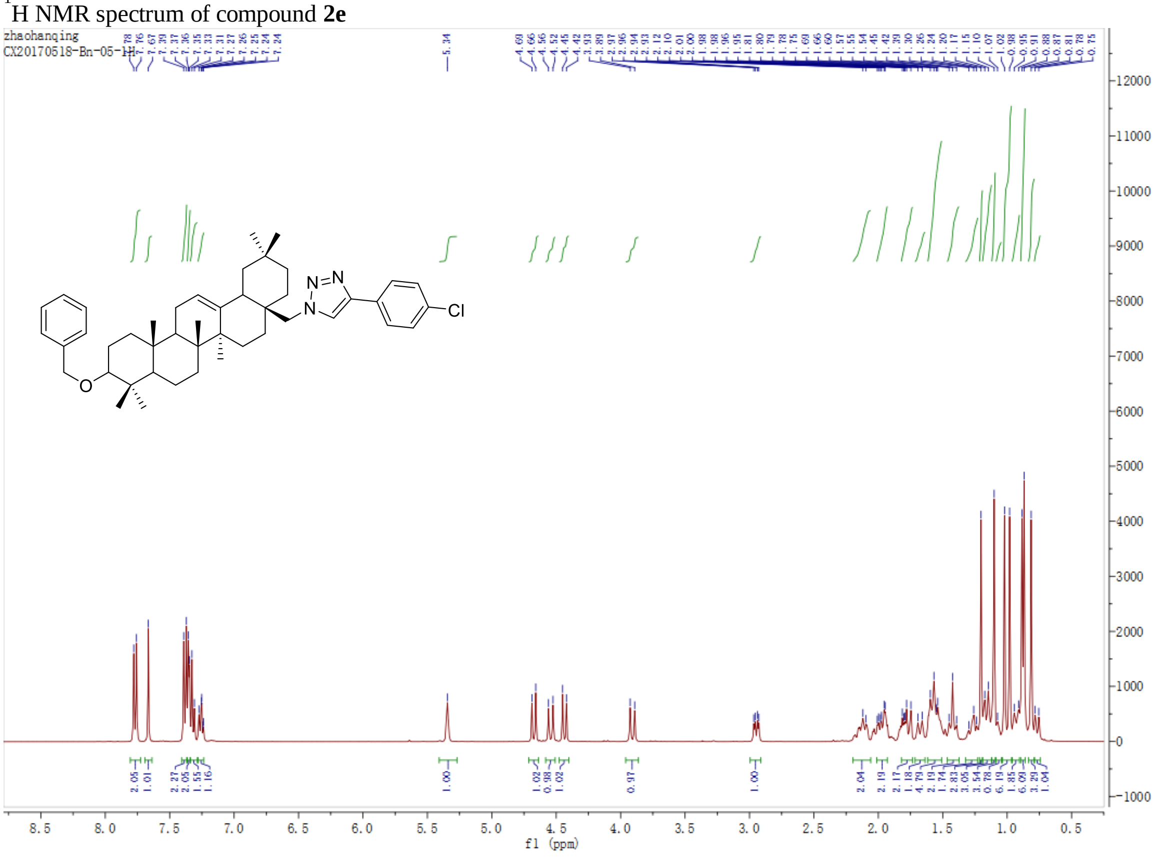

^{13}C NMR spectrum of compound **2e**

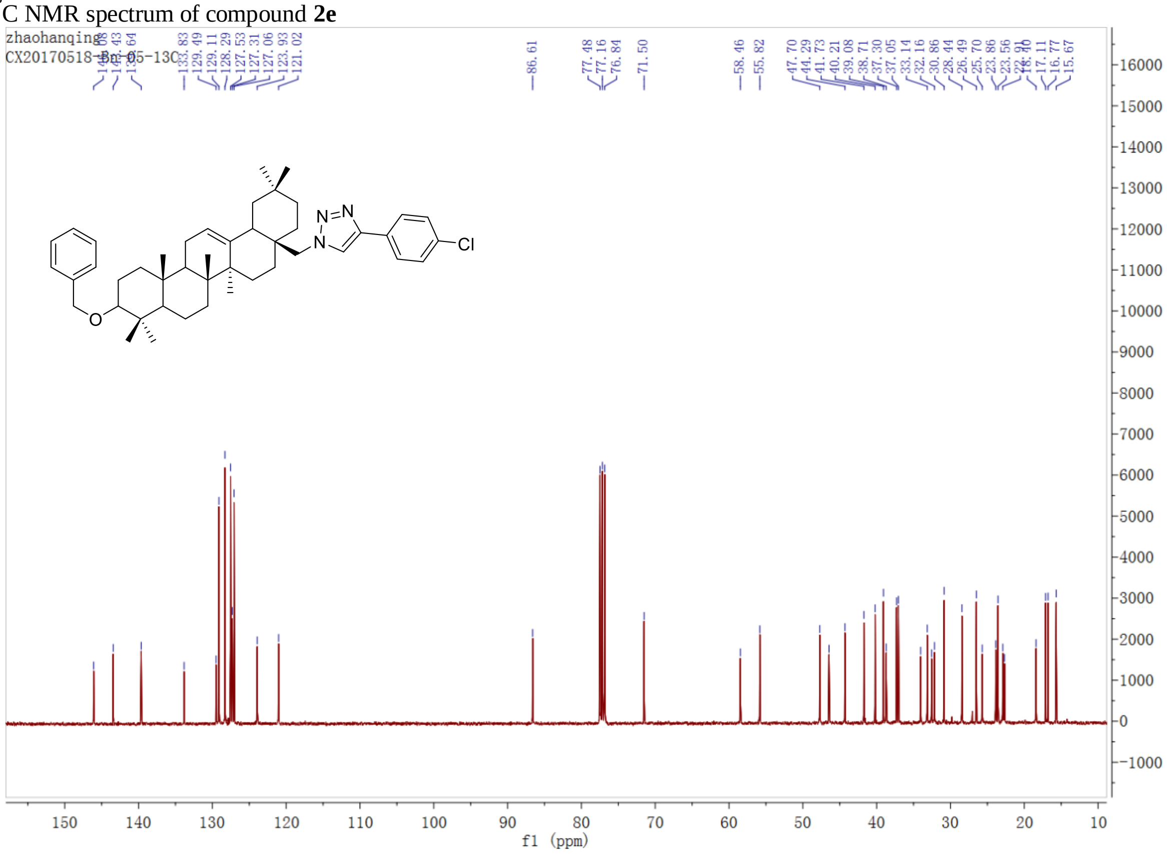

HRMS spectrum of compound **2e**

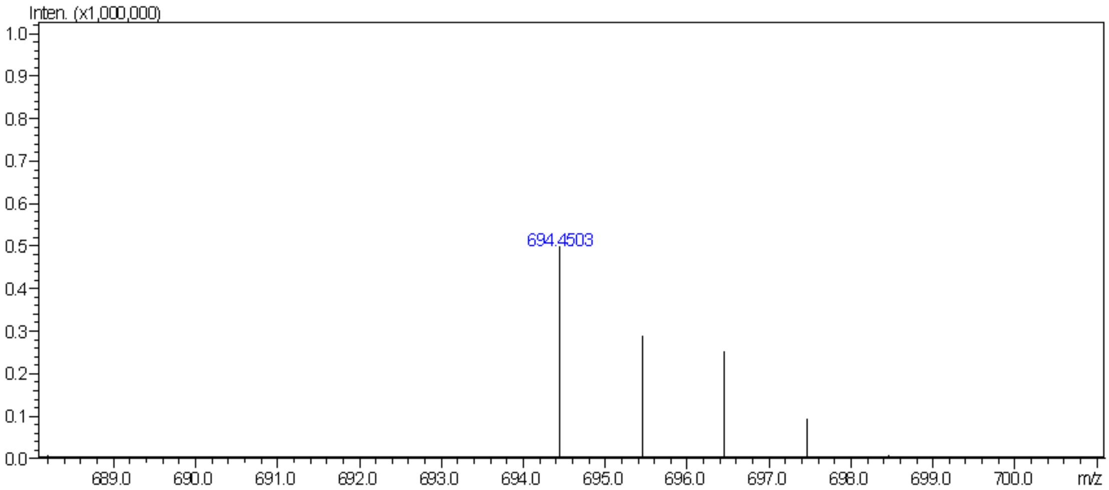

^{1}H NMR spectrum of compound **2f**

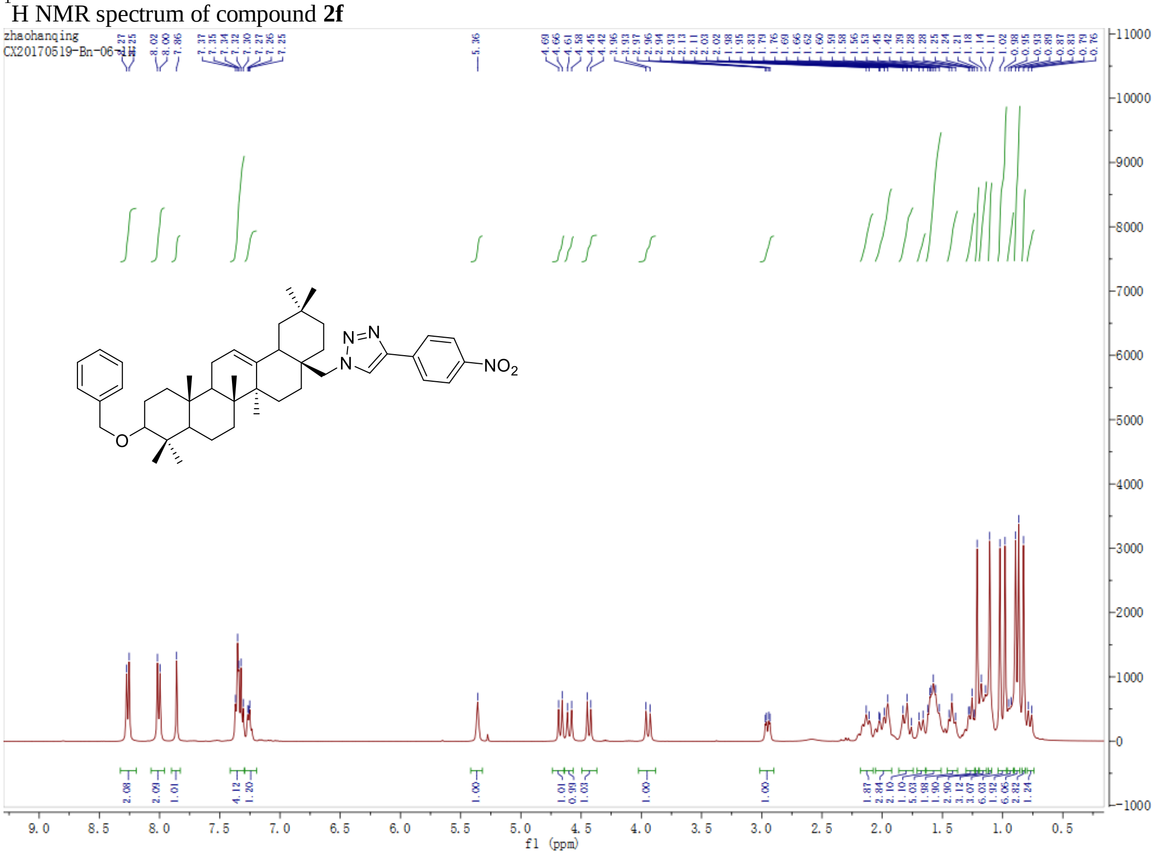

^{13}C NMR spectrum of compound **2f**

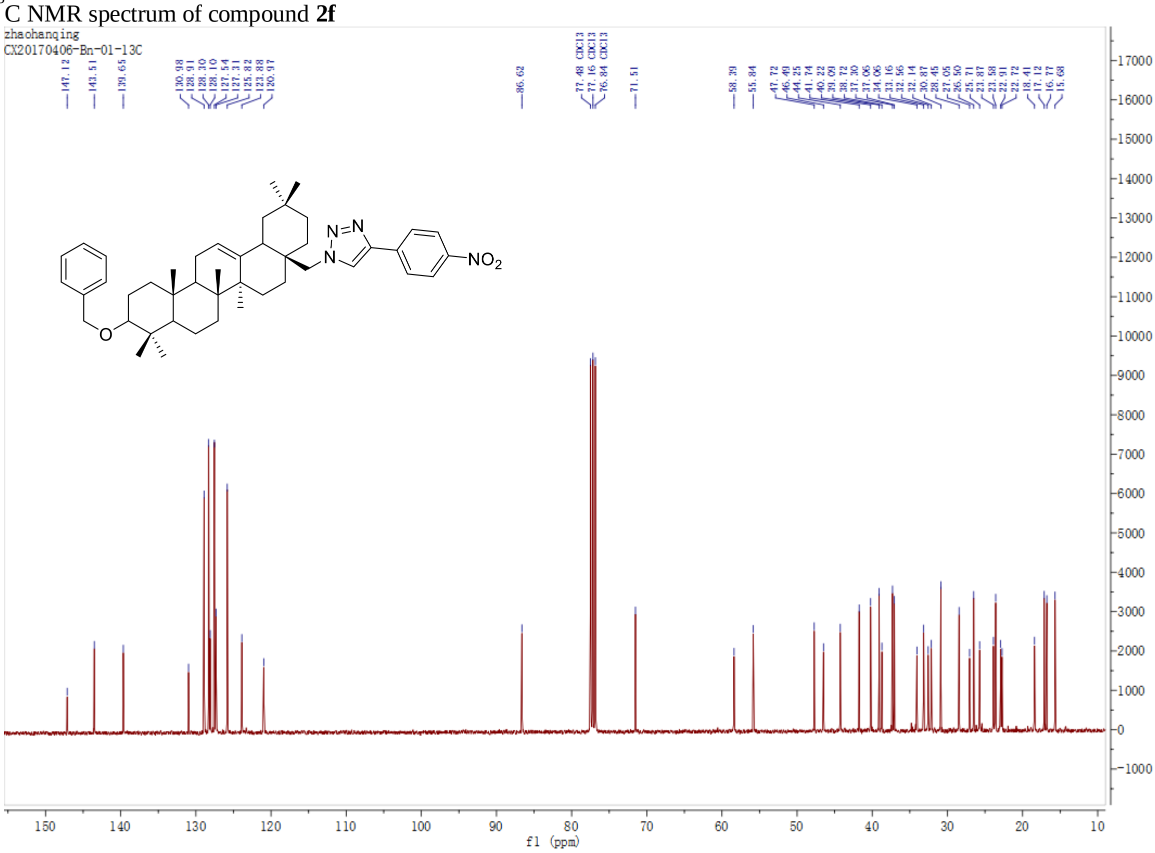

HRMS spectrum of compound **2f**

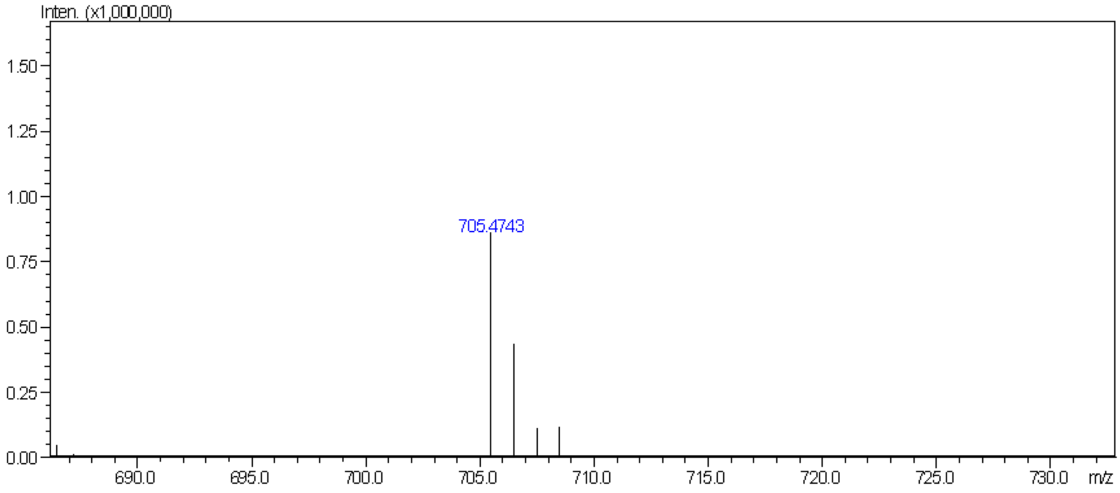

^{1}H NMR spectrum of compound **2g**

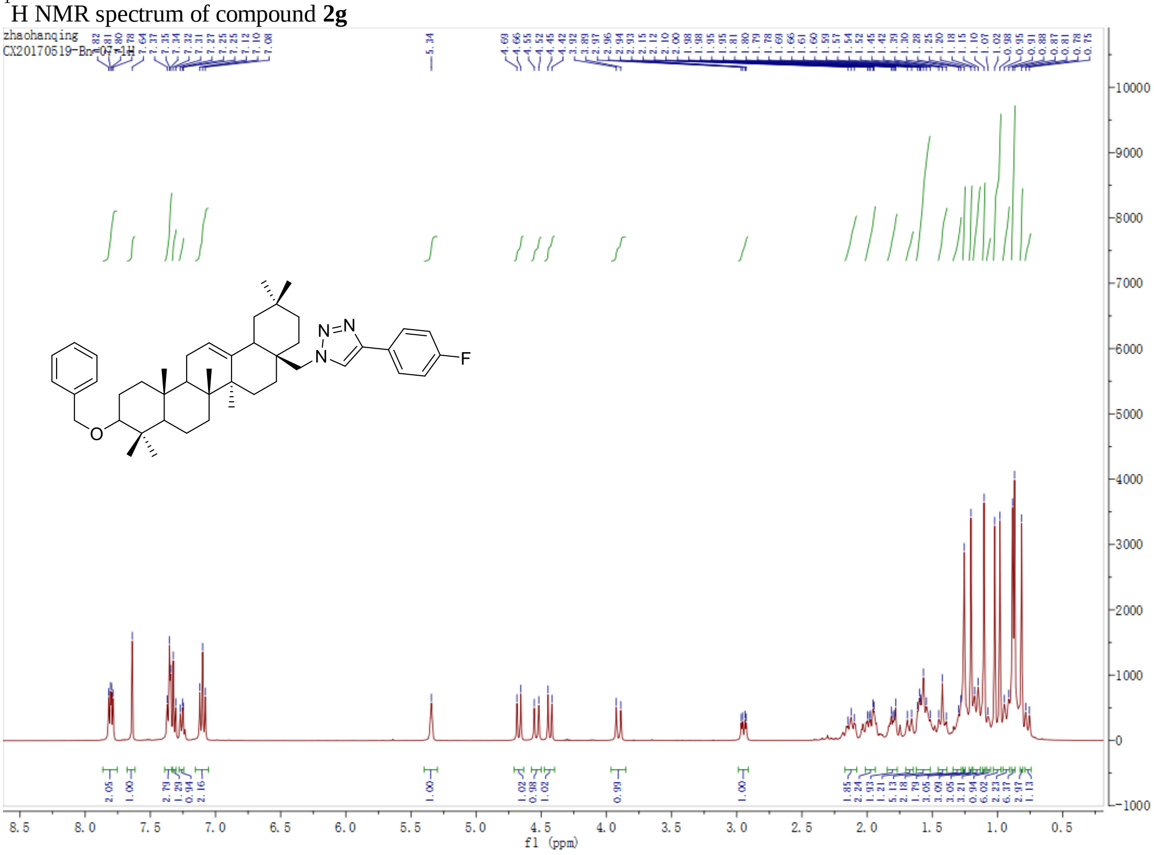

^{13}C NMR spectrum of compound **2g**

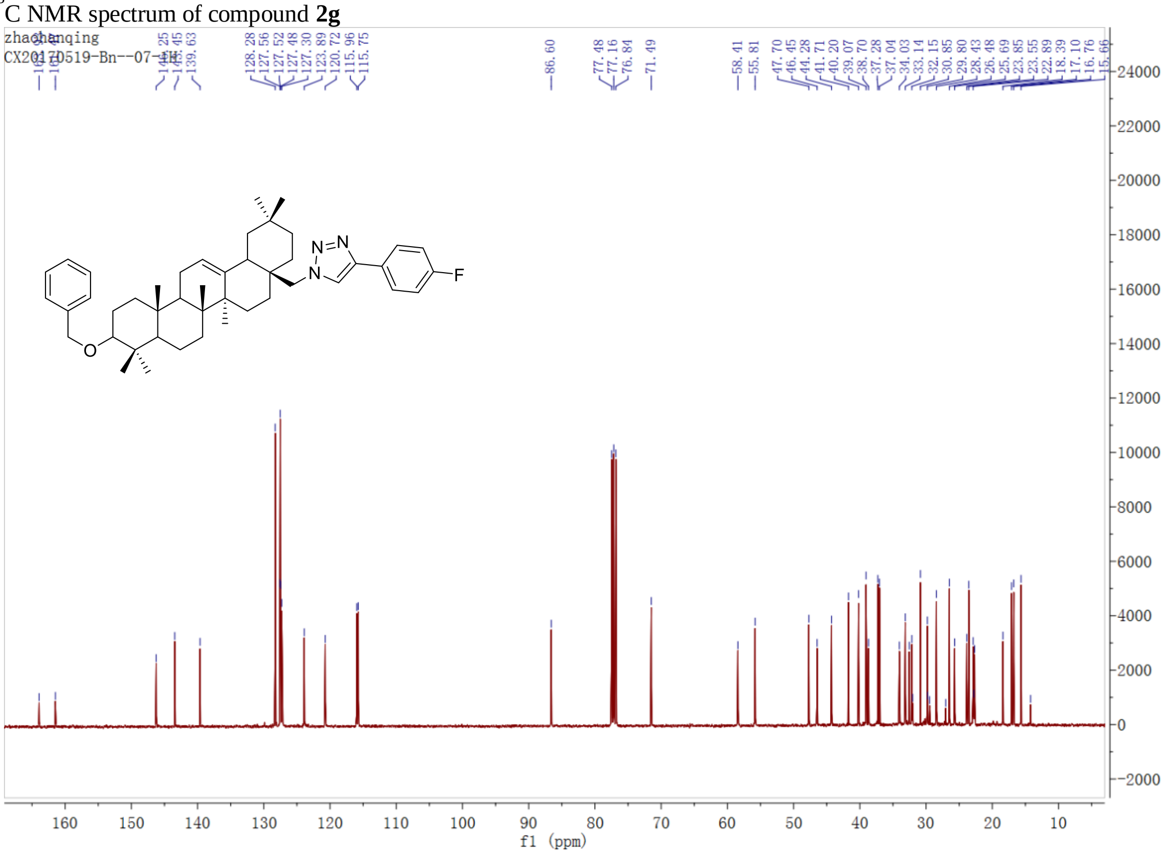

HRMS spectrum of compound **2g**

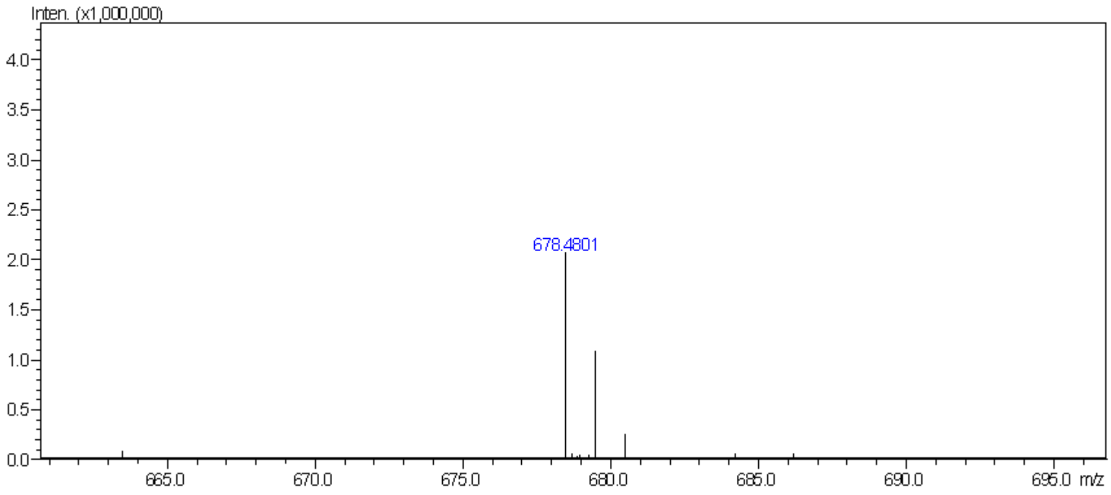

^{1}H NMR spectrum of compound **2h**

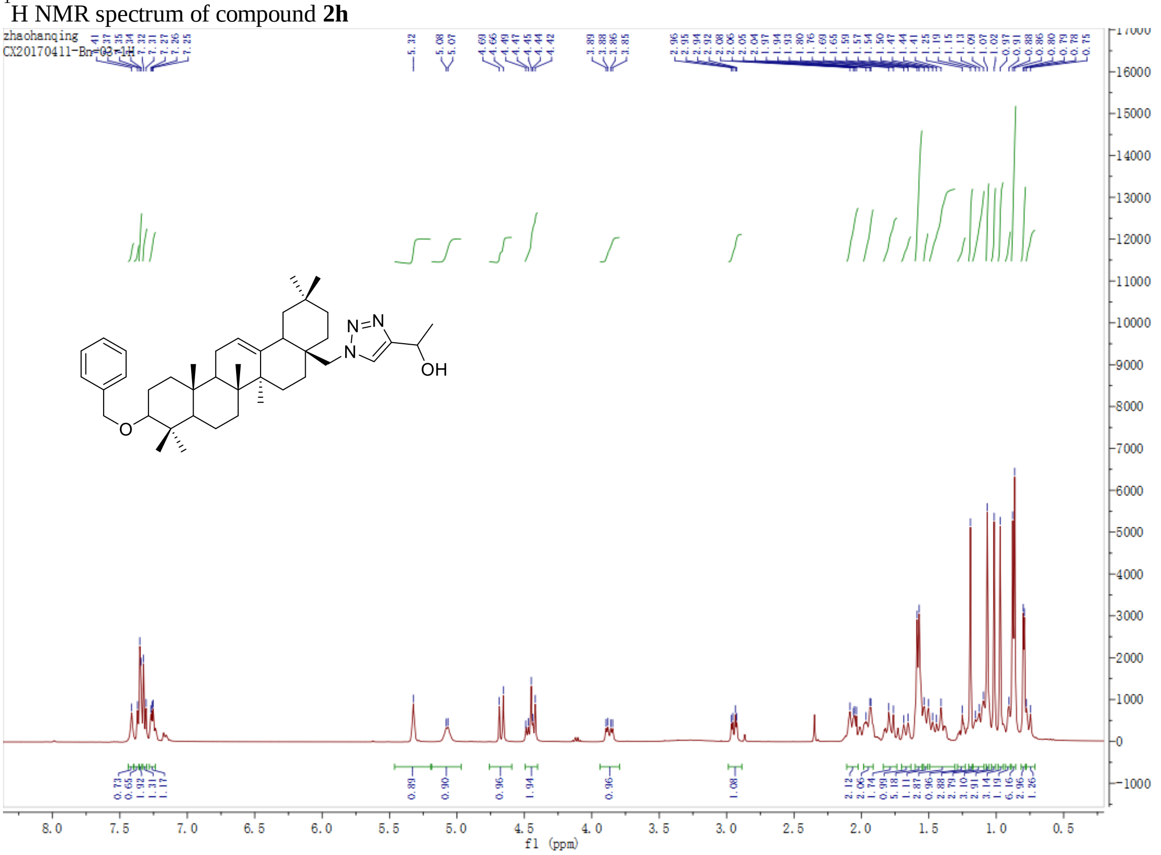

^{13}C NMR spectrum of compound **2h**

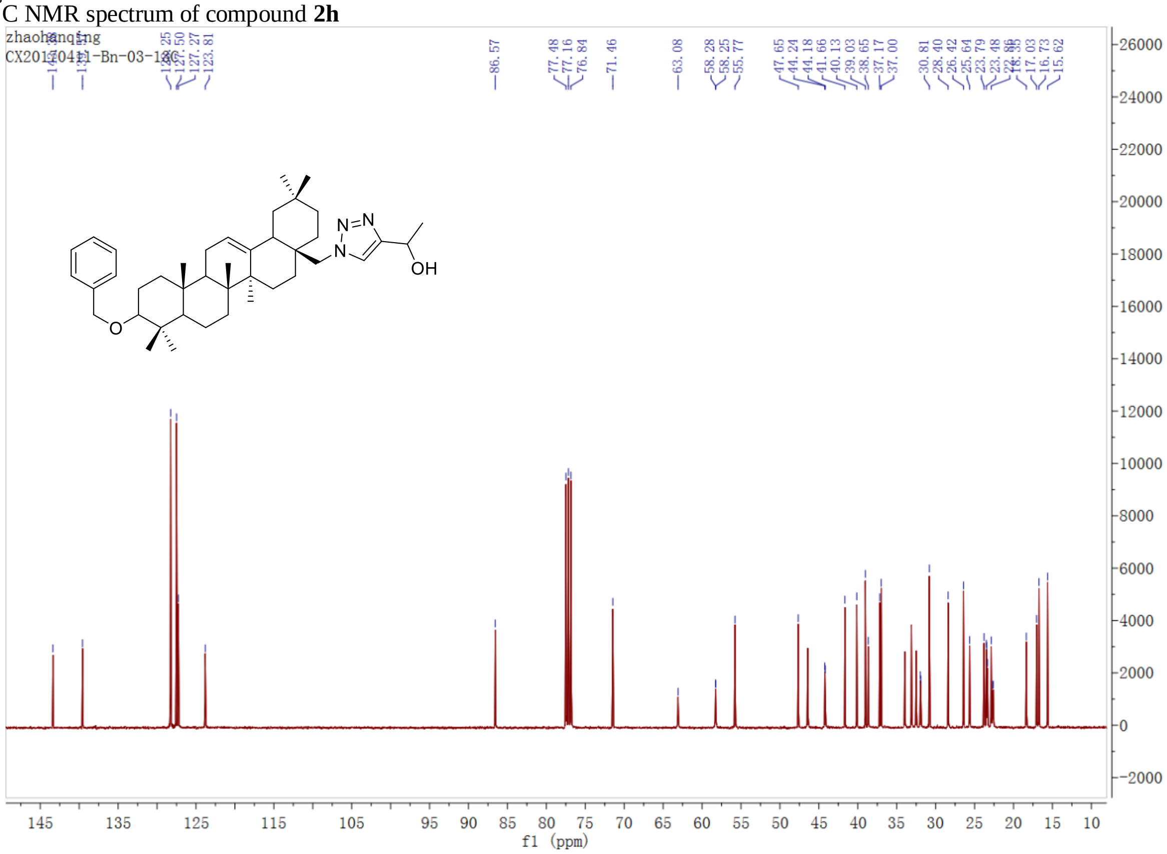

HRMS spectrum of compound **2h**

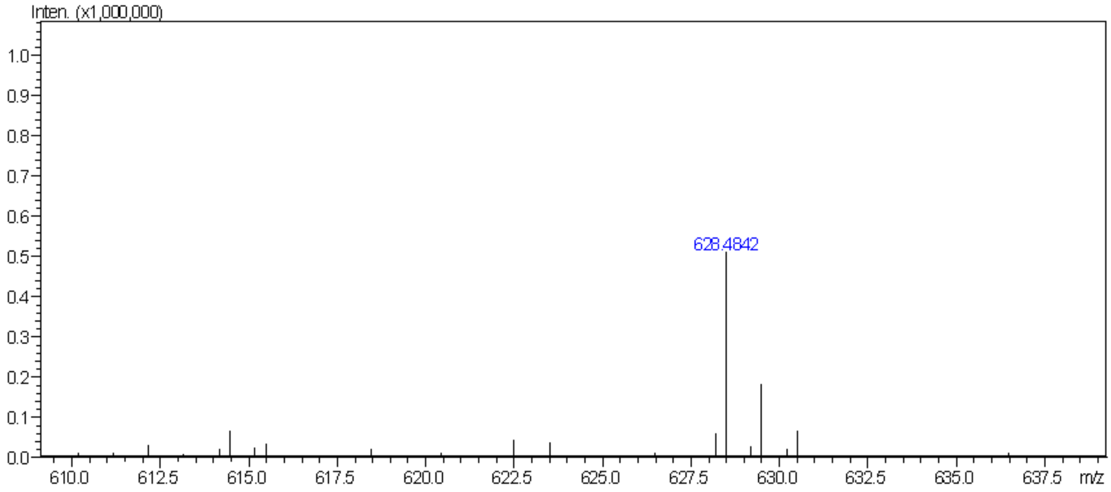

^{1}H NMR spectrum of compound **2i**

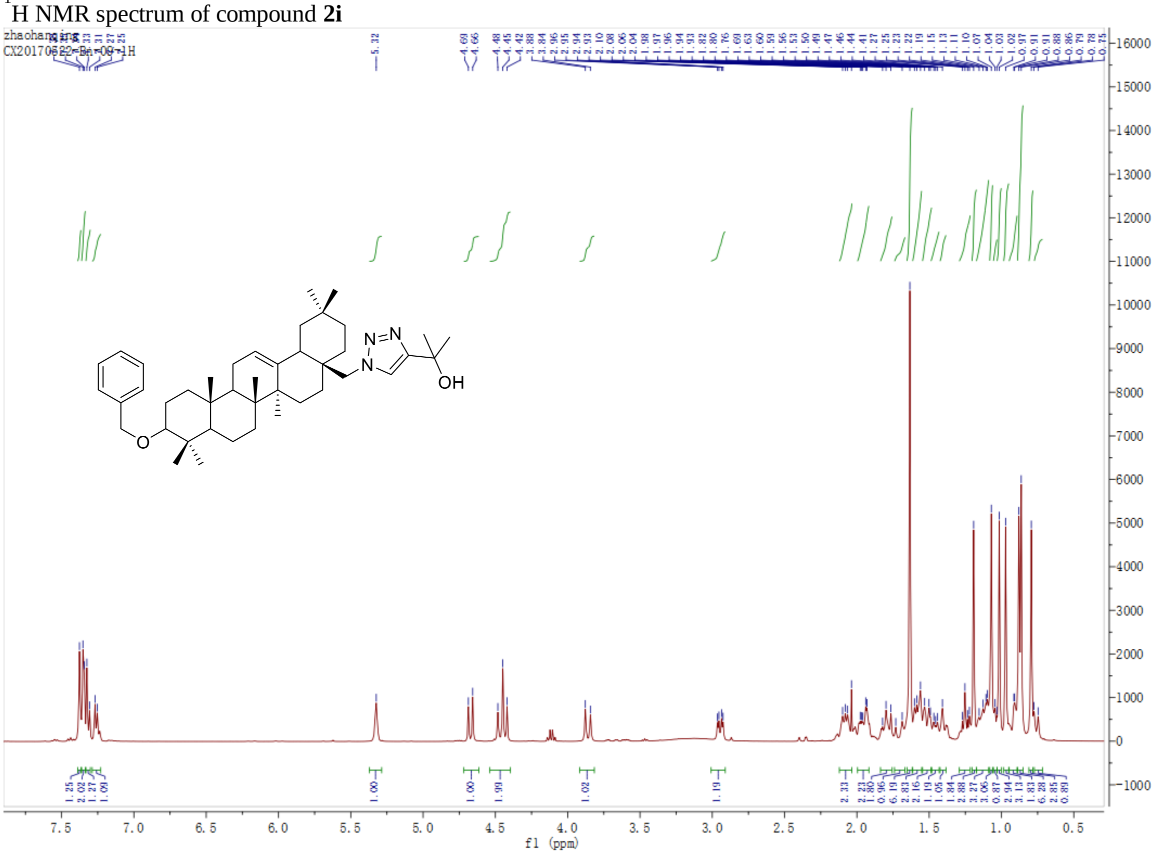

^{13}C NMR spectrum of compound **2i**

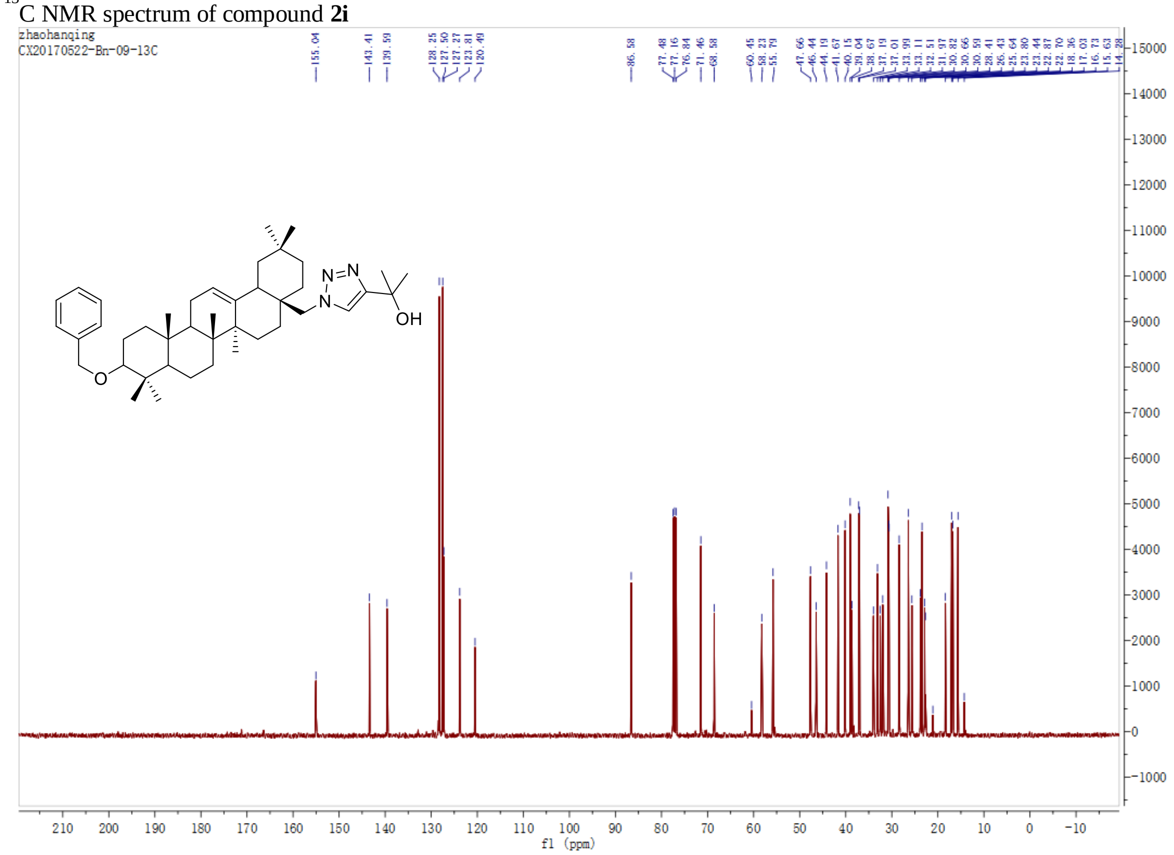

HRMS spectrum of compound **2i**

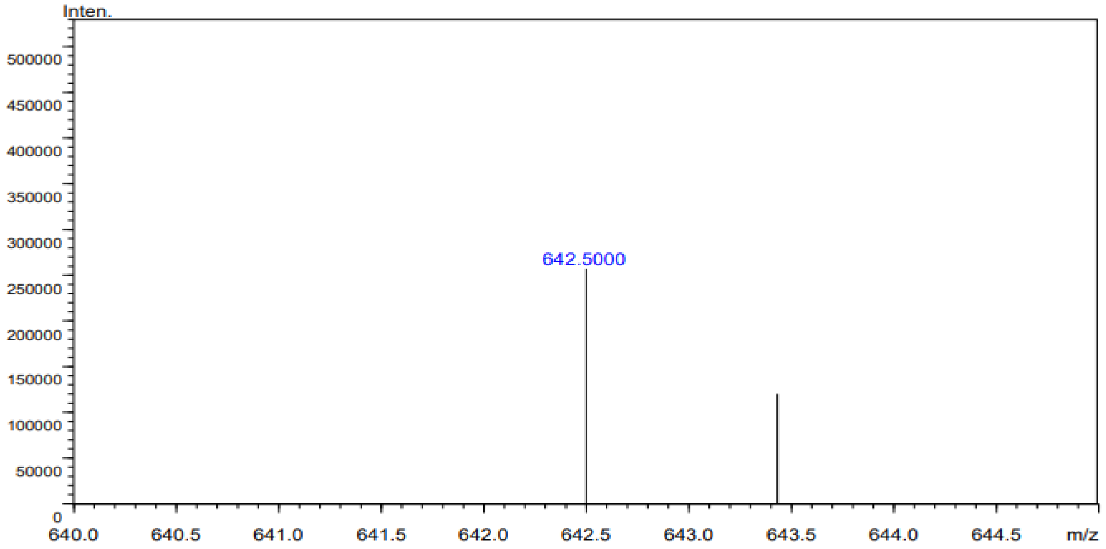

Supplement: Supplementary file 1 [file molecules-27-04928-s001.zip › molecules-1819964-supplementary.pdf]
